# Supplementary material for: HDAC6-dependent deacetylation of SAE2 enhances SUMO1 conjugation for mitotic integrity
Source: EMBO J. 2025 Aug 20;44(19):5537–63. doi: 10.1038/s44318-025-00532-y (PMC12489036; doi:10.1038/s44318-025-00532-y)
Supplement: Supplementary file 1 — Appendix [file 44318_2025_532_MOESM1_ESM.pdf]

APPENDIX FOR

SUMO E1 regulation directs variant bias for mitotic fidelity, Walker et al.

Contents: Appendix Table S1- Antibody details, Page 1.

Appendix Table S1 – Antibody details.

Antibody (clone)	Host	Supplier	Cat. number	Lot number	Use	Conc.	RRID
β-actin	Rabbit	Abcam	Ab8227	GR3215935-1	WB	1:3000	AB_2305186
acK164-SAE2 (30E2-2)	Mouse	GenScript	Custom design	NA-6	WB	1:1000	N/A
SAE1	Rabbit	Abcam	ab185552	GR3379016-1	WB	1:2500	-
SAE2	Rabbit	Sigma	HPA041436	R38238	WB	1:2500	AB_2677479
UBC9	Rabbit	Abcam	ab75854	GR118836-6	WB	1:2500	AB_1310787
SUMO1 (Y299)	Rabbit	Abcam	ab32058	GR3244068-3; GR3366977-1; GR3244068-3; GR3366977-9;	WB/IP	1:5000	AB_778173
SUMO1 (EP298)	Rabbit	Abcam	ab133352	GR268526-16; GR268526-12; GR268526-10	WB	1:500	AB_11156108
SUMO2/3 (8A2)	Mouse	Abcam	ab81371	GR3379145-7	WB/IP	1:1000	AB_1658424
His	Mouse	Sigma	H1029	025M4780V	WB	1:5000	AB_260015
FLAG (M2)	Mouse	Sigma Aldrich	F1804	SLBT7654	WB	1:1000	AB_262044
GFP	Mouse	Roche	11814460001	47859600	WB	1:2500	AB_390913
γH2AX	Rabbit	Abcam	b2893ab2893	GR3242597-1	IF	1:2000	AB_303388
αTubulin	Mouse	Santa Cruz	sc-5286	H0613	WB	1:1000	AB_628411
CENPA	Mouse	Invitrogen	MA1-20832	YL4140131	IF	1:500	AB_2078763
NuMA	Mouse	Santa Cruz	sc-365532	A3124<br>C1323	WB<br>IF	1:1000<br>1:2000	AB_10846197
Vinculin [EPR8185]	Rabbit	Abcam	ab129002	GR221671-50	WB	1:2000	AB_11144129
Pericentrin	Rabbit	Abcam	ab4448	GR245491-2	IF	1:1000	AB_304461
αTubulin	Mouse	Novus	NB100-690	G-3	IF	1:500	AB_521686
pS10-H3	Mouse	Invitrogen	MA5-15220	XJ3742465	WB	1:5000	AB_11008586
pS10-H3	Rabbit	Antibodies.com	A94899	32699	WB	1:5000	-
Donkey α Mouse AlexaFluor 488	Donkey	Life Technologies	A21202	1975519	IF	1:5000	AB_141607
Donkey α Rabbit AlexaFluor 488	Donkey	Life Technologies	A21206	1874771	IF	1:5000	AB_2535792
Donkey α Mouse AlexaFluor 555	Donkey	Life Technologies	A31570	1774719	IF	1:5000	AB_2536180
Donkey α Rabbit AlexaFluor 555	Donkey	Life Technologies	A31572	1945911	IF	1:5000	AB_162543
Donkey α rat AlexaFluor 555	Donkey	Life Technologies	A21434	1987272	IF	1:5000	AB_2535855
Rabbit α Mouse HRP	Rabbit	Dako	P0161	20062080	WB	1:10000	AB_2687969
Swine α Rabbit HRP	Swine	Dako	P0217	20047666	WB	1:10000	AB_2728719
Mouse TrueBlot® ULTRA: Anti-Mouse Ig HRP	Rat	Rockland	18-8817-30	39891	WB	1:5000	AB_2610849
