# Supplementary material for: HDAC6-dependent deacetylation of SAE2 enhances SUMO1 conjugation for mitotic integrity
Source: EMBO J. 2025 Aug 20;44(19):5537–63. doi: 10.1038/s44318-025-00532-y (PMC12489036; doi:10.1038/s44318-025-00532-y)
Supplement: Supplementary file 3 — Figure 1 raw data [file 44318_2025_532_MOESM3_ESM.zip › Figure 1/1A/figure 1a blots.pptx]

## Slide 1
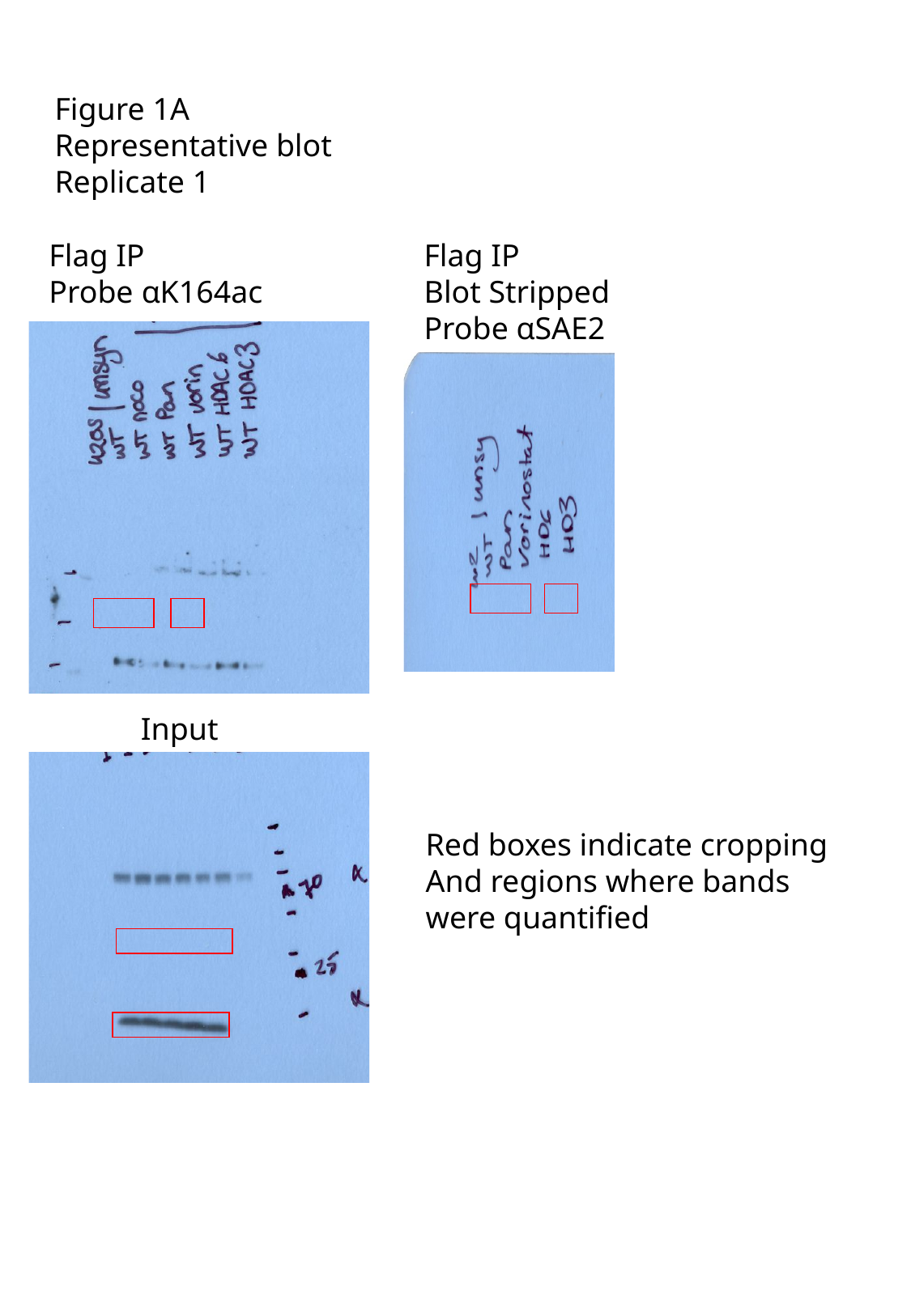

Figure 1A
Representative blot
Replicate 1
Flag IP
Blot Stripped
Probe αSAE2
Flag IP
Probe αK164ac
Input
Red boxes indicate cropping
And regions where bands were quantified

## Slide 2
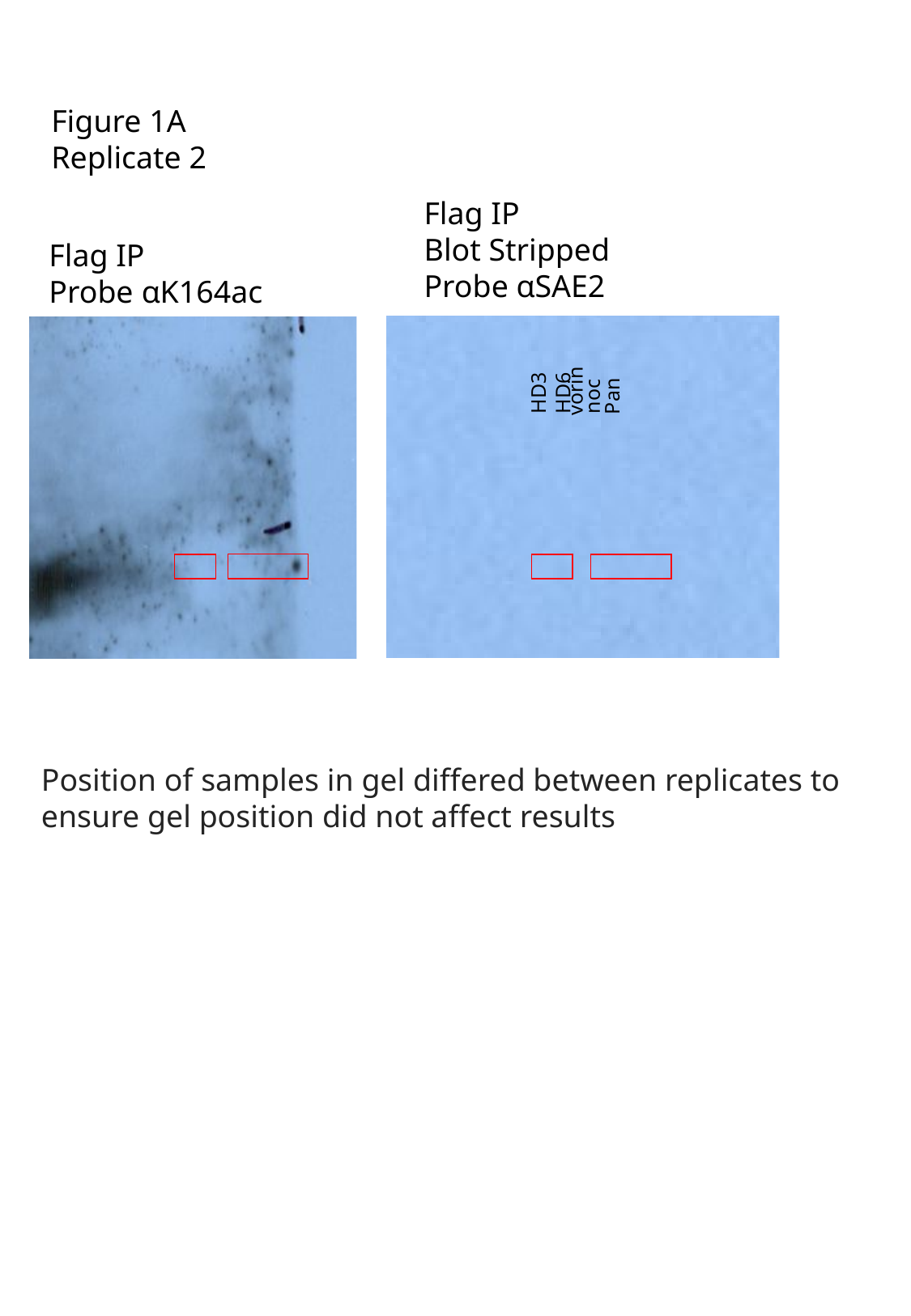

Figure 1A
Replicate 2
Flag IP
Blot Stripped
Probe αSAE2
Flag IP
Probe αK164ac
vorin
HD3
HD6
noc
Pan
Position of samples in gel differed between replicates to ensure gel position did not affect results

## Slide 3
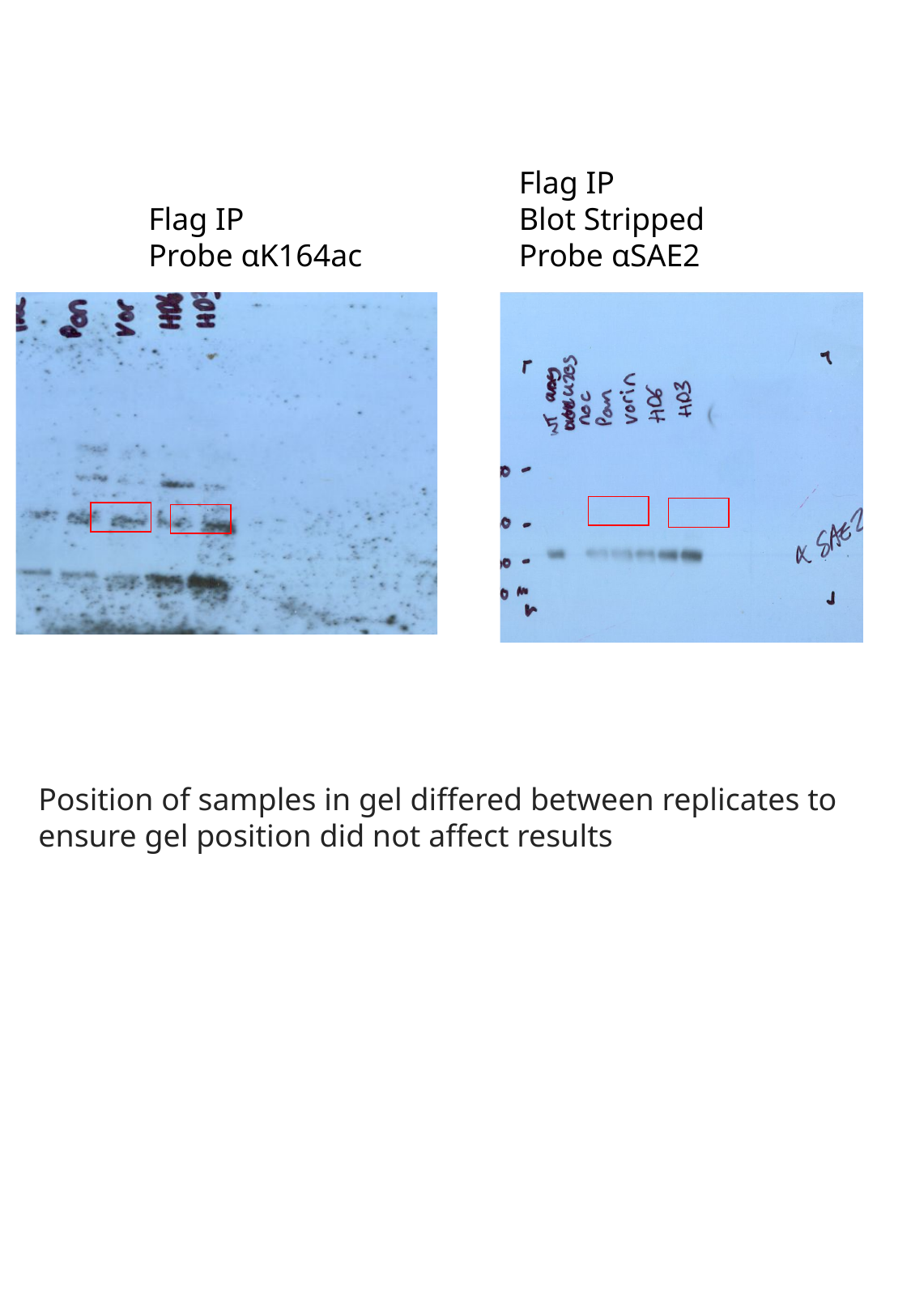

Flag IP
Blot Stripped
Probe αSAE2
Flag IP
Probe αK164ac
Position of samples in gel differed between replicates to ensure gel position did not affect results
