# Supplementary material for: HDAC6-dependent deacetylation of SAE2 enhances SUMO1 conjugation for mitotic integrity
Source: EMBO J. 2025 Aug 20;44(19):5537–63. doi: 10.1038/s44318-025-00532-y (PMC12489036; doi:10.1038/s44318-025-00532-y)
Supplement: Supplementary file 3 — Figure 1 raw data [file 44318_2025_532_MOESM3_ESM.zip › Figure 1/1C/Figure 1C acetyltransferase inhibitor IP.pptx]

## Slide 1
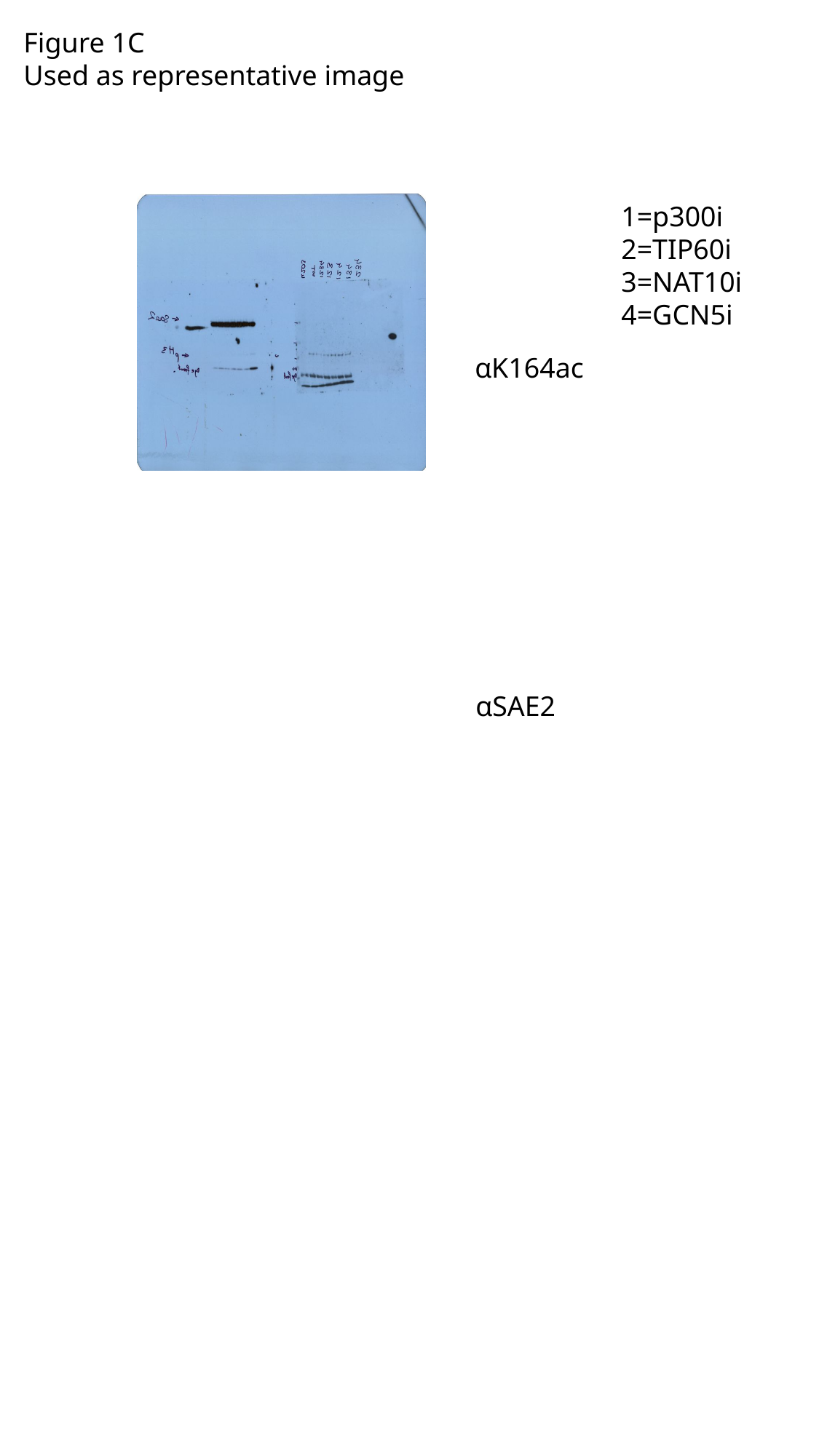

Figure 1C
Used as representative image
1=p300i
2=TIP60i
3=NAT10i
4=GCN5i
αK164ac
αSAE2

## Slide 2
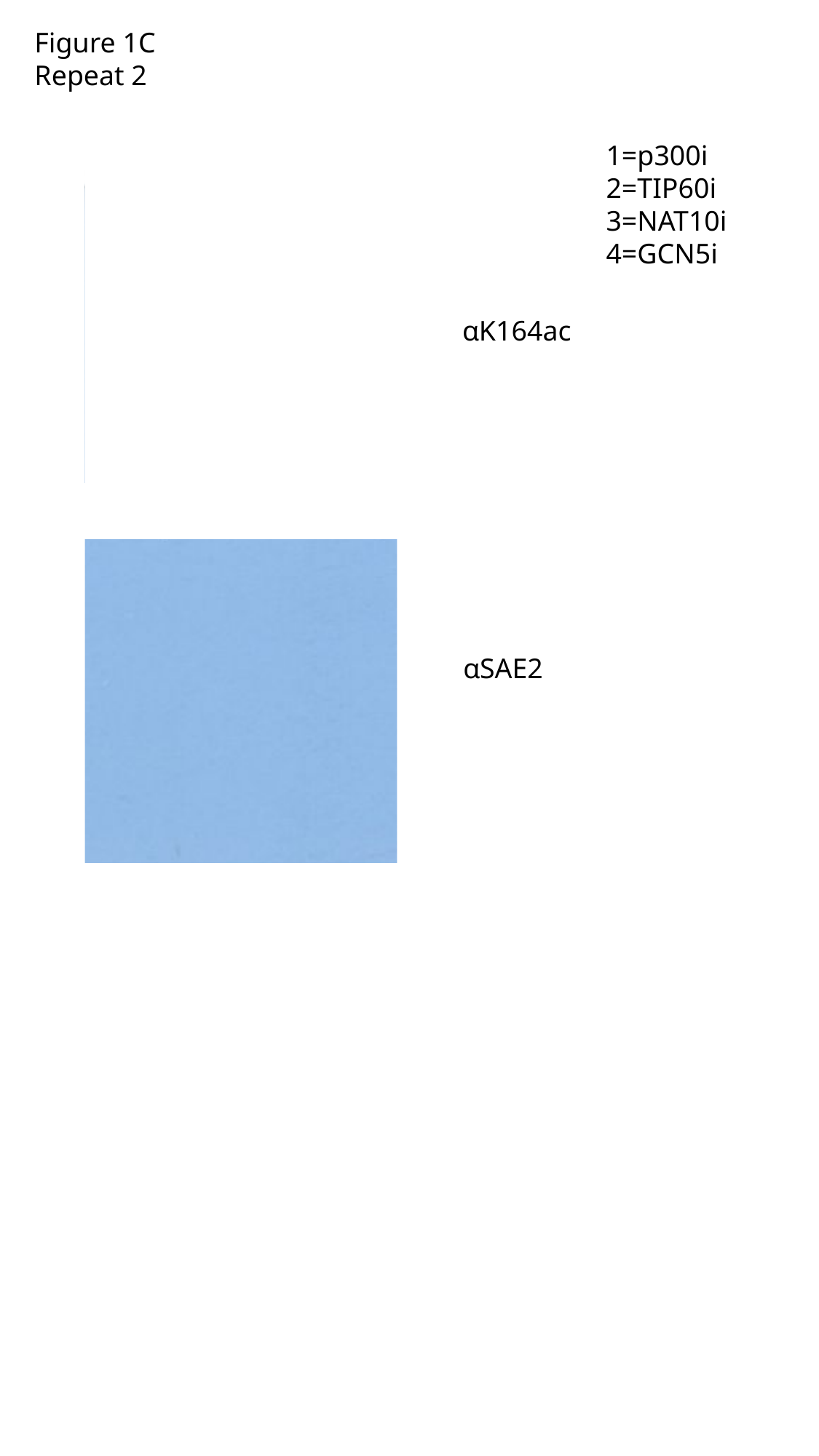

Figure 1C
Repeat 2
1=p300i
2=TIP60i
3=NAT10i
4=GCN5i
αK164ac
αSAE2

## Slide 3
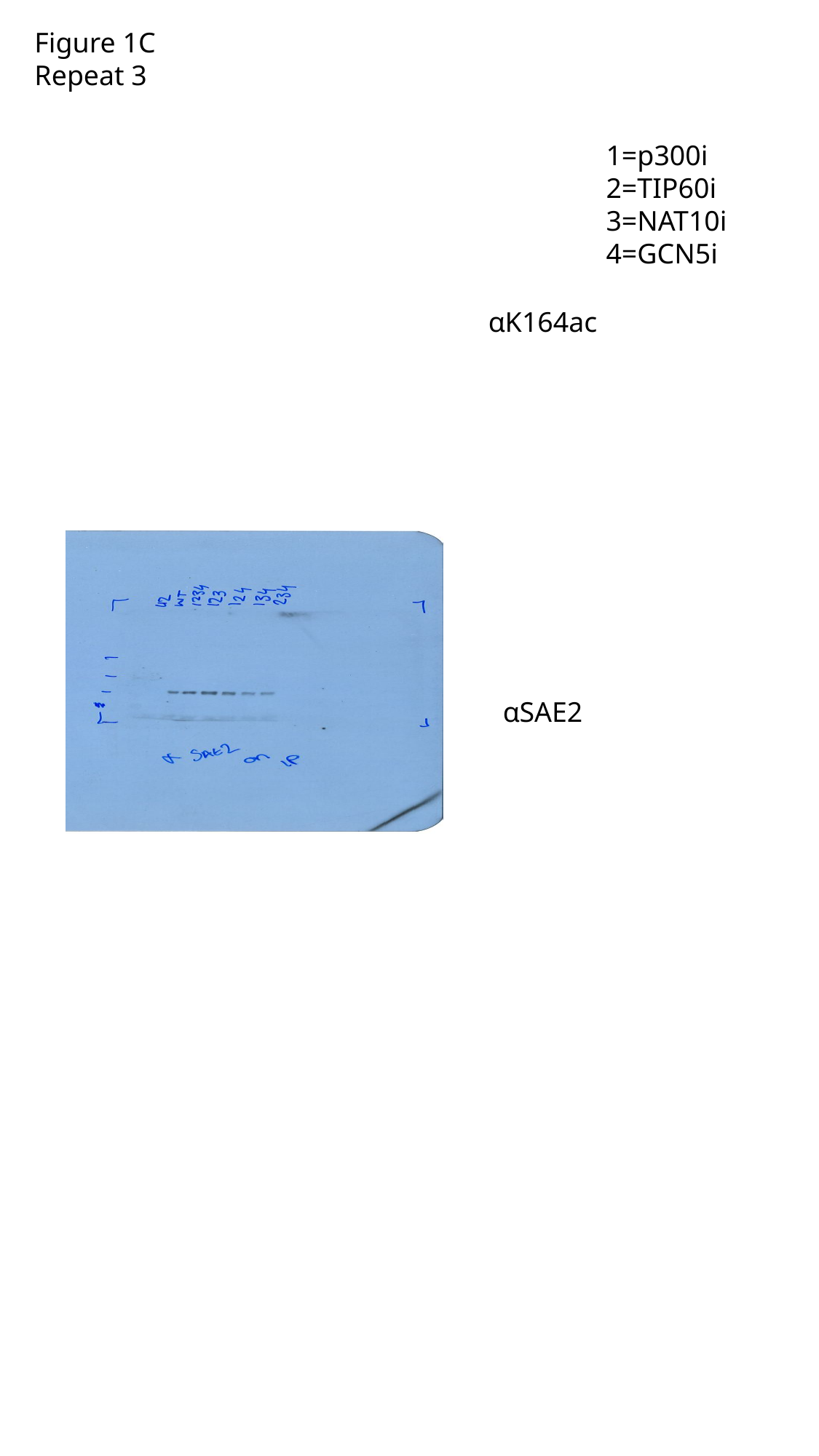

Figure 1C
Repeat 3
1=p300i
2=TIP60i
3=NAT10i
4=GCN5i
αK164ac
αSAE2
