# Supplementary figures and images for: HDAC6-dependent deacetylation of SAE2 enhances SUMO1 conjugation for mitotic integrity (part 1 of 2)
Source: EMBO J. 2025 Aug 20;44(19):5537–63. doi: 10.1038/s44318-025-00532-y (PMC12489036; doi:10.1038/s44318-025-00532-y)

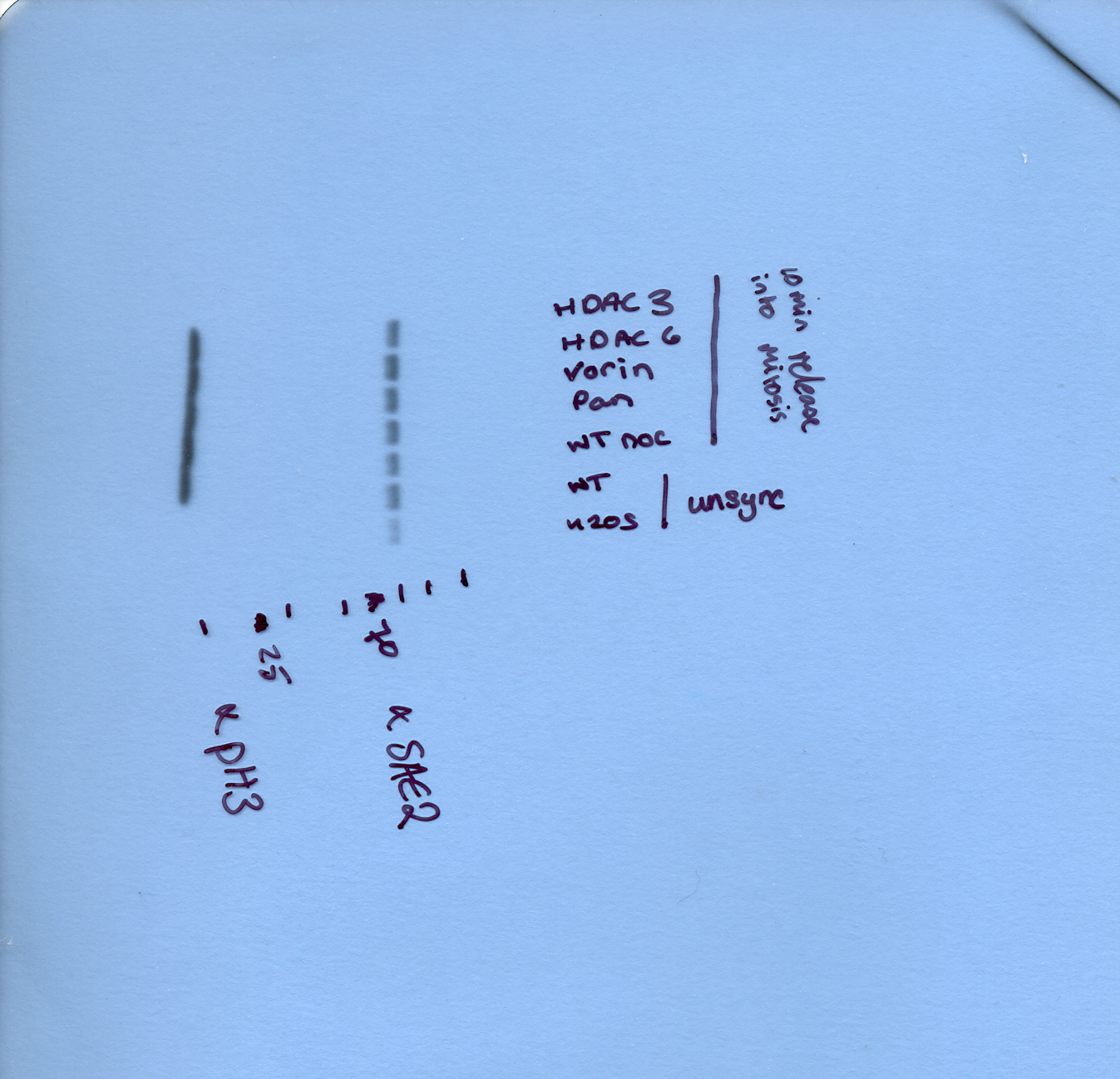

Supplement: Supplementary file 3 — Figure 1 raw data [file 44318_2025_532_MOESM3_ESM.zip › Figure 1/1A/figure 1a loading control probe SAE2 phosH3.tif]

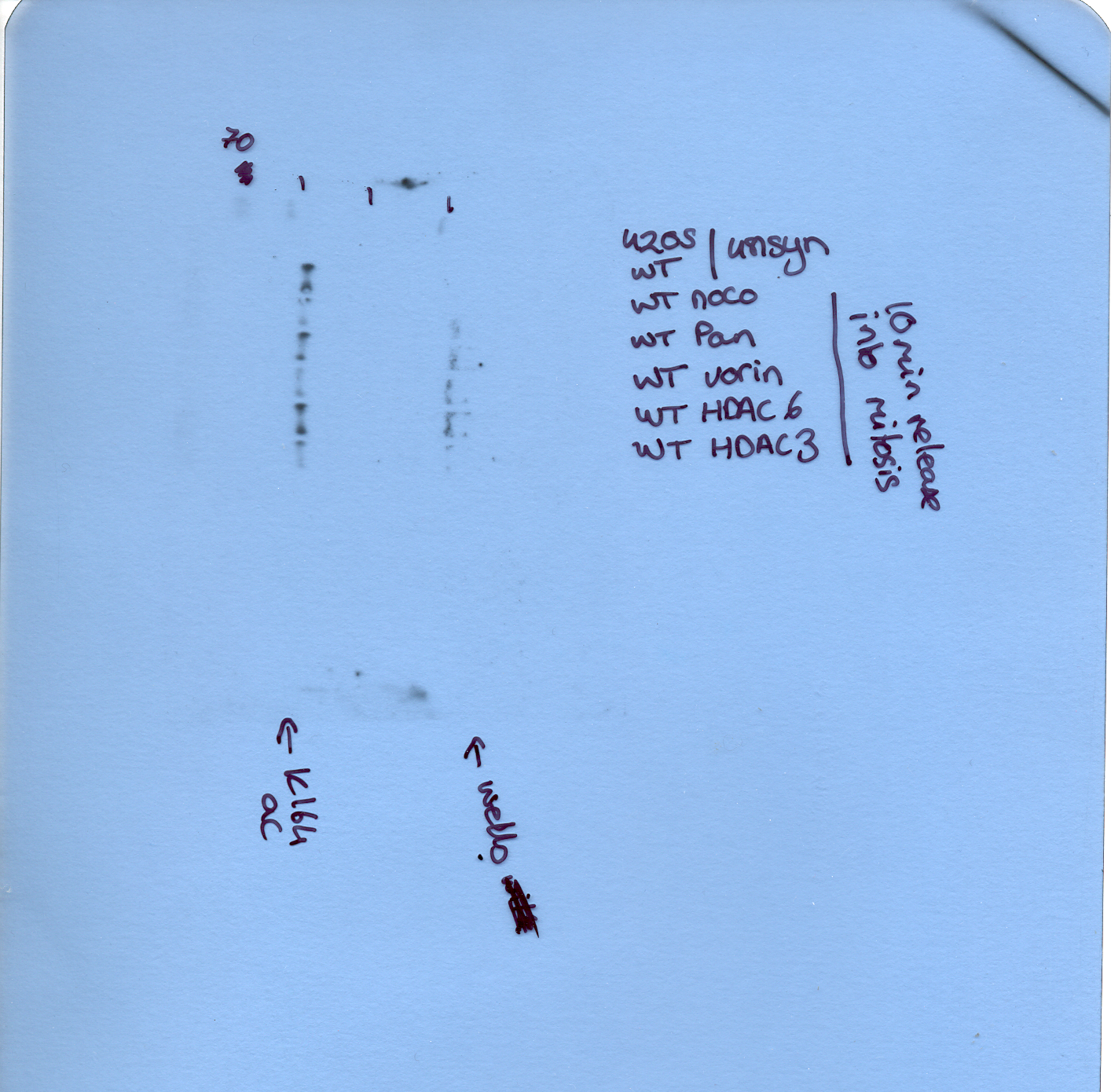

Supplement: Supplementary file 3 — Figure 1 raw data [file 44318_2025_532_MOESM3_ESM.zip › Figure 1/1A/figure 1a probe ack164 replicate 1.tif]

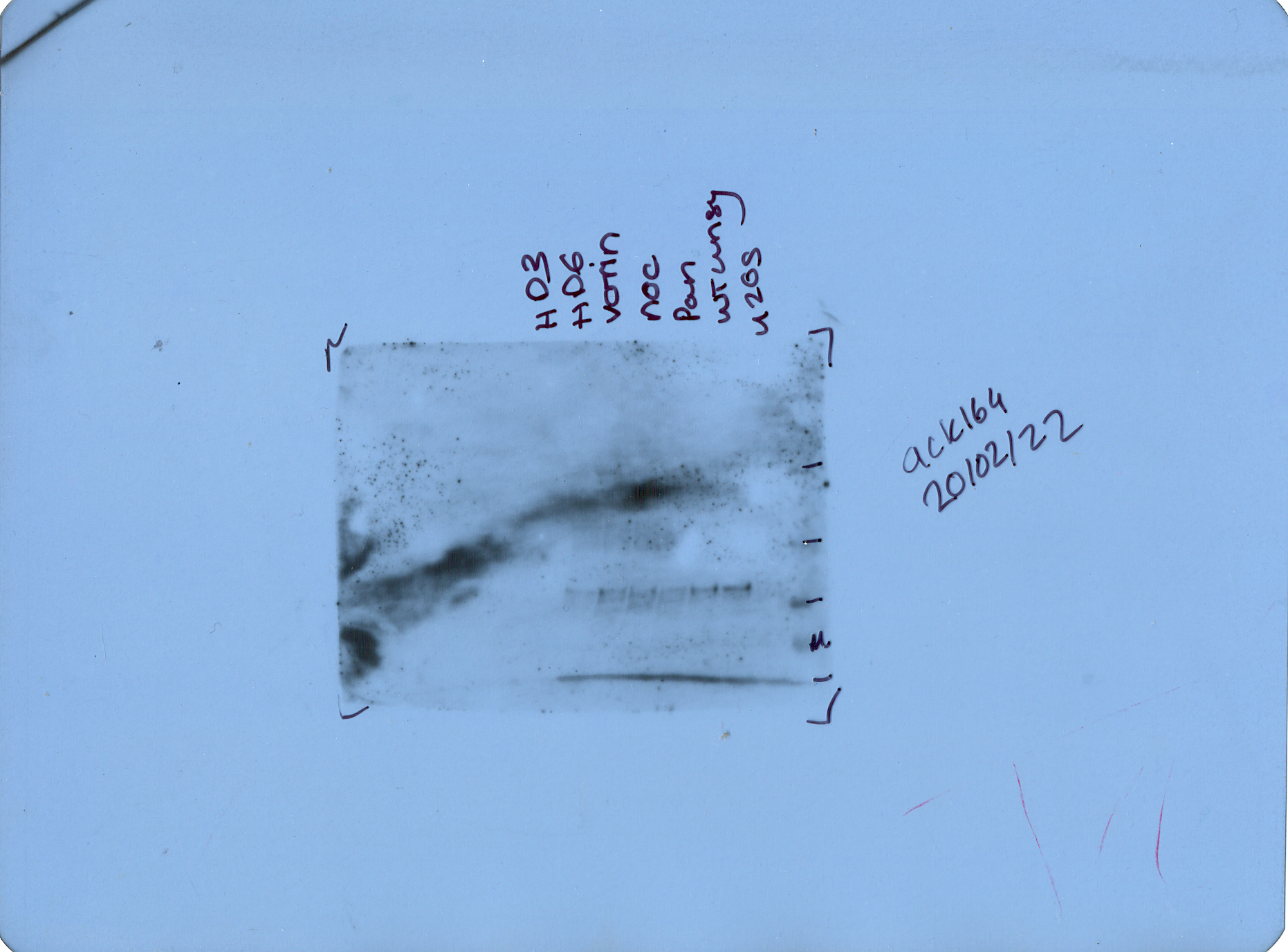

Supplement: Supplementary file 3 — Figure 1 raw data [file 44318_2025_532_MOESM3_ESM.zip › Figure 1/1A/figure 1a probe acK164 replicate 2.tif]

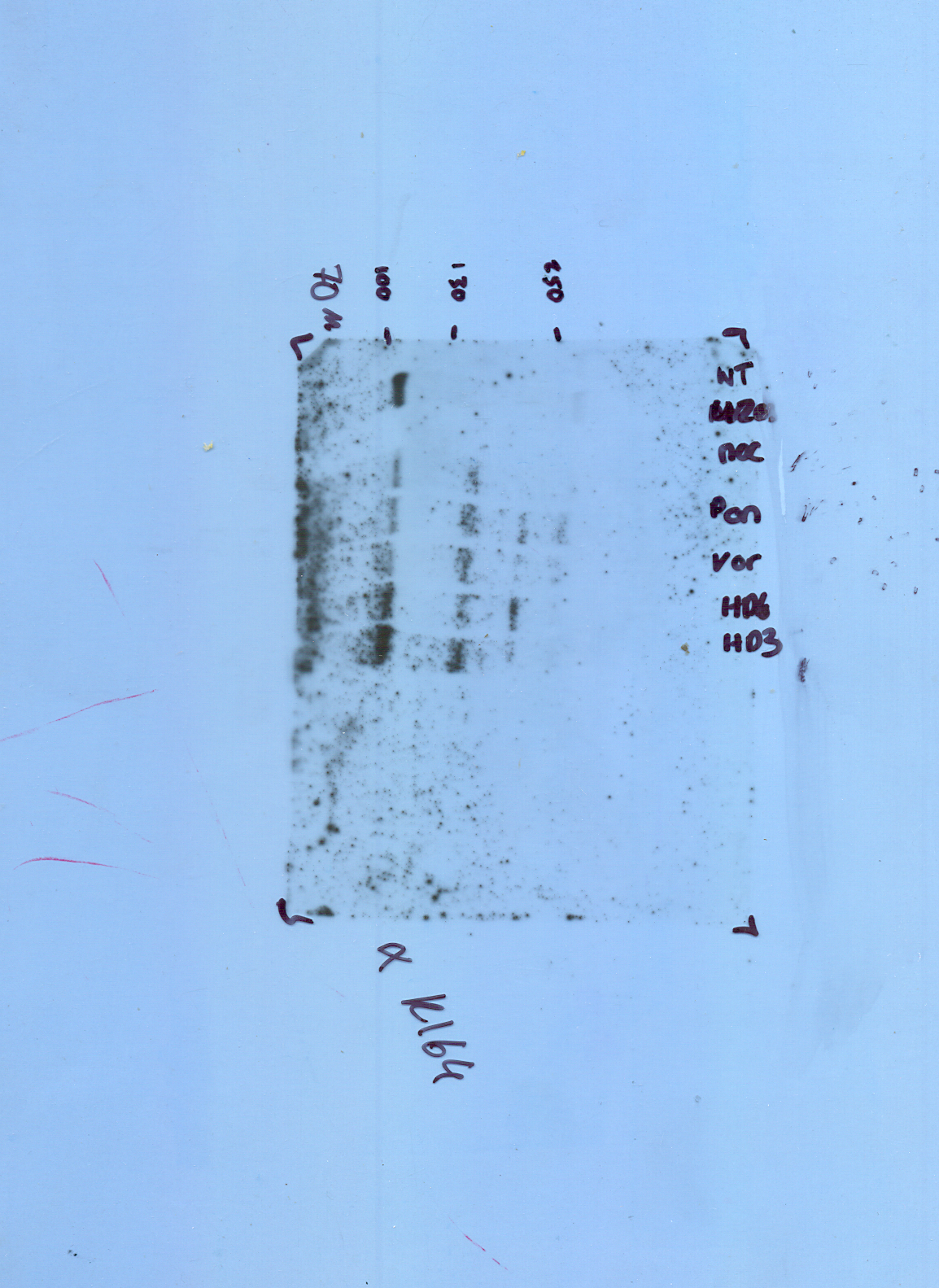

Supplement: Supplementary file 3 — Figure 1 raw data [file 44318_2025_532_MOESM3_ESM.zip › Figure 1/1A/figure 1a probe acK164 replicate 3.tif]

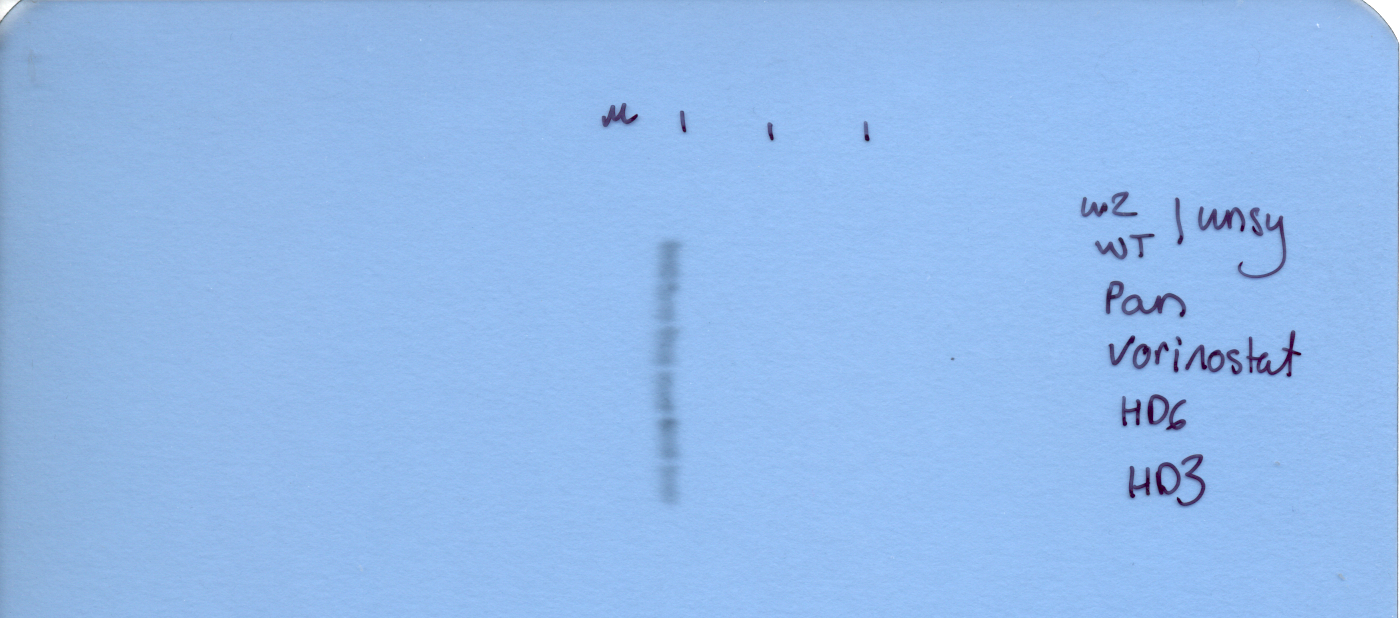

Supplement: Supplementary file 3 — Figure 1 raw data [file 44318_2025_532_MOESM3_ESM.zip › Figure 1/1A/figure 1a probe SAE2 replicate 1.tif]

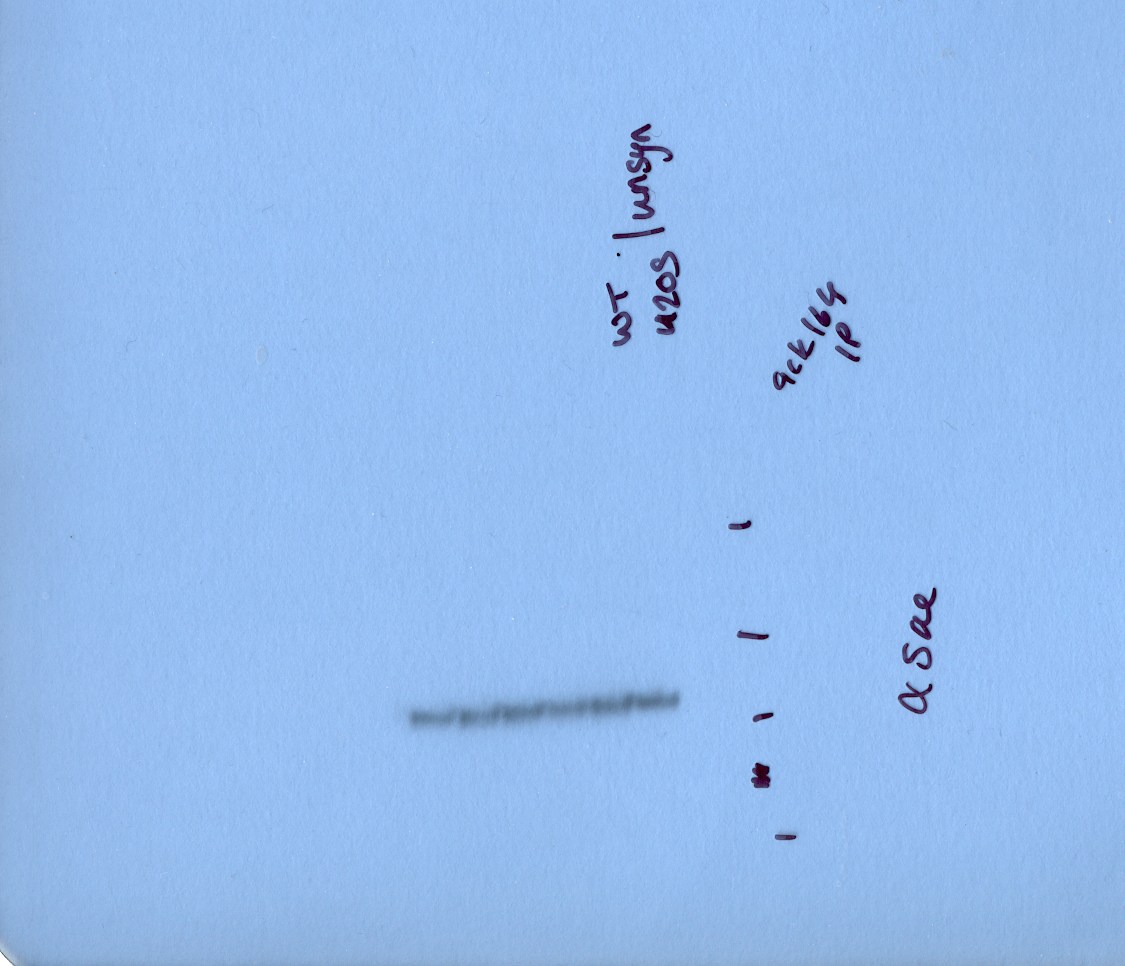

Supplement: Supplementary file 3 — Figure 1 raw data [file 44318_2025_532_MOESM3_ESM.zip › Figure 1/1A/figure 1a probe SAE2 replicate 2.tif]

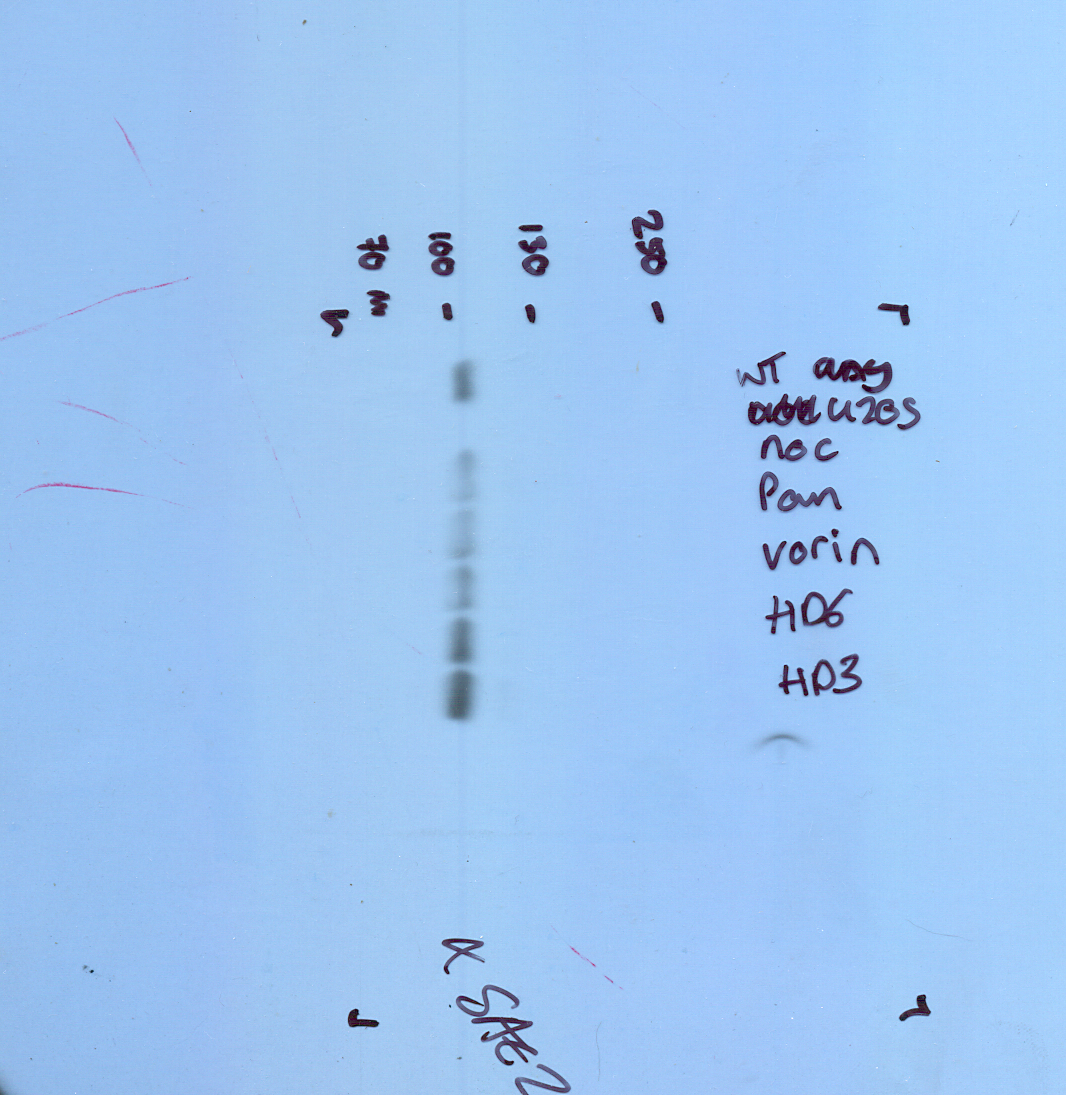

Supplement: Supplementary file 3 — Figure 1 raw data [file 44318_2025_532_MOESM3_ESM.zip › Figure 1/1A/figure 1a probe SAE2 replicate 3.tif]

## Slide 1
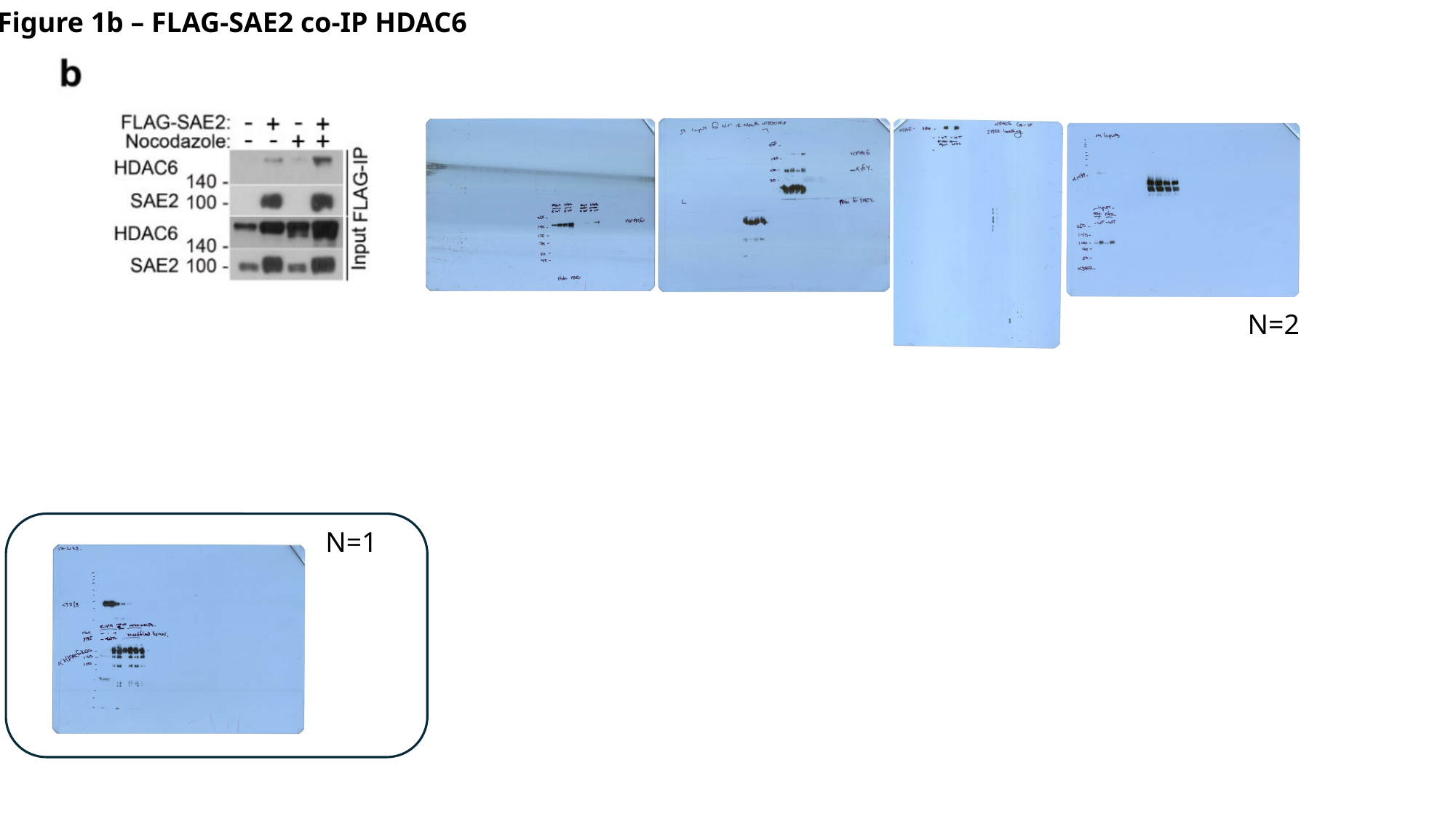

Figure 1b – FLAG-SAE2 co-IP HDAC6
N=2
N=1

Supplement: Supplementary file 3 — Figure 1 raw data [file 44318_2025_532_MOESM3_ESM.zip › Figure 1/1B/Figure 1.pptx]

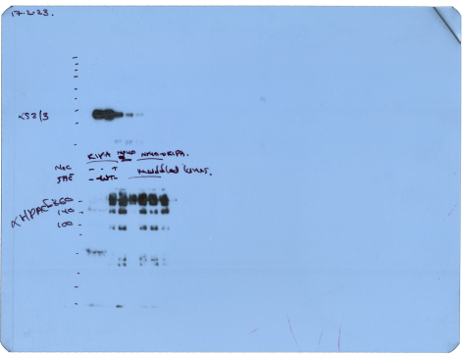

Supplement: Supplementary file 3 — Figure 1 raw data [file 44318_2025_532_MOESM3_ESM.zip › Figure 1/1B/N=1 HDAC6 co-IP.tif]

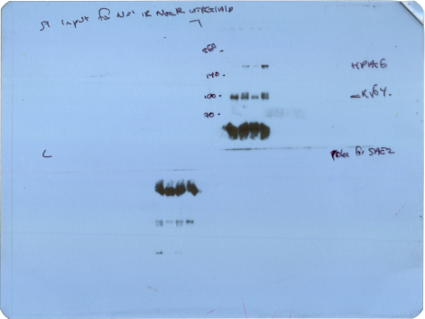

Supplement: Supplementary file 3 — Figure 1 raw data [file 44318_2025_532_MOESM3_ESM.zip › Figure 1/1B/N=2 aHDAC6 IP.tif]

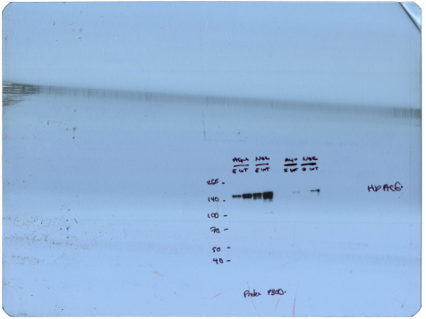

Supplement: Supplementary file 3 — Figure 1 raw data [file 44318_2025_532_MOESM3_ESM.zip › Figure 1/1B/N=2 aHDAC6.tif]

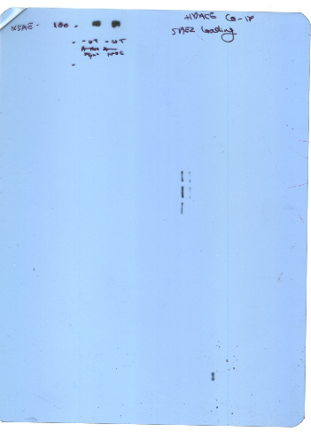

Supplement: Supplementary file 3 — Figure 1 raw data [file 44318_2025_532_MOESM3_ESM.zip › Figure 1/1B/N=2 aSAE2 IP.tif]

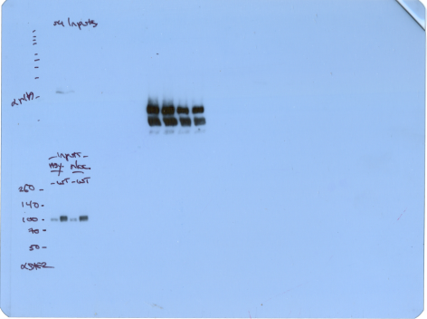

Supplement: Supplementary file 3 — Figure 1 raw data [file 44318_2025_532_MOESM3_ESM.zip › Figure 1/1B/N=2 SAE2 Input.tif]

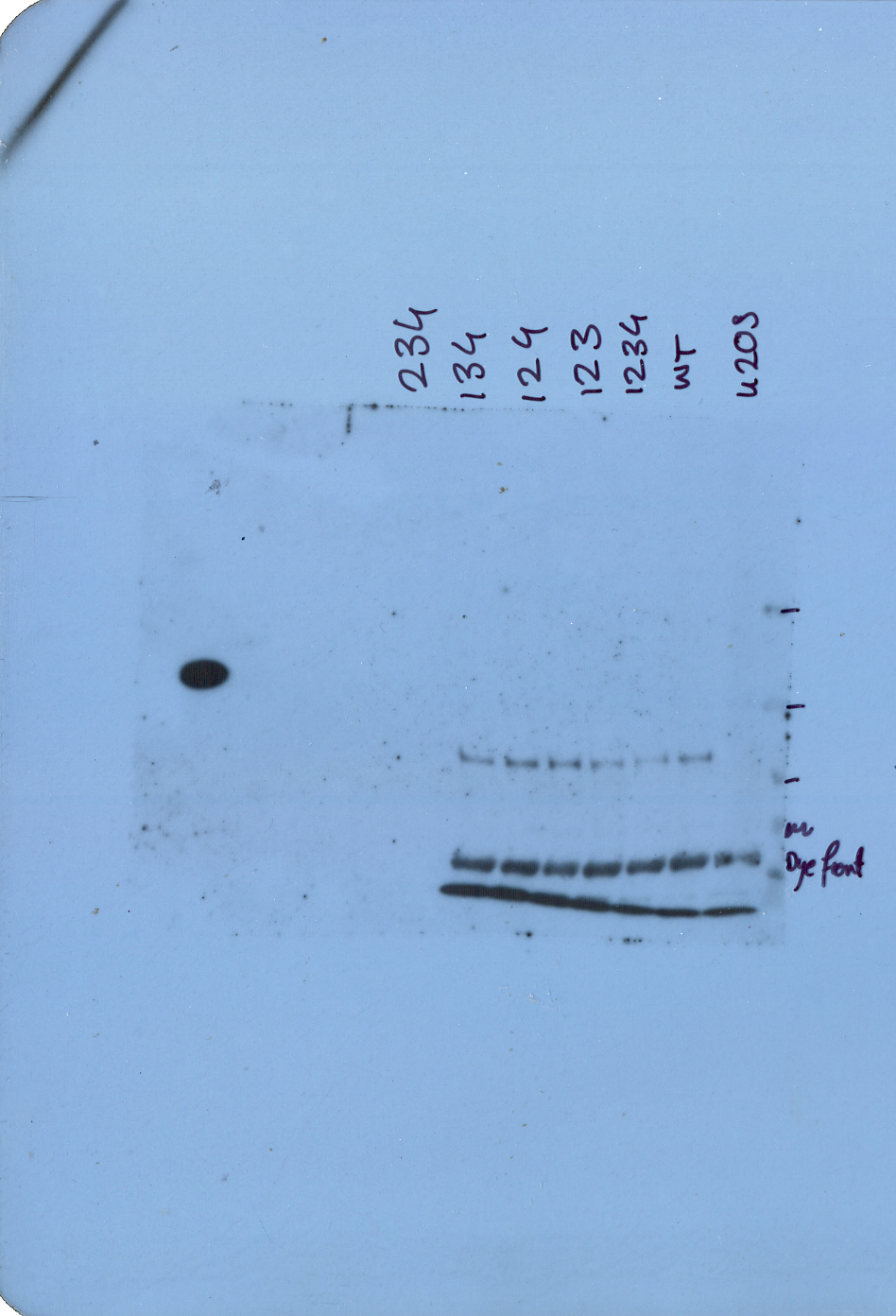

Supplement: Supplementary file 3 — Figure 1 raw data [file 44318_2025_532_MOESM3_ESM.zip › Figure 1/1C/Fig 1C antiK164ac replicate 1 representative image.tif]

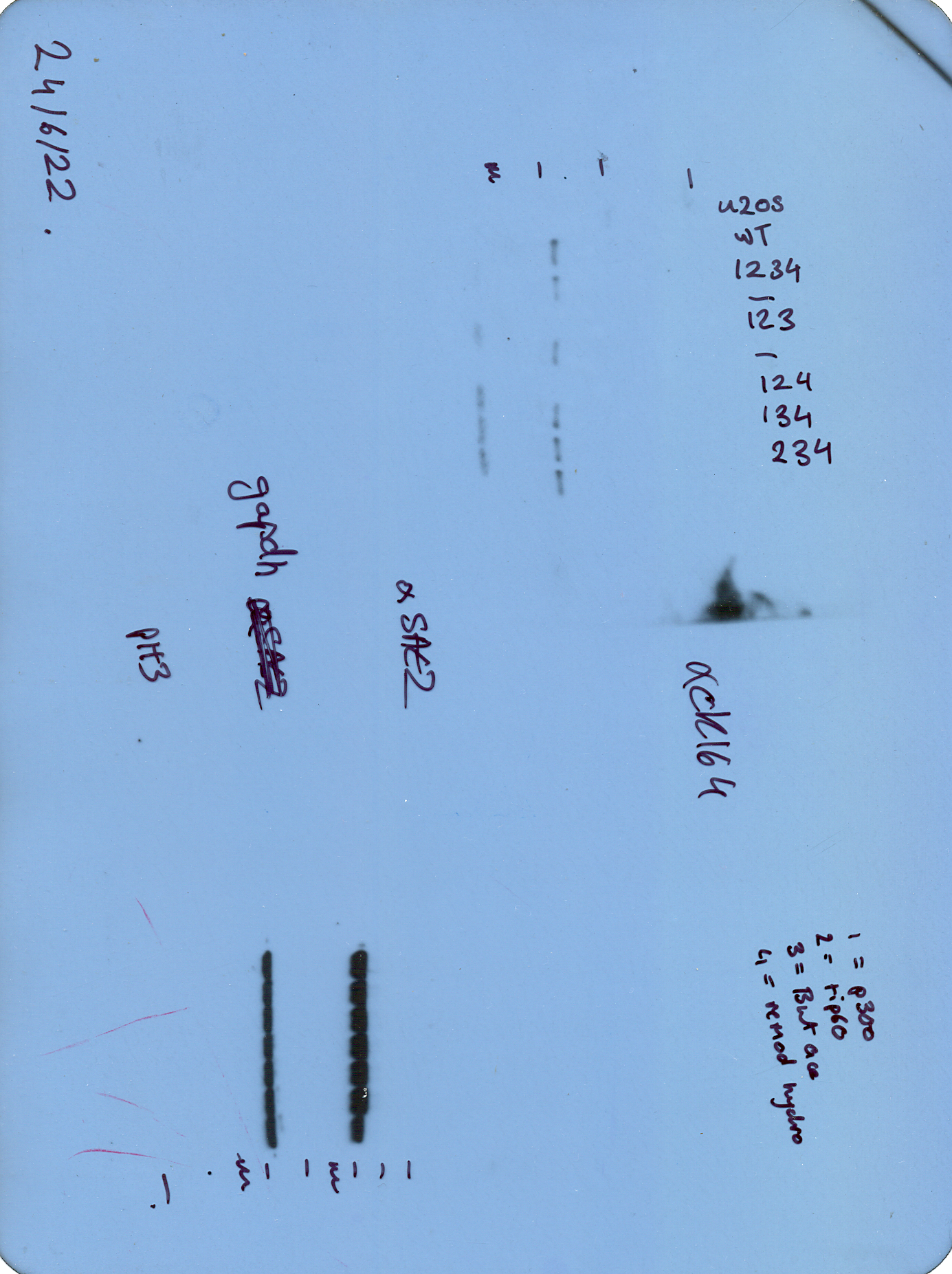

Supplement: Supplementary file 3 — Figure 1 raw data [file 44318_2025_532_MOESM3_ESM.zip › Figure 1/1C/Fig 1C antiK164ac Replicate 2 HATi.tif]

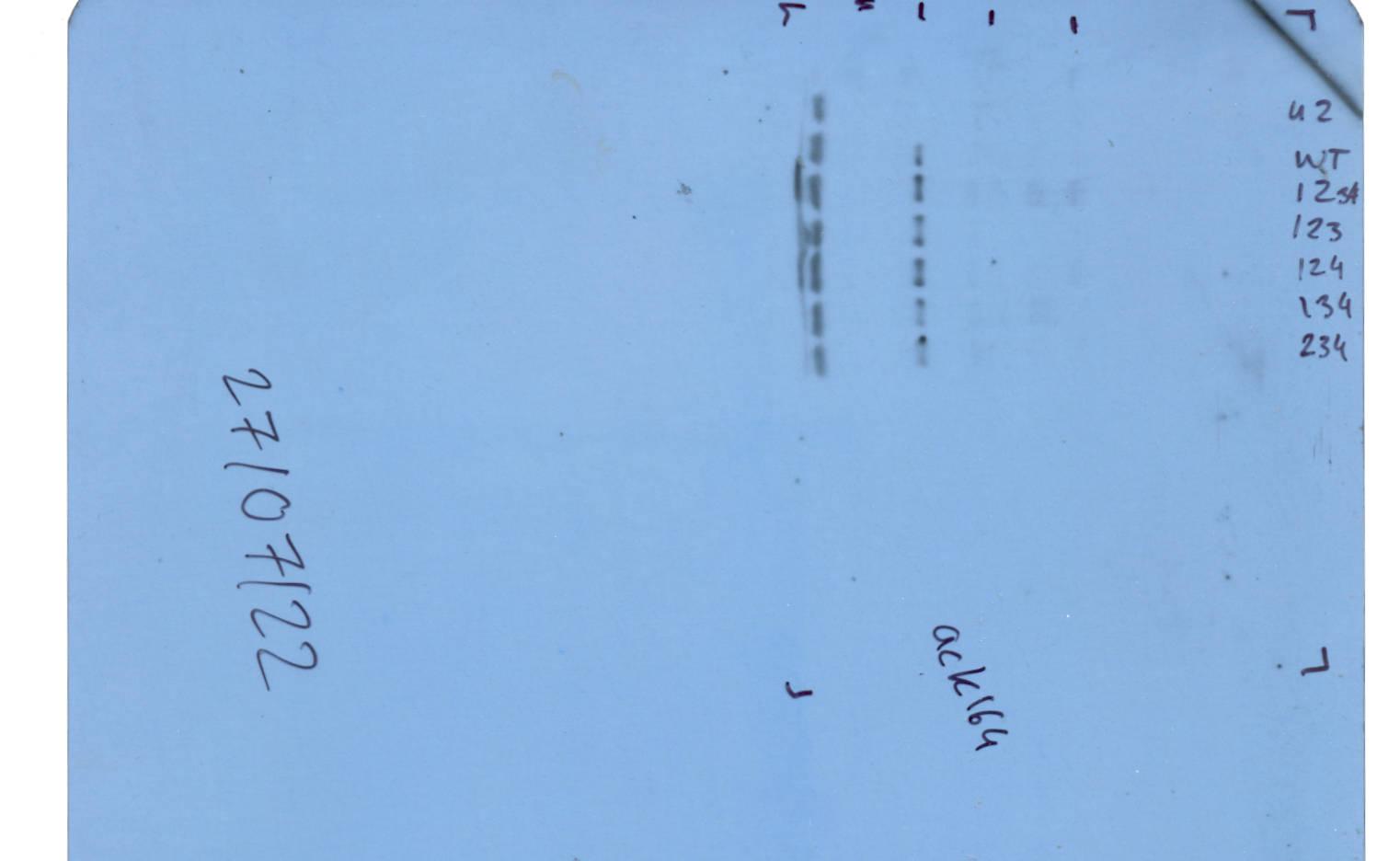

Supplement: Supplementary file 3 — Figure 1 raw data [file 44318_2025_532_MOESM3_ESM.zip › Figure 1/1C/Fig 1C antiK164ac Replicate 3 HATi.tif]

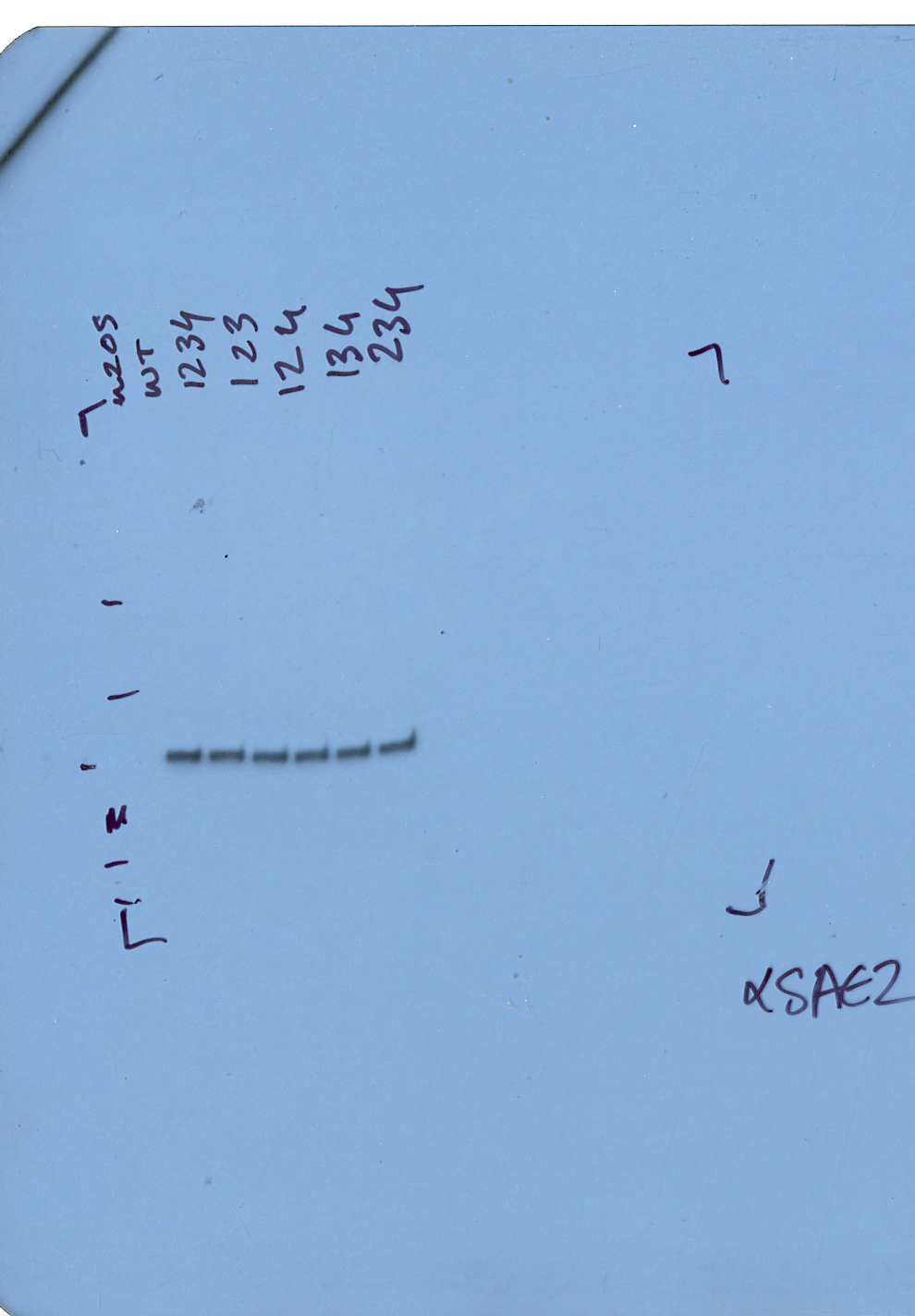

Supplement: Supplementary file 3 — Figure 1 raw data [file 44318_2025_532_MOESM3_ESM.zip › Figure 1/1C/Fig 1C antiSAE2 replicate 1 representative image.tif]

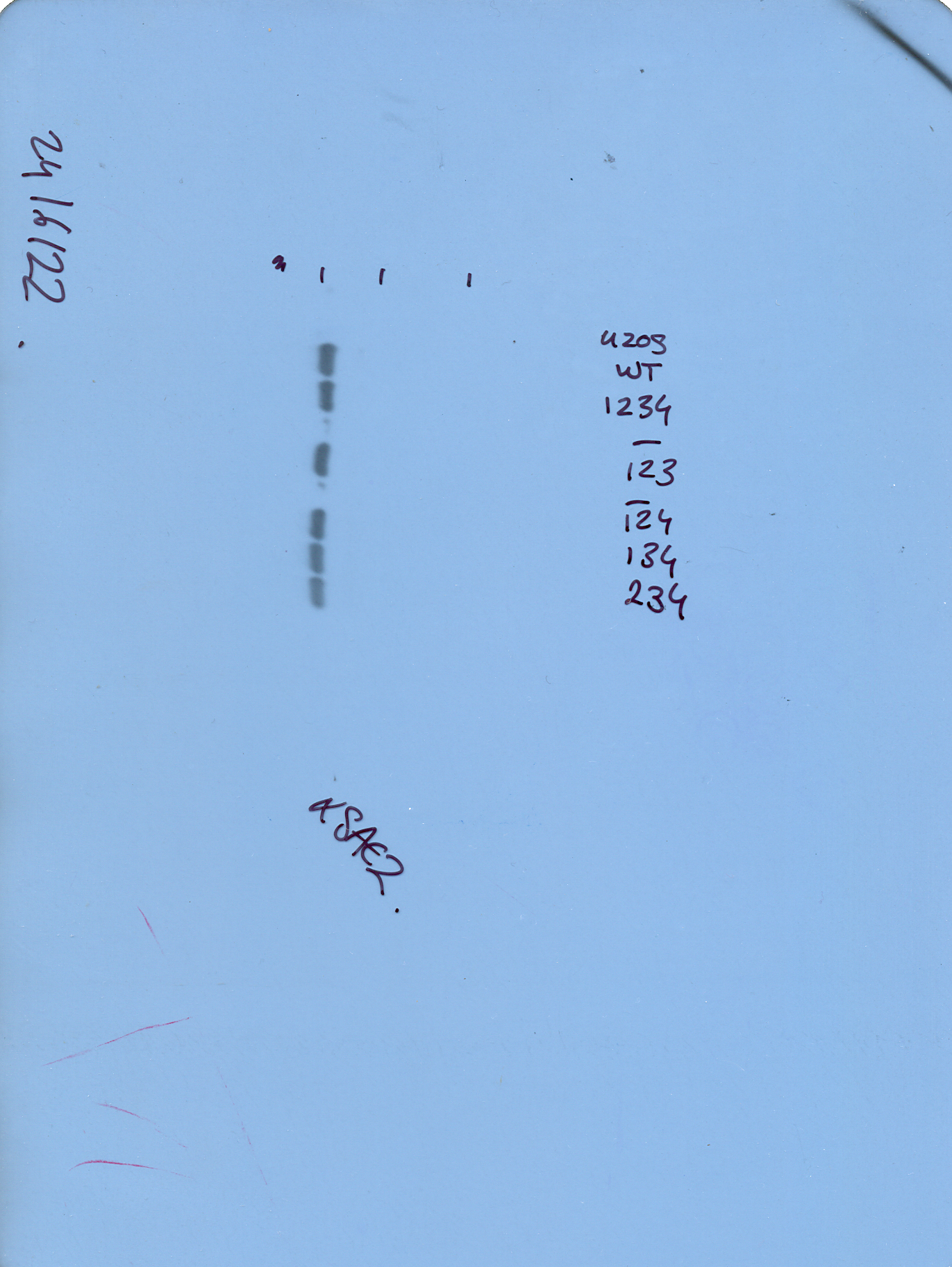

Supplement: Supplementary file 3 — Figure 1 raw data [file 44318_2025_532_MOESM3_ESM.zip › Figure 1/1C/Fig 1C antiSAE2 Replicate 2 HATi.tif]

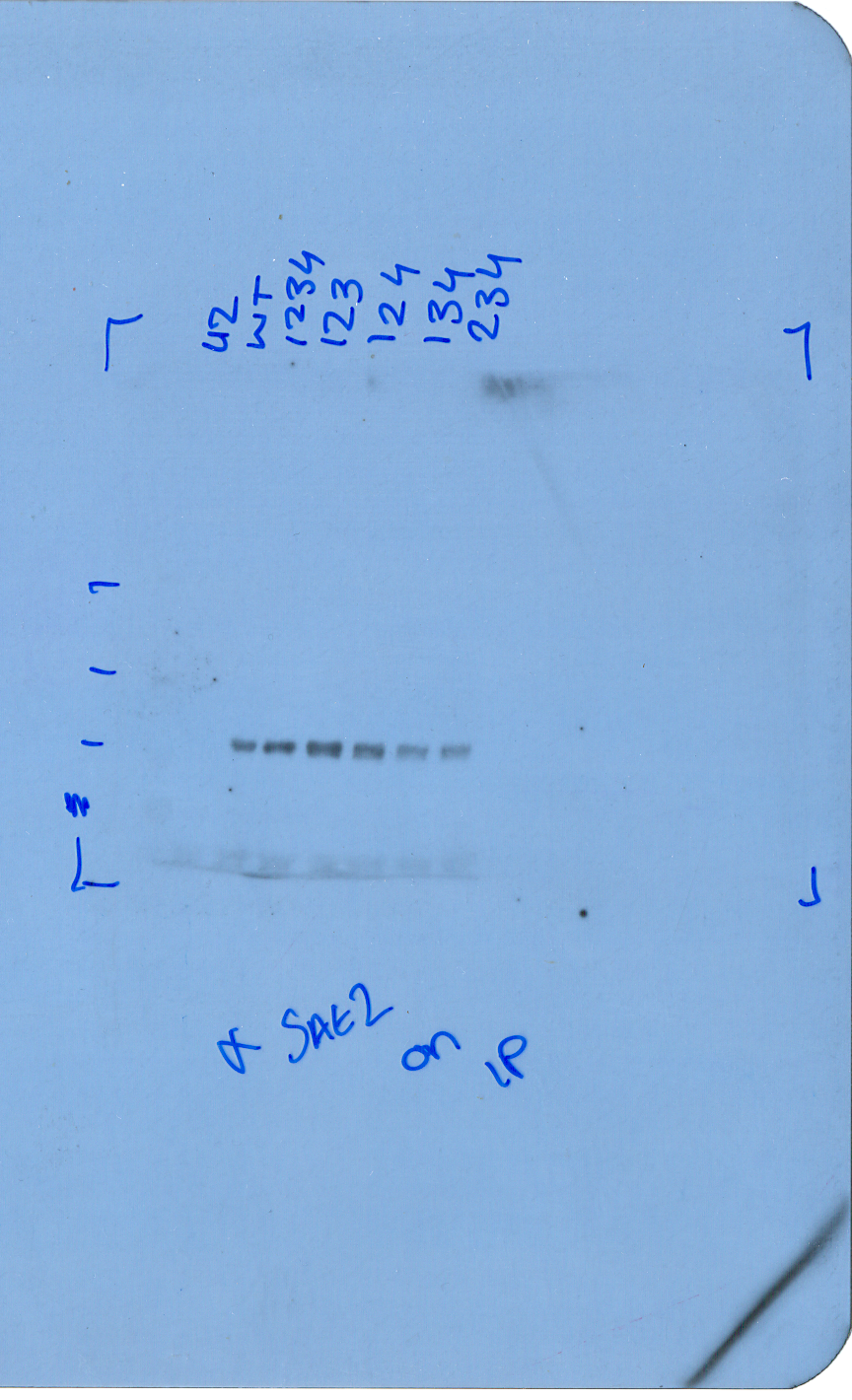

Supplement: Supplementary file 3 — Figure 1 raw data [file 44318_2025_532_MOESM3_ESM.zip › Figure 1/1C/Fig 1C antiSAE2 replicate 3 HATi.tif]

## Slide 1
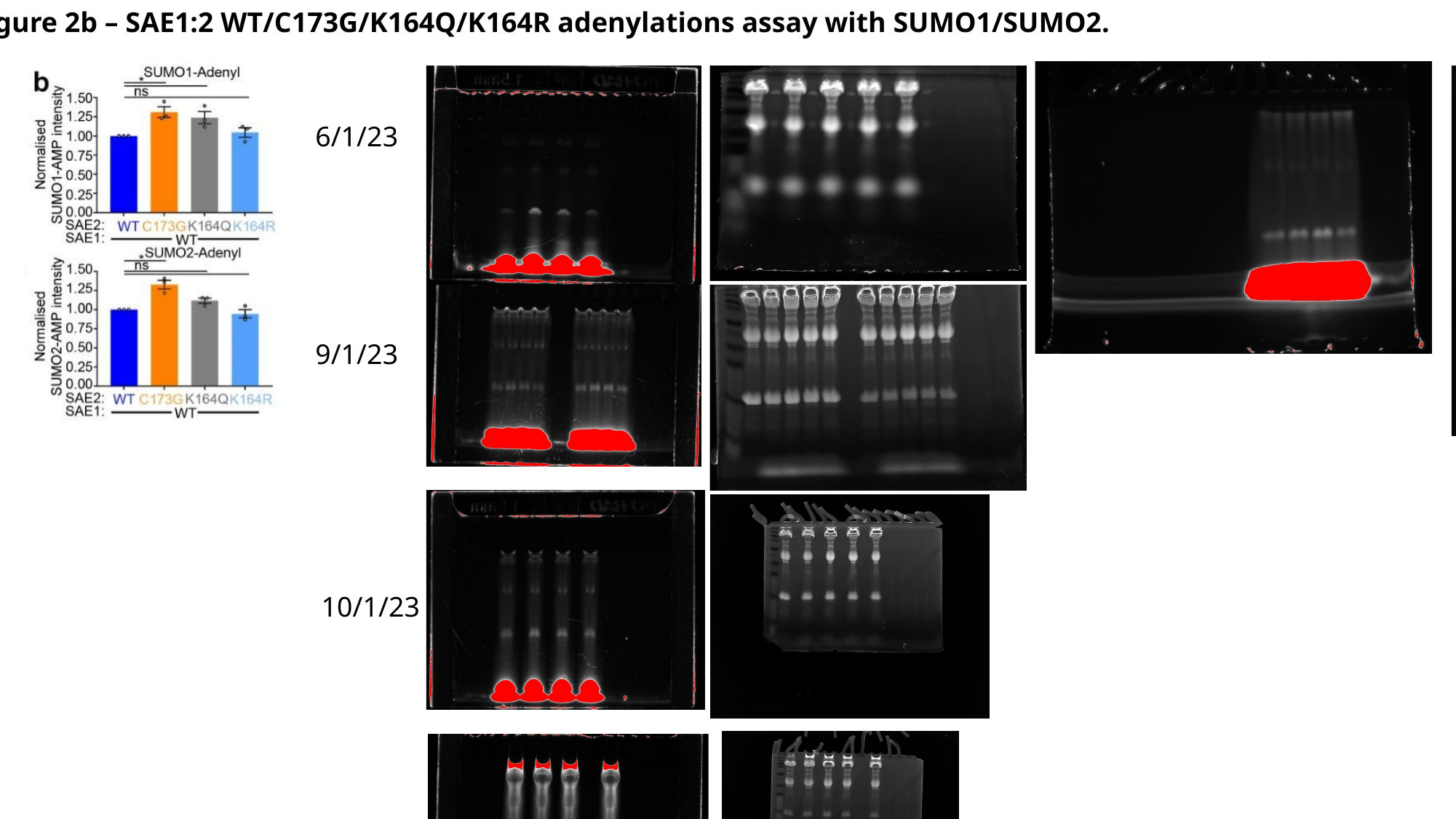

Figure 2b – SAE1:2 WT/C173G/K164Q/K164R adenylations assay with SUMO1/SUMO2.
6/1/23
9/1/23
10/1/23
16/1/23
17/1/23

Supplement: Supplementary file 4 — Figure 2 raw data [file 44318_2025_532_MOESM4_ESM.zip › Figure 2/2B/Figure 2b.pptx]

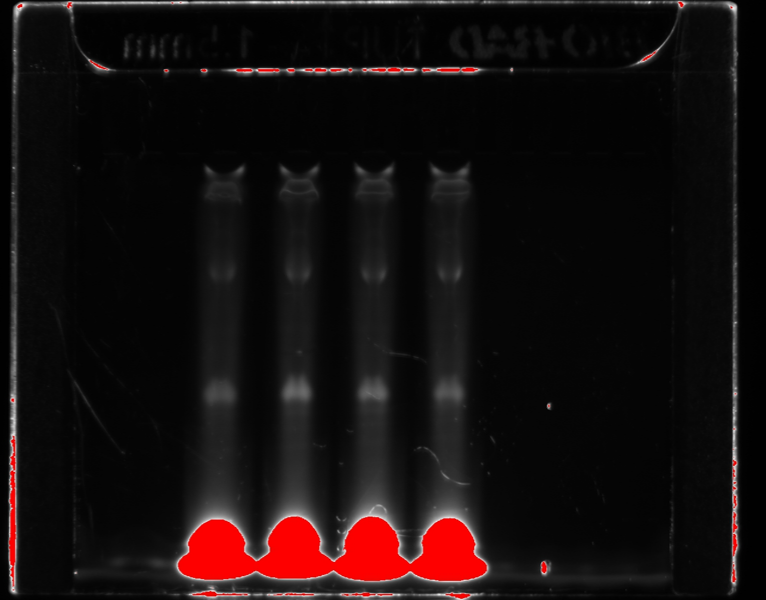

Supplement: Supplementary file 4 — Figure 2 raw data [file 44318_2025_532_MOESM4_ESM.zip › Figure 2/2B/SUMO1-Adenyl 10.1.23.tif]

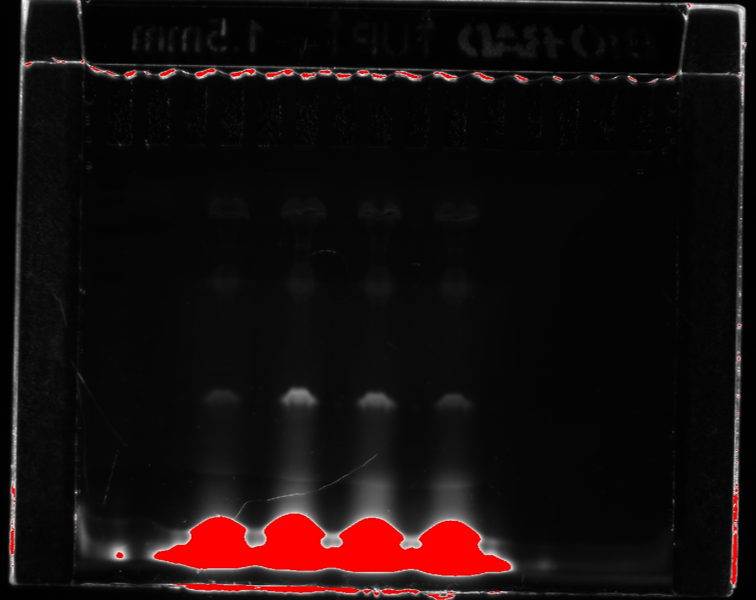

Supplement: Supplementary file 4 — Figure 2 raw data [file 44318_2025_532_MOESM4_ESM.zip › Figure 2/2B/SUMO1-Adenyl 6.1.23.tif]

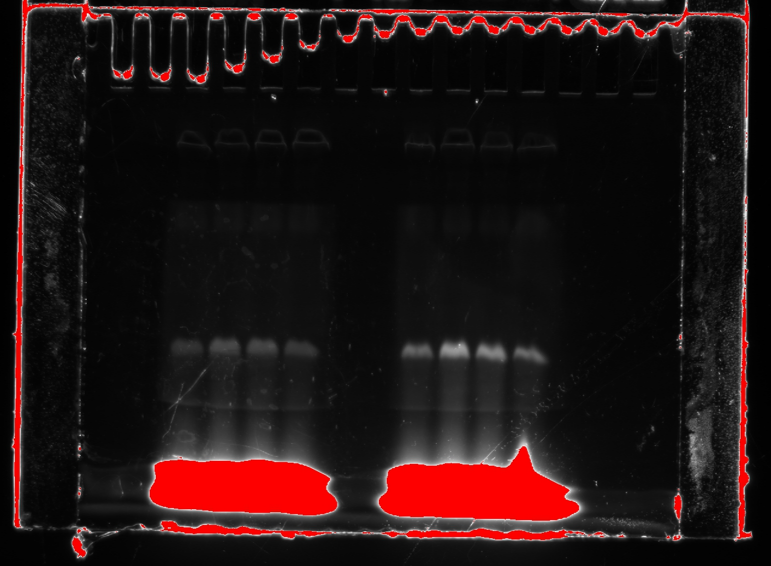

Supplement: Supplementary file 4 — Figure 2 raw data [file 44318_2025_532_MOESM4_ESM.zip › Figure 2/2B/SUMO1-Adenyl SUMO2-Adenyl 17.1.23.tif]

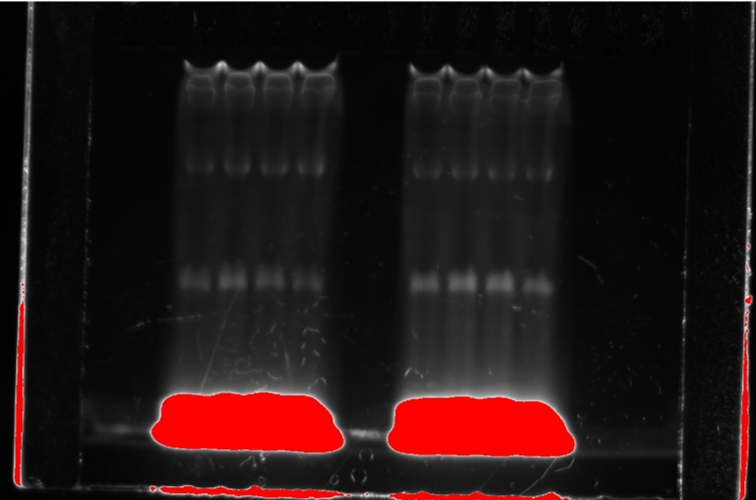

Supplement: Supplementary file 4 — Figure 2 raw data [file 44318_2025_532_MOESM4_ESM.zip › Figure 2/2B/SUMO1-Adenyl SUMO2-Adenyl 9.1.23.tif]

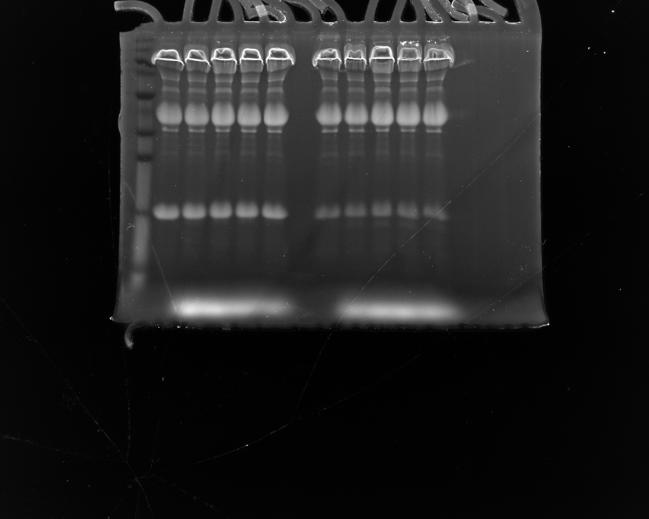

Supplement: Supplementary file 4 — Figure 2 raw data [file 44318_2025_532_MOESM4_ESM.zip › Figure 2/2B/SUMO1-Adenyl SUMO2-Adenyl sypro 17.1.23.tif]

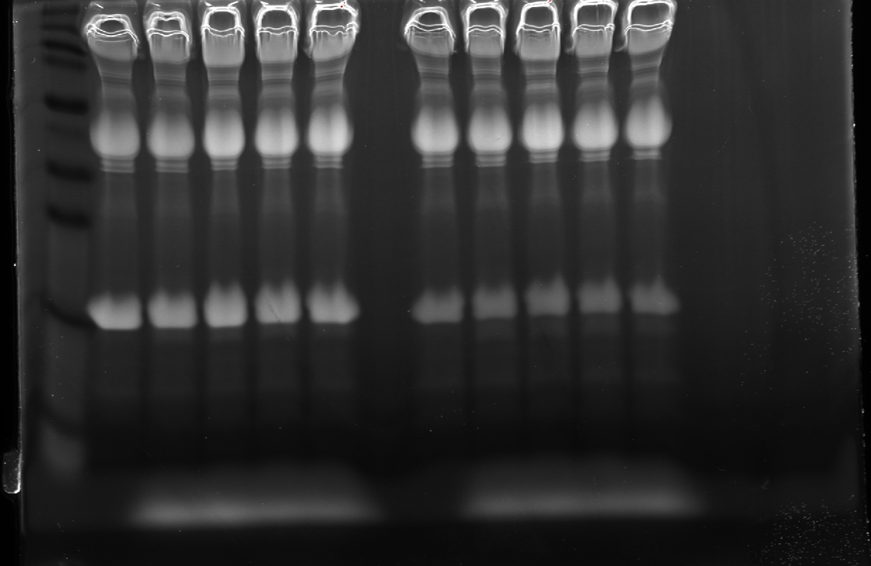

Supplement: Supplementary file 4 — Figure 2 raw data [file 44318_2025_532_MOESM4_ESM.zip › Figure 2/2B/SUMO1-Adenyl SUMO2-Adenyl sypro 9.1.23.tif]

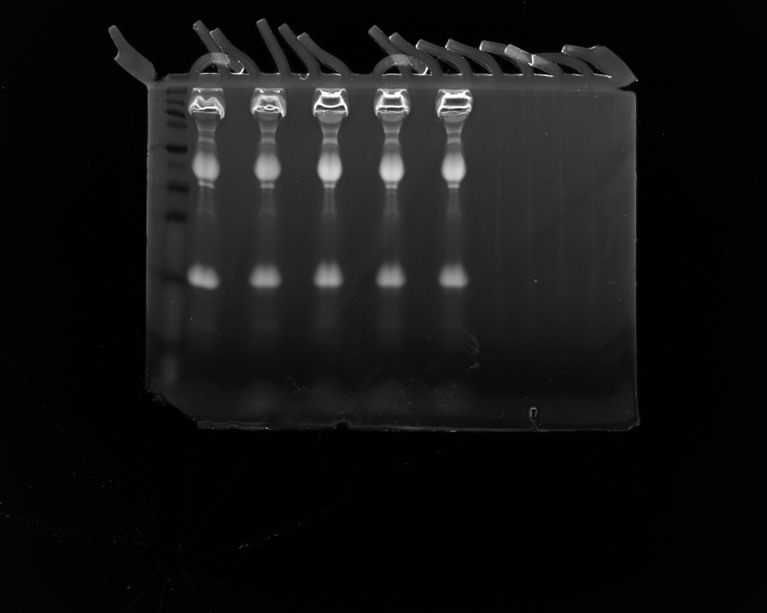

Supplement: Supplementary file 4 — Figure 2 raw data [file 44318_2025_532_MOESM4_ESM.zip › Figure 2/2B/SUMO1-Adenyl sypro 10.1.23.tif]

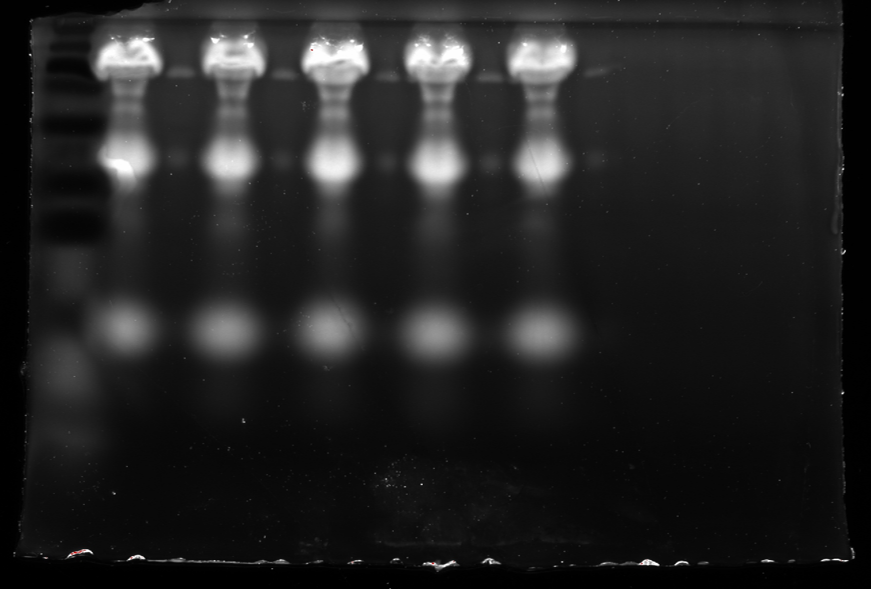

Supplement: Supplementary file 4 — Figure 2 raw data [file 44318_2025_532_MOESM4_ESM.zip › Figure 2/2B/SUMO1-Adenyl sypro 6.1.23.tif]

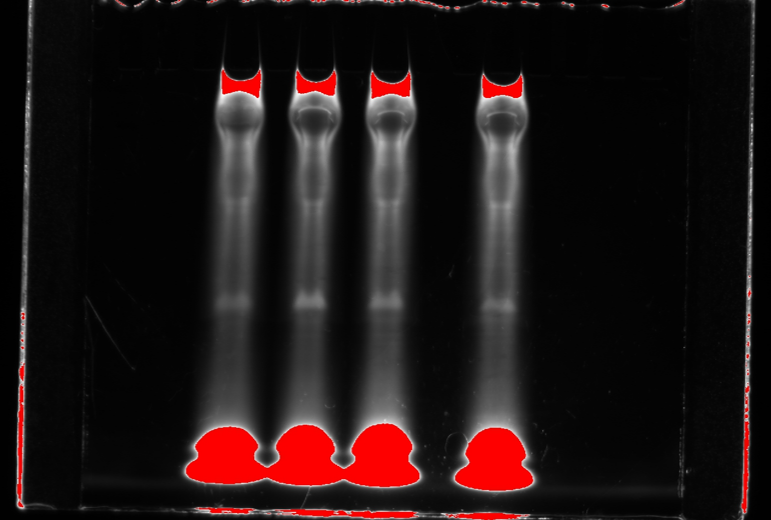

Supplement: Supplementary file 4 — Figure 2 raw data [file 44318_2025_532_MOESM4_ESM.zip › Figure 2/2B/SUMO2-Adenyl 16.1.23.tif]

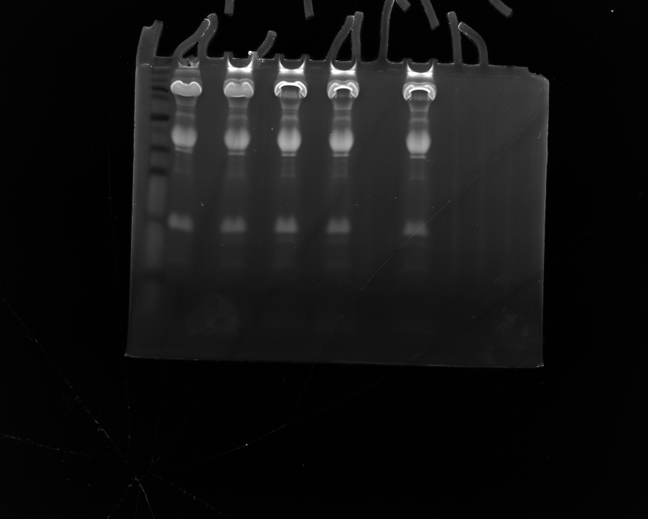

Supplement: Supplementary file 4 — Figure 2 raw data [file 44318_2025_532_MOESM4_ESM.zip › Figure 2/2B/SUMO2-Adenyl sypro 16.1.23.tif]

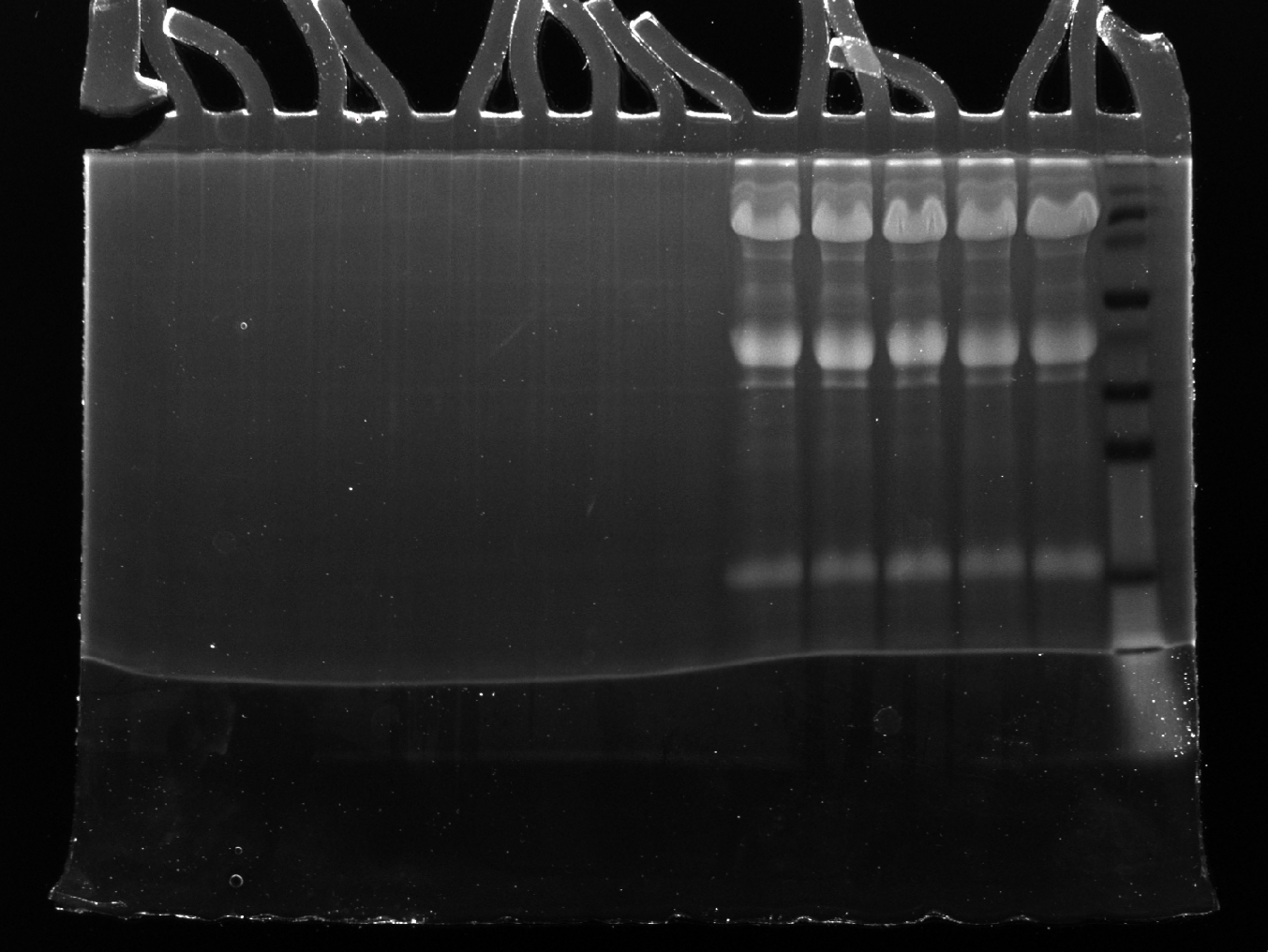

Supplement: Supplementary file 4 — Figure 2 raw data [file 44318_2025_532_MOESM4_ESM.zip › Figure 2/2B/SUMO2-Adenyl sypro.tif]

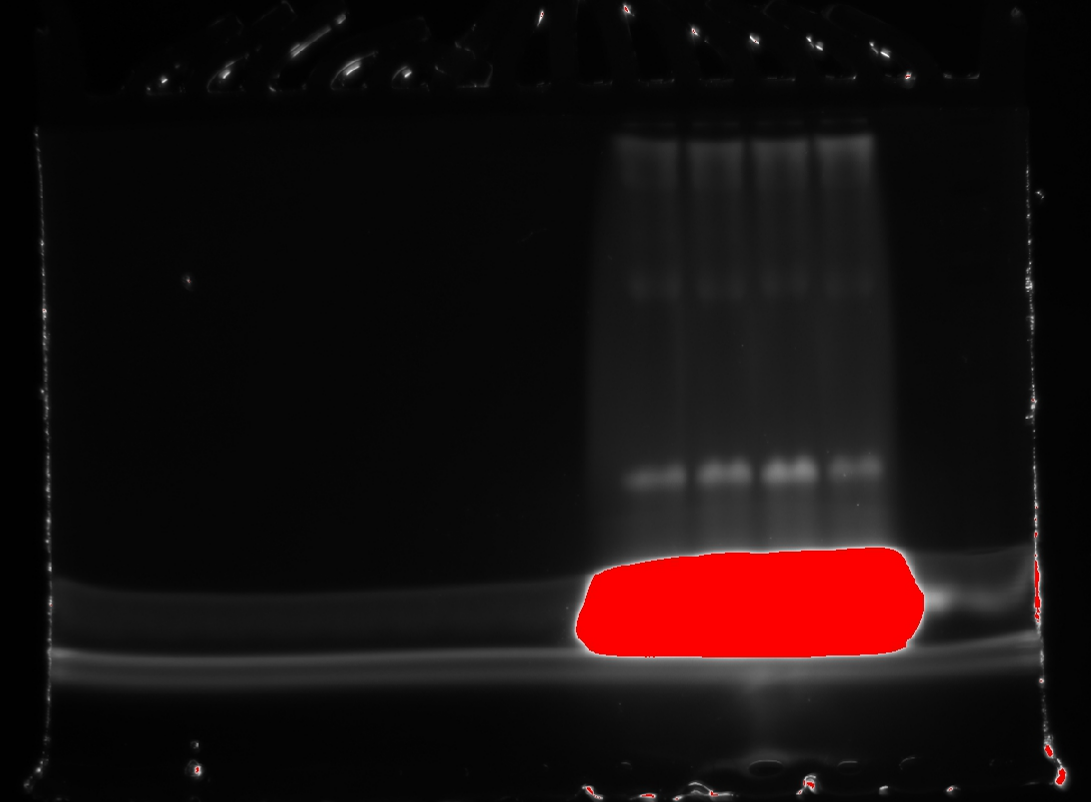

Supplement: Supplementary file 4 — Figure 2 raw data [file 44318_2025_532_MOESM4_ESM.zip › Figure 2/2B/SUMO2-Adenyl.tif]

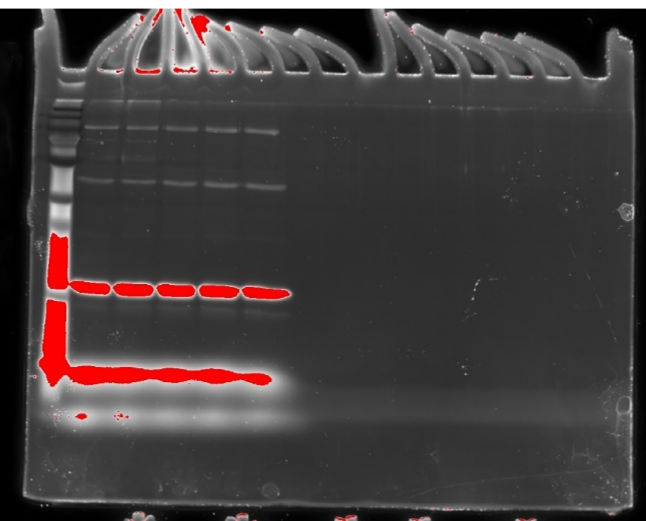

Supplement: Supplementary file 4 — Figure 2 raw data [file 44318_2025_532_MOESM4_ESM.zip › Figure 2/2C/N=1 Alexa488-SUMO1~SAE2 sypro.tif]

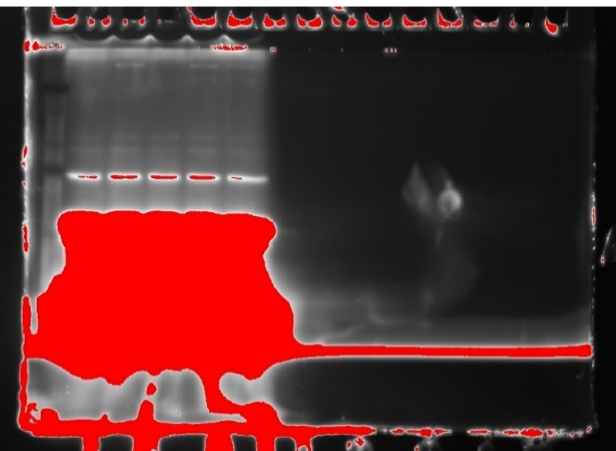

Supplement: Supplementary file 4 — Figure 2 raw data [file 44318_2025_532_MOESM4_ESM.zip › Figure 2/2C/N=1 Alexa488-SUMO1~SAE2.tif]

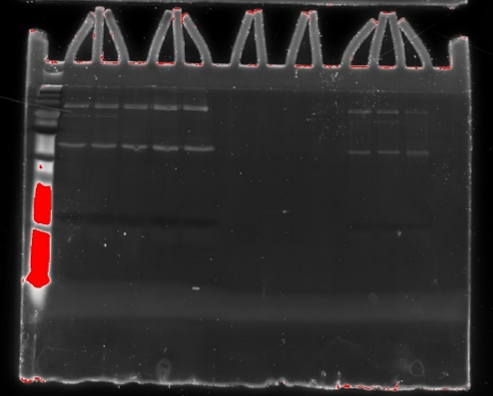

Supplement: Supplementary file 4 — Figure 2 raw data [file 44318_2025_532_MOESM4_ESM.zip › Figure 2/2C/N=1 Alexa647-SUMO2~SAE2 sypro.tif]

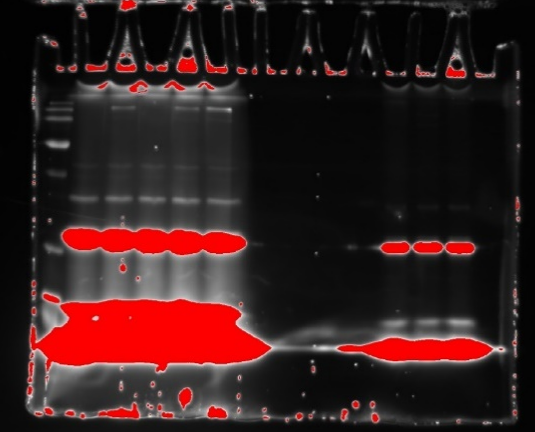

Supplement: Supplementary file 4 — Figure 2 raw data [file 44318_2025_532_MOESM4_ESM.zip › Figure 2/2C/N=1 Alexa647-SUMO2~SAE2.tif]

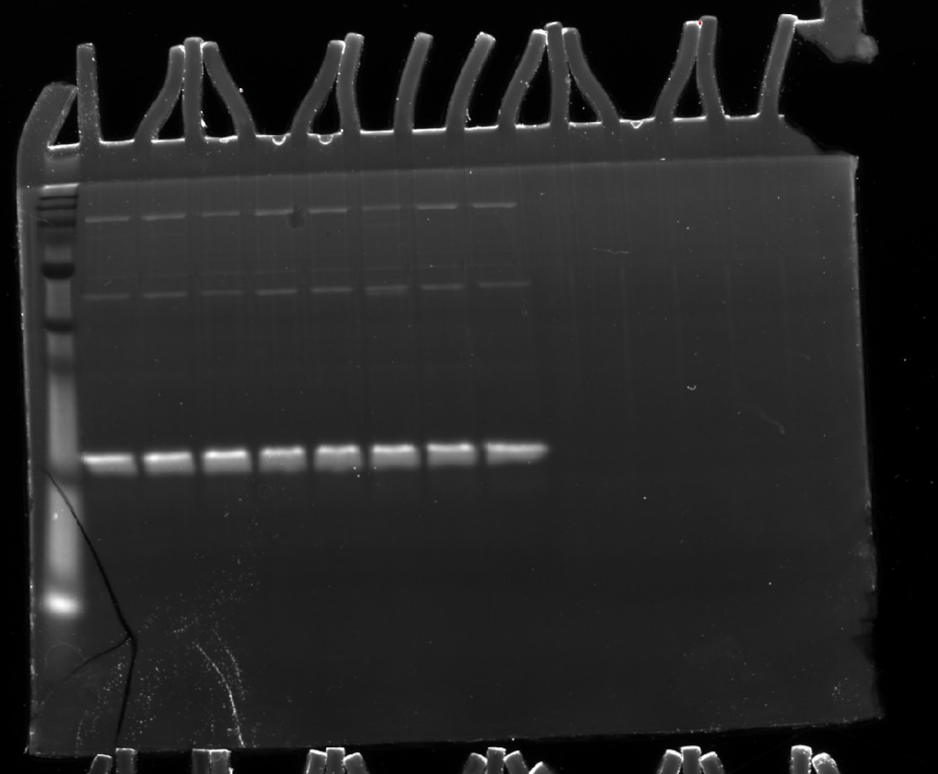

Supplement: Supplementary file 4 — Figure 2 raw data [file 44318_2025_532_MOESM4_ESM.zip › Figure 2/2C/N=2 Alexa488-SUMO1~SAE2 sypro.tif]

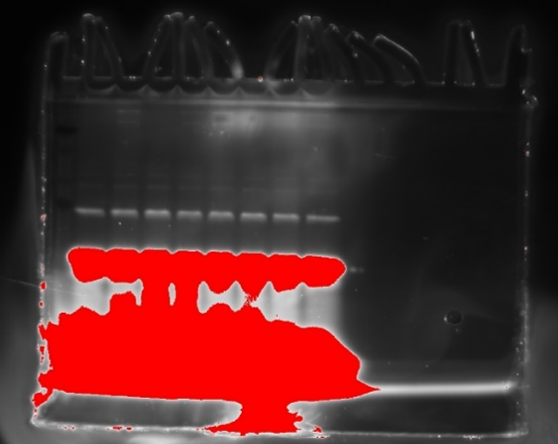

Supplement: Supplementary file 4 — Figure 2 raw data [file 44318_2025_532_MOESM4_ESM.zip › Figure 2/2C/N=2 Alexa488-SUMO1~SAE2.tif]

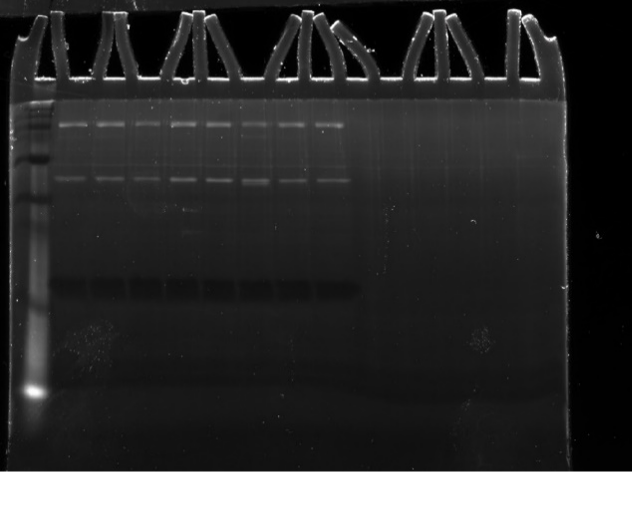

Supplement: Supplementary file 4 — Figure 2 raw data [file 44318_2025_532_MOESM4_ESM.zip › Figure 2/2C/N=2 Alexa647-SUMO2~SAE2 sypro.tif]

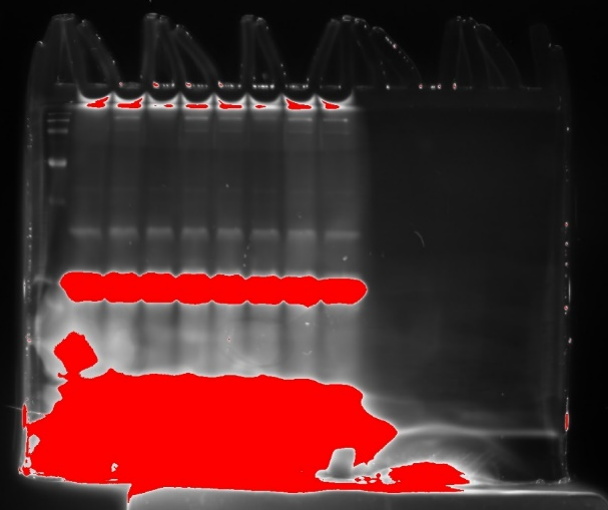

Supplement: Supplementary file 4 — Figure 2 raw data [file 44318_2025_532_MOESM4_ESM.zip › Figure 2/2C/N=2 Alexa647-SUMO2~SAE2.tif]

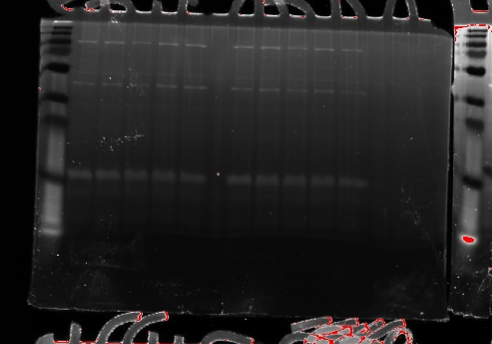

Supplement: Supplementary file 4 — Figure 2 raw data [file 44318_2025_532_MOESM4_ESM.zip › Figure 2/2C/N=3 Alexa488-SUMO1~SAE2 sypro.tif]

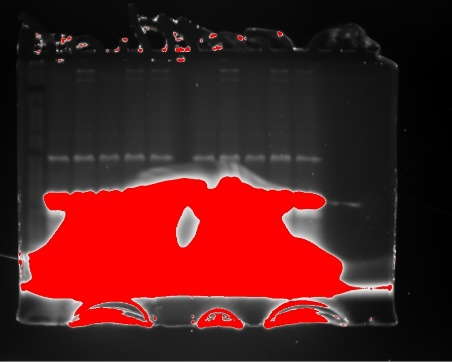

Supplement: Supplementary file 4 — Figure 2 raw data [file 44318_2025_532_MOESM4_ESM.zip › Figure 2/2C/N=3 Alexa488-SUMO1~SAE2.tif]

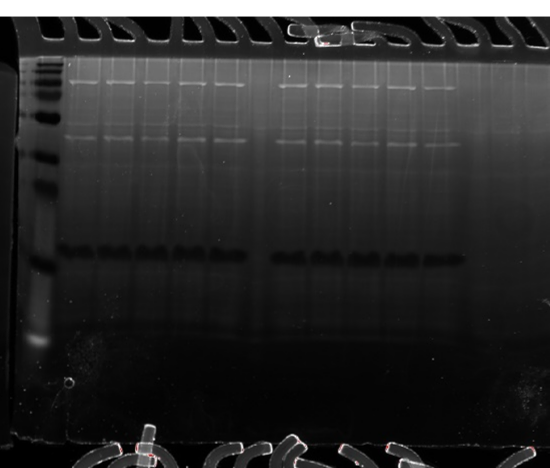

Supplement: Supplementary file 4 — Figure 2 raw data [file 44318_2025_532_MOESM4_ESM.zip › Figure 2/2C/N=3 Alexa647-SUMO2~SAE2 sypro.tif]

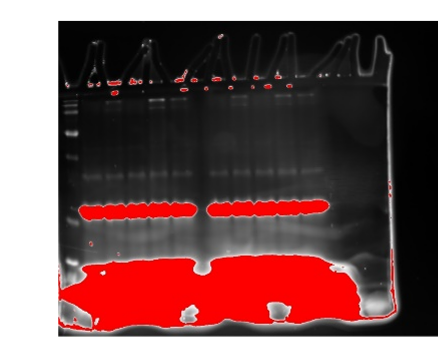

Supplement: Supplementary file 4 — Figure 2 raw data [file 44318_2025_532_MOESM4_ESM.zip › Figure 2/2C/N=3 Alexa647-SUMO2~SAE2.tif]

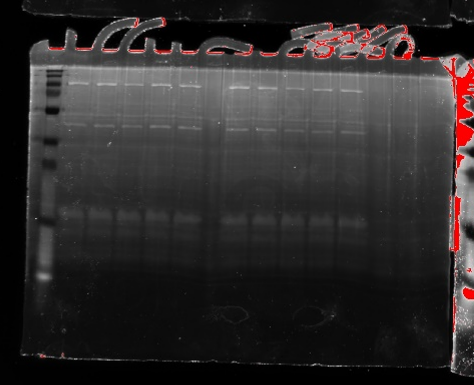

Supplement: Supplementary file 4 — Figure 2 raw data [file 44318_2025_532_MOESM4_ESM.zip › Figure 2/2C/N=4 Alexa488-SUMO1~SAE2 sypro.tif]

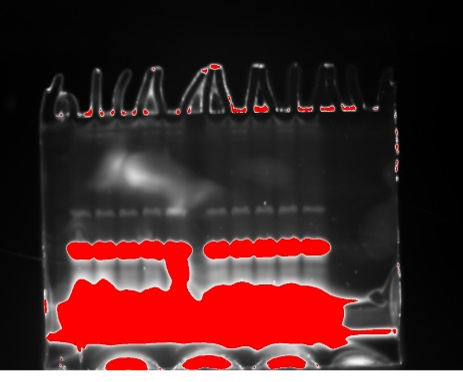

Supplement: Supplementary file 4 — Figure 2 raw data [file 44318_2025_532_MOESM4_ESM.zip › Figure 2/2C/N=4 Alexa488-SUMO1~SAE2.tif]

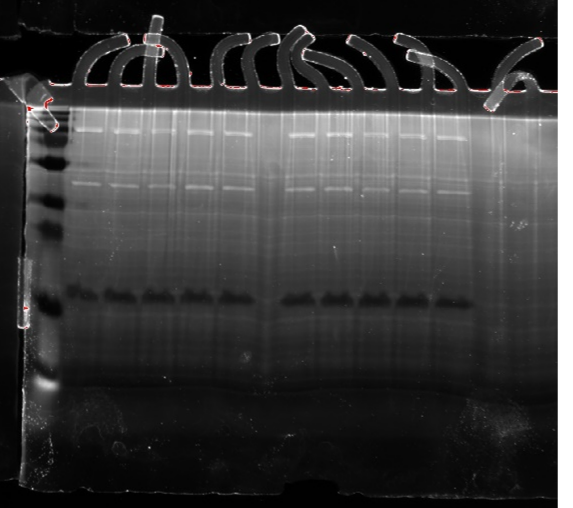

Supplement: Supplementary file 4 — Figure 2 raw data [file 44318_2025_532_MOESM4_ESM.zip › Figure 2/2C/N=4 Alexa647-SUMO2~SAE2 sypro.tif]

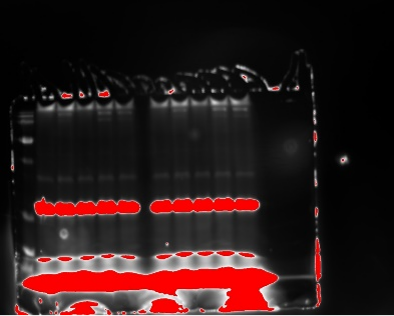

Supplement: Supplementary file 4 — Figure 2 raw data [file 44318_2025_532_MOESM4_ESM.zip › Figure 2/2C/N=4 Alexa647-SUMO2~SAE2.tif]

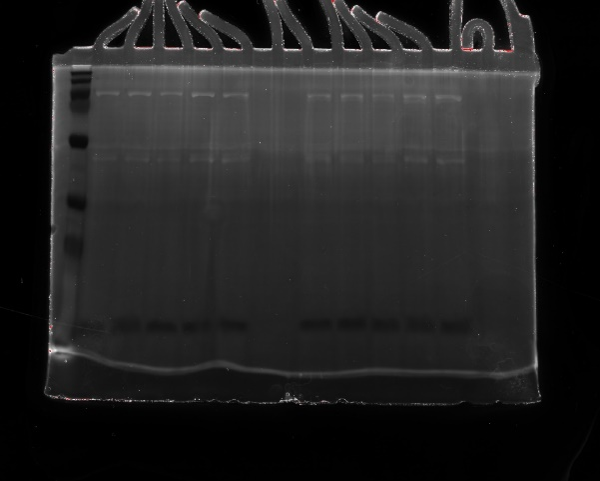

Supplement: Supplementary file 4 — Figure 2 raw data [file 44318_2025_532_MOESM4_ESM.zip › Figure 2/2C/N=5 Alexa647-SUMO2~SAE2 sypro.tif]

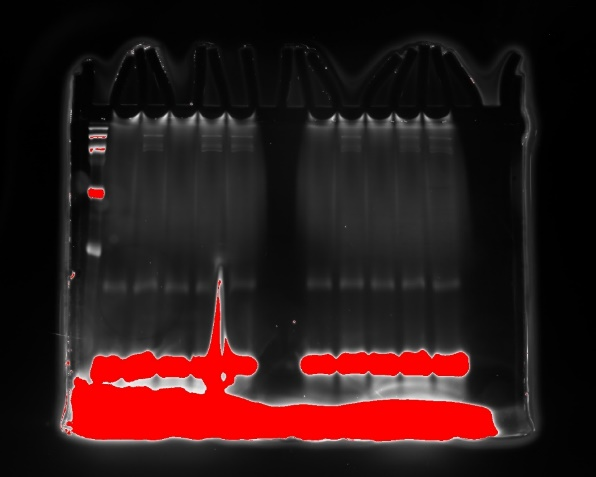

Supplement: Supplementary file 4 — Figure 2 raw data [file 44318_2025_532_MOESM4_ESM.zip › Figure 2/2C/N=5 Alexa647-SUMO2~SAE2.tif]

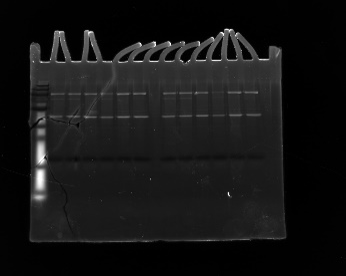

Supplement: Supplementary file 4 — Figure 2 raw data [file 44318_2025_532_MOESM4_ESM.zip › Figure 2/2C/Test Alexa647-SUMO2~SAE2 sypro.tif]

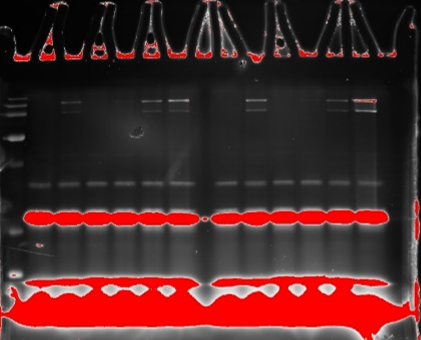

Supplement: Supplementary file 4 — Figure 2 raw data [file 44318_2025_532_MOESM4_ESM.zip › Figure 2/2C/Test Alexa647-SUMO2~SAE2.tif]

## Slide 1
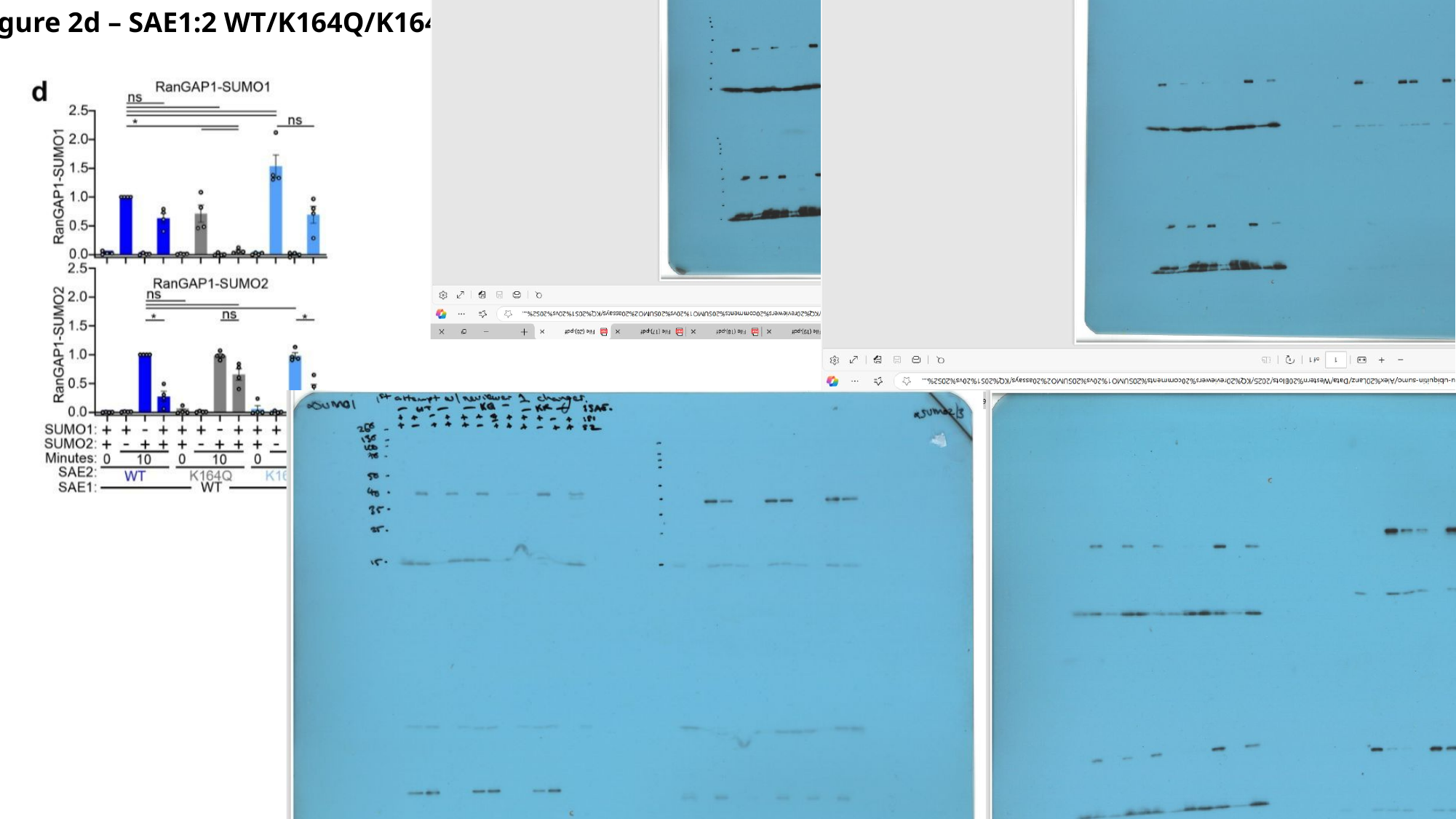

Figure 2d – SAE1:2 WT/K164Q/K164R RanGAP1 SUMO1/SUMO2ylation assay.

Supplement: Supplementary file 4 — Figure 2 raw data [file 44318_2025_532_MOESM4_ESM.zip › Figure 2/2D/Figure 2d.pptx]

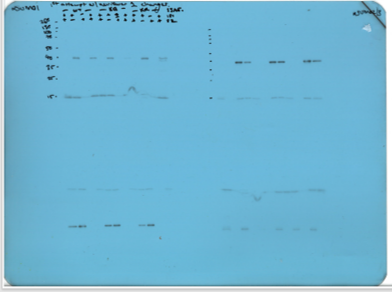

Supplement: Supplementary file 4 — Figure 2 raw data [file 44318_2025_532_MOESM4_ESM.zip › Figure 2/2D/N=1 WT-KQ-KQ S1 vs S2 RanGAP1 SUMOylation.tif]

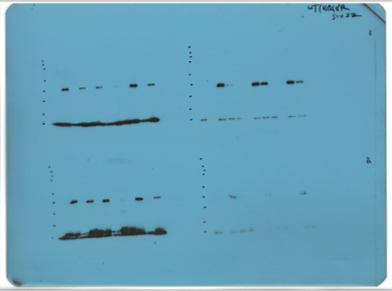

Supplement: Supplementary file 4 — Figure 2 raw data [file 44318_2025_532_MOESM4_ESM.zip › Figure 2/2D/N=2 WT-KQ-KQ S1 vs S2 RanGAP1 SUMOylation.tif]

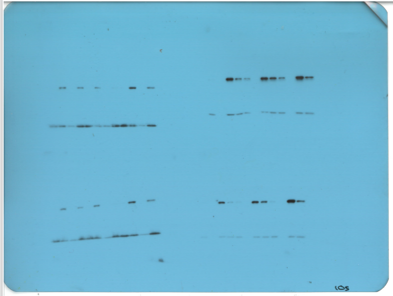

Supplement: Supplementary file 4 — Figure 2 raw data [file 44318_2025_532_MOESM4_ESM.zip › Figure 2/2D/N=3,4 WT-KQ-KQ S1 vs S2 RanGAP1 SUMOylation.tif]

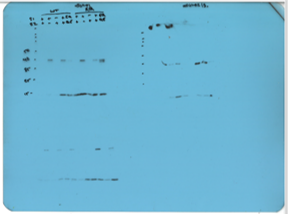

Supplement: Supplementary file 4 — Figure 2 raw data [file 44318_2025_532_MOESM4_ESM.zip › Figure 2/2F/N=1 WT-KQ S1-EQ vs S2-QE RanGAP1 SUMOylation.tif]

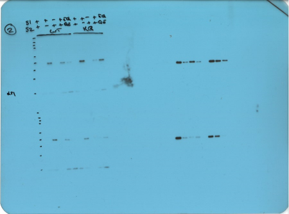

Supplement: Supplementary file 4 — Figure 2 raw data [file 44318_2025_532_MOESM4_ESM.zip › Figure 2/2F/N=2 WT-KQ S1-EQ vs S2-QE RanGAP1 SUMOylation.tif]

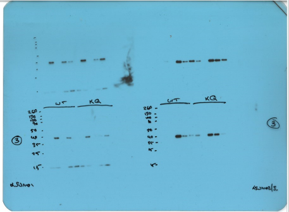

Supplement: Supplementary file 4 — Figure 2 raw data [file 44318_2025_532_MOESM4_ESM.zip › Figure 2/2F/N=3 WT-KQ S1-EQ vs S2-QE RanGAP1 SUMOylation.tif]

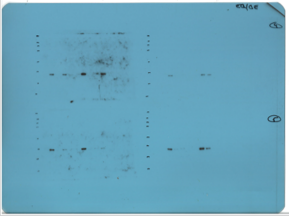

Supplement: Supplementary file 4 — Figure 2 raw data [file 44318_2025_532_MOESM4_ESM.zip › Figure 2/2F/N=4,5 WT-KQ S1-EQ vs S2-QE RanGAP1 SUMOylation SUMO1 blots.tif]

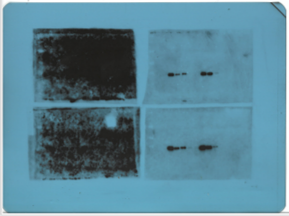

Supplement: Supplementary file 4 — Figure 2 raw data [file 44318_2025_532_MOESM4_ESM.zip › Figure 2/2F/N=4,5 WT-KQ S1-EQ vs S2-QE RanGAP1 SUMOylation SUMO2 blots.tif]

## Slide 1
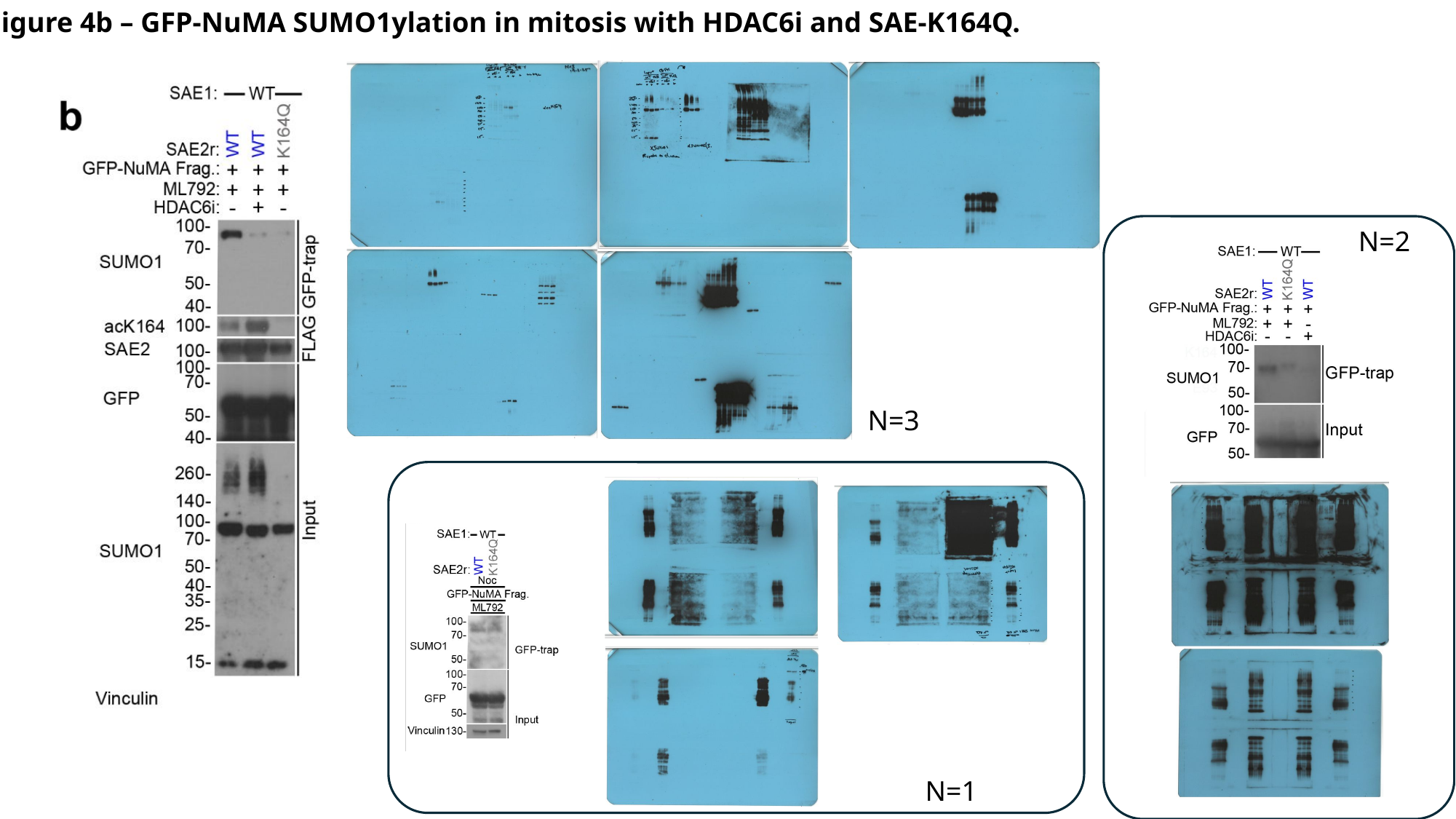

Figure 4b – GFP-NuMA SUMO1ylation in mitosis with HDAC6i and SAE-K164Q.
N=2
N=3
N=1

Supplement: Supplementary file 5 — Figure 4 raw data [file 44318_2025_532_MOESM5_ESM.zip › Figure 4/4A/Figure 4a.pptx]

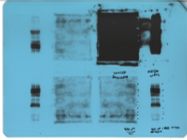

Supplement: Supplementary file 5 — Figure 4 raw data [file 44318_2025_532_MOESM5_ESM.zip › Figure 4/4A/N=1 GFP-NuMA-SUMO1 2.tif]

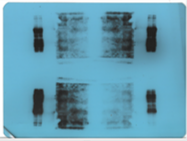

Supplement: Supplementary file 5 — Figure 4 raw data [file 44318_2025_532_MOESM5_ESM.zip › Figure 4/4A/N=1 GFP-NuMA-SUMO1 3.tif]

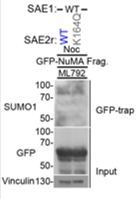

Supplement: Supplementary file 5 — Figure 4 raw data [file 44318_2025_532_MOESM5_ESM.zip › Figure 4/4A/N=1 GFP-NuMA-SUMO1 composite.tif]

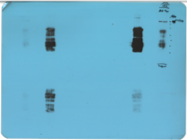

Supplement: Supplementary file 5 — Figure 4 raw data [file 44318_2025_532_MOESM5_ESM.zip › Figure 4/4A/N=1 GFP-NuMA-SUMO1.tif]

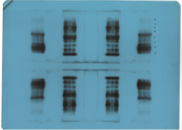

Supplement: Supplementary file 5 — Figure 4 raw data [file 44318_2025_532_MOESM5_ESM.zip › Figure 4/4A/N=2 GFP-NuMA-SUMO1 2.tif]

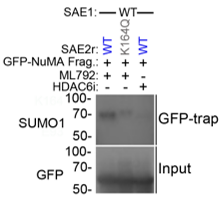

Supplement: Supplementary file 5 — Figure 4 raw data [file 44318_2025_532_MOESM5_ESM.zip › Figure 4/4A/N=2 GFP-NuMA-SUMO1 composite.tif]

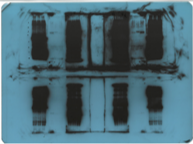

Supplement: Supplementary file 5 — Figure 4 raw data [file 44318_2025_532_MOESM5_ESM.zip › Figure 4/4A/N=2 GFP-NuMA-SUMO1.tif]

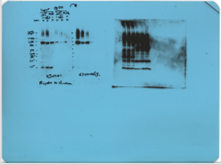

Supplement: Supplementary file 5 — Figure 4 raw data [file 44318_2025_532_MOESM5_ESM.zip › Figure 4/4A/N=3 GFP-NuMA-SUMO1 2.tif]

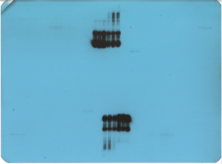

Supplement: Supplementary file 5 — Figure 4 raw data [file 44318_2025_532_MOESM5_ESM.zip › Figure 4/4A/N=3 GFP-NuMA-SUMO1 3.tif]

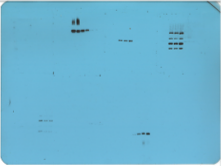

Supplement: Supplementary file 5 — Figure 4 raw data [file 44318_2025_532_MOESM5_ESM.zip › Figure 4/4A/N=3 GFP-NuMA-SUMO1 4.tif]

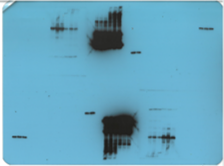

Supplement: Supplementary file 5 — Figure 4 raw data [file 44318_2025_532_MOESM5_ESM.zip › Figure 4/4A/N=3 GFP-NuMA-SUMO1 5.tif]

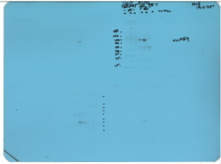

Supplement: Supplementary file 5 — Figure 4 raw data [file 44318_2025_532_MOESM5_ESM.zip › Figure 4/4A/N=3 GFP-NuMA-SUMO1.tif]

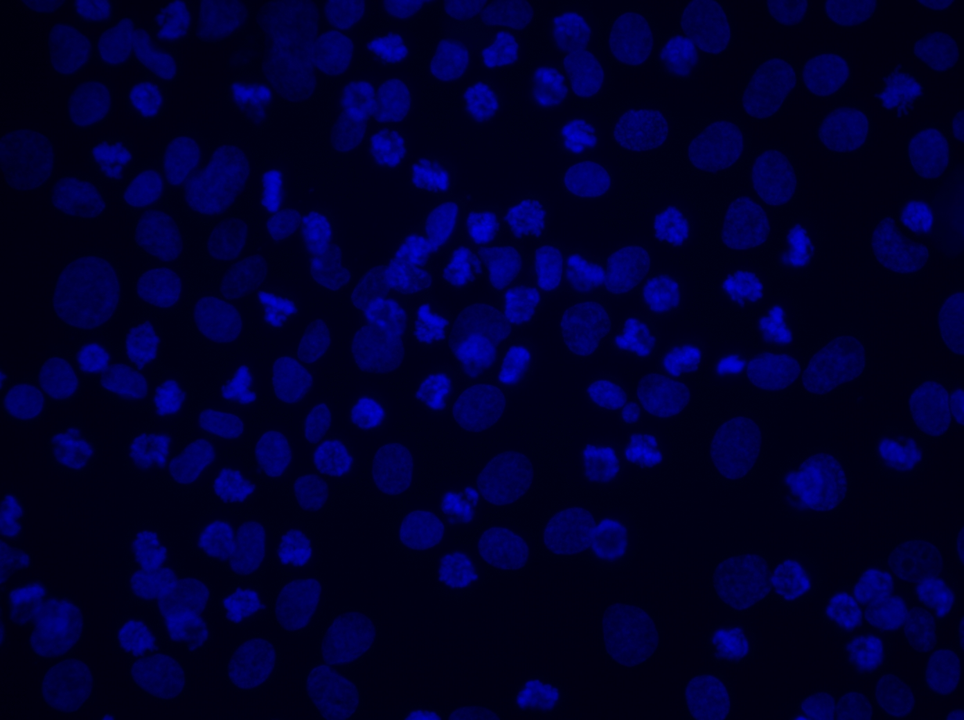

Supplement: Supplementary file 5 — Figure 4 raw data [file 44318_2025_532_MOESM5_ESM.zip › Figure 4/4B/2 and 3 NuMA structures example.tif]

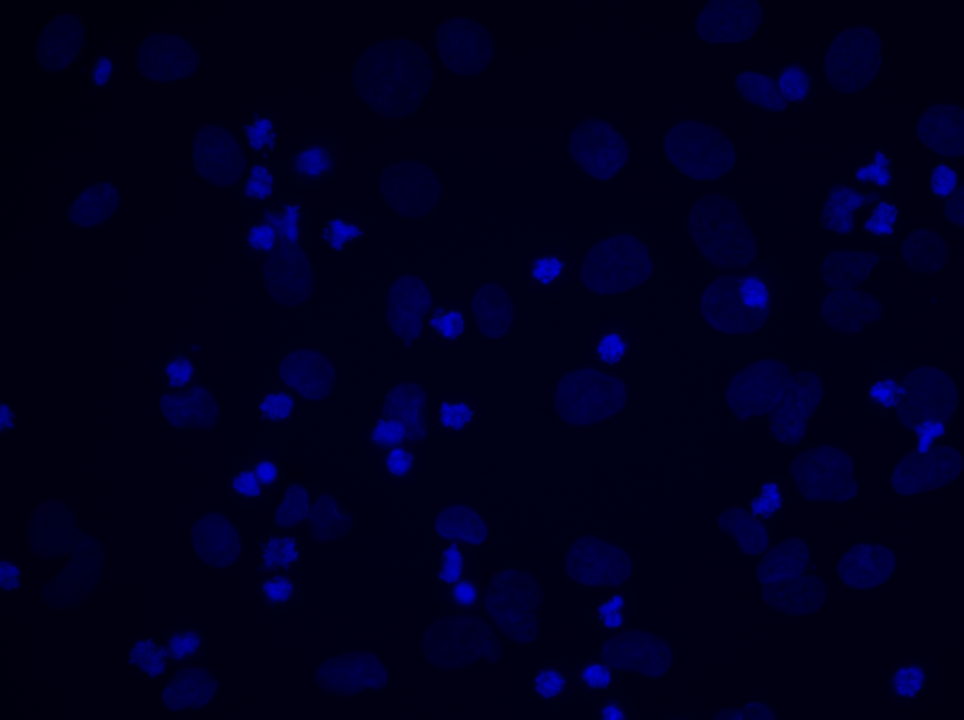

Supplement: Supplementary file 5 — Figure 4 raw data [file 44318_2025_532_MOESM5_ESM.zip › Figure 4/4B/4 NuMA structures example.tif]

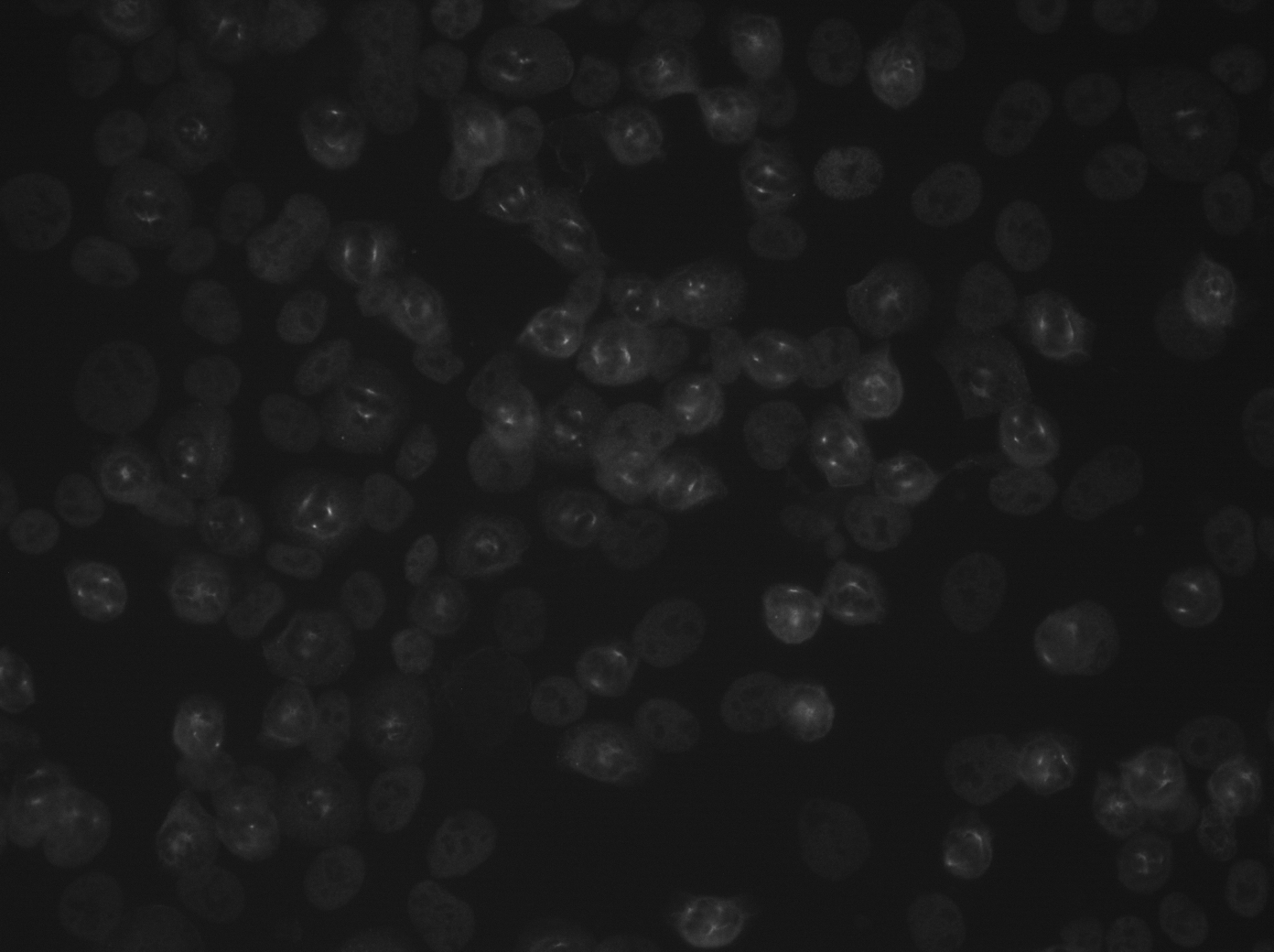

Supplement: Supplementary file 5 — Figure 4 raw data [file 44318_2025_532_MOESM5_ESM.zip › Figure 4/4B/N=2 NuMA red structures KQ or HDAC6i GFP-NuMA 5.8.24_-_Image067_ch02.tif]

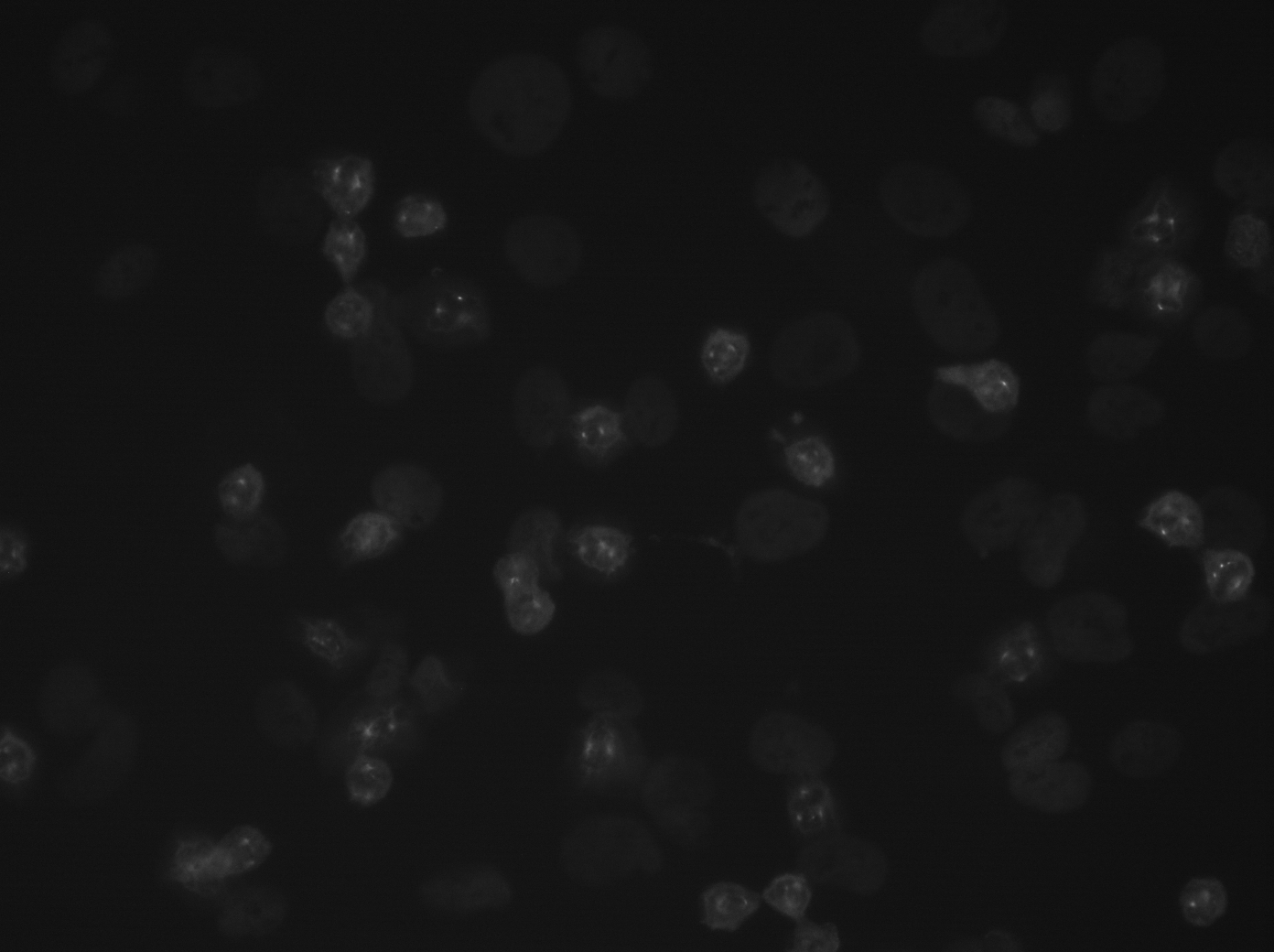

Supplement: Supplementary file 5 — Figure 4 raw data [file 44318_2025_532_MOESM5_ESM.zip › Figure 4/4B/N=2 NuMA red structures KQ or HDAC6i GFP-NuMA 5.8.24_KQ_Image123_ch02.tif]

## Slide 1
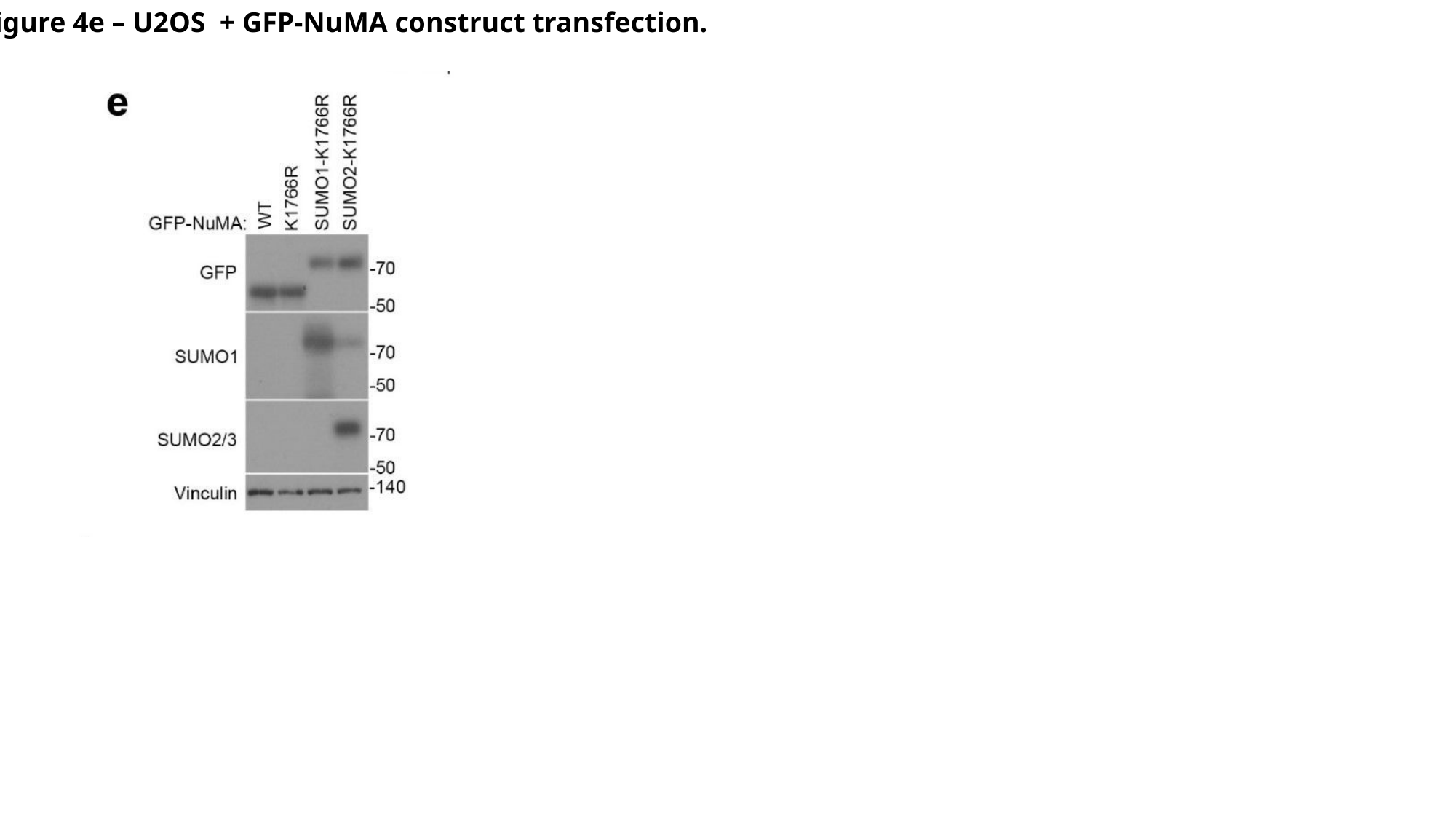

Figure 4e – U2OS + GFP-NuMA construct transfection.

Supplement: Supplementary file 5 — Figure 4 raw data [file 44318_2025_532_MOESM5_ESM.zip › Figure 4/4E/Figure 4e.pptx]

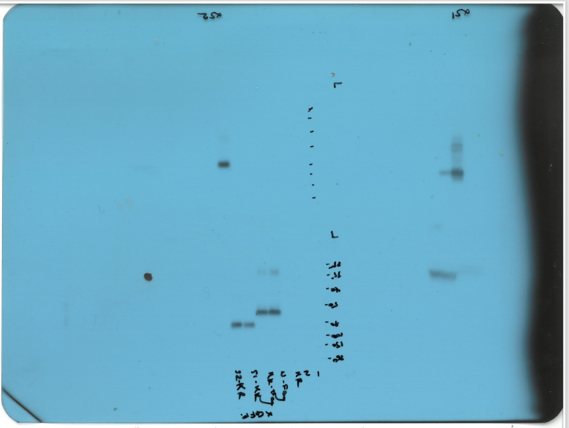

Supplement: Supplementary file 5 — Figure 4 raw data [file 44318_2025_532_MOESM5_ESM.zip › Figure 4/4E/GFP-NuMA construct transfectios aGFP, aSUMO1, aSUMO2 westerns.tif]

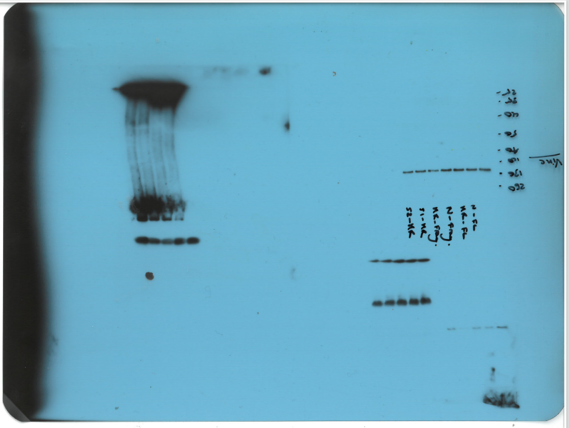

Supplement: Supplementary file 5 — Figure 4 raw data [file 44318_2025_532_MOESM5_ESM.zip › Figure 4/4E/GFP-NuMA construct transfectios aVinculin westerns.tif]

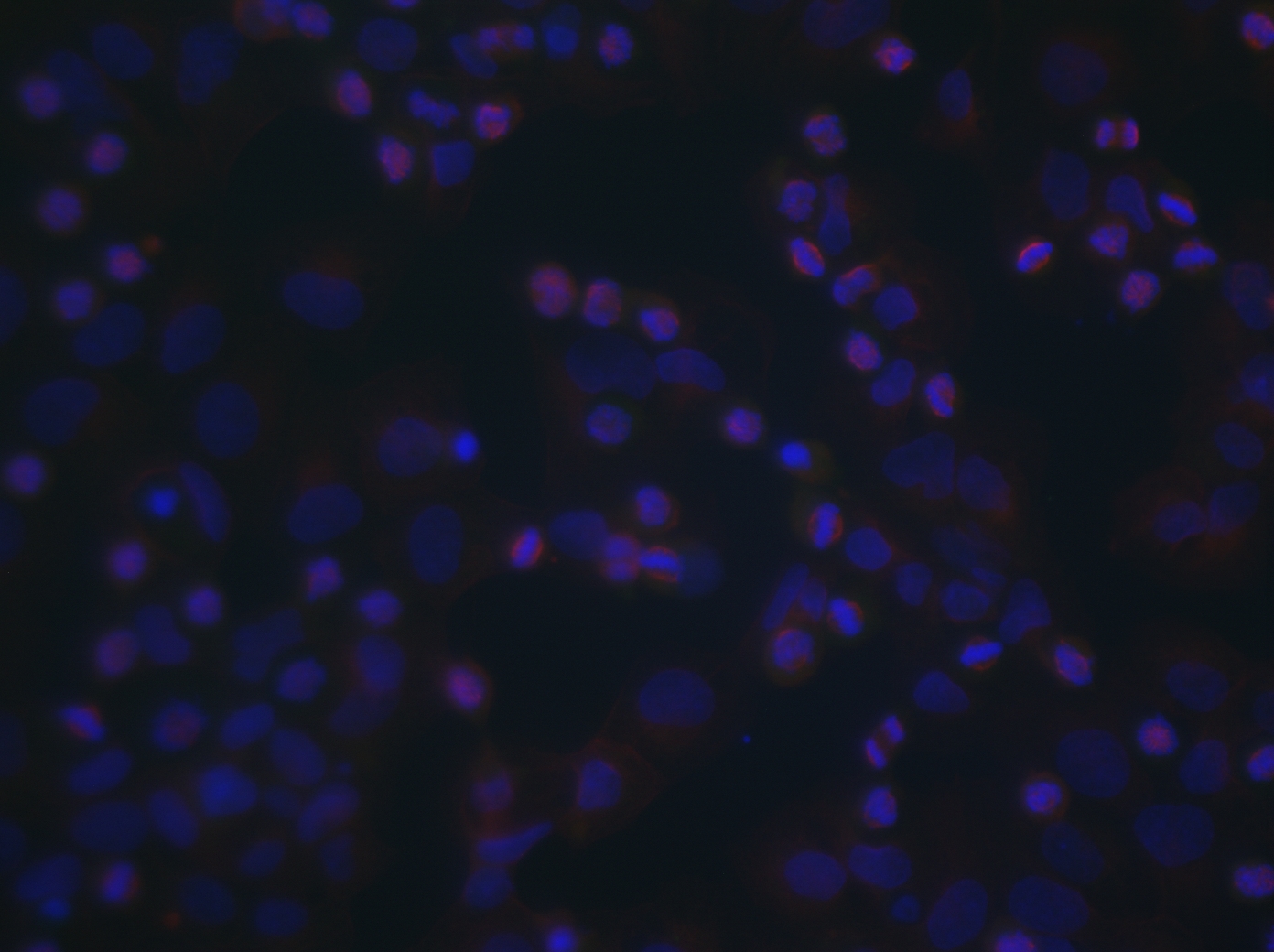

Supplement: Supplementary file 5 — Figure 4 raw data [file 44318_2025_532_MOESM5_ESM.zip › Figure 4/4F/-_Image068.jpg]

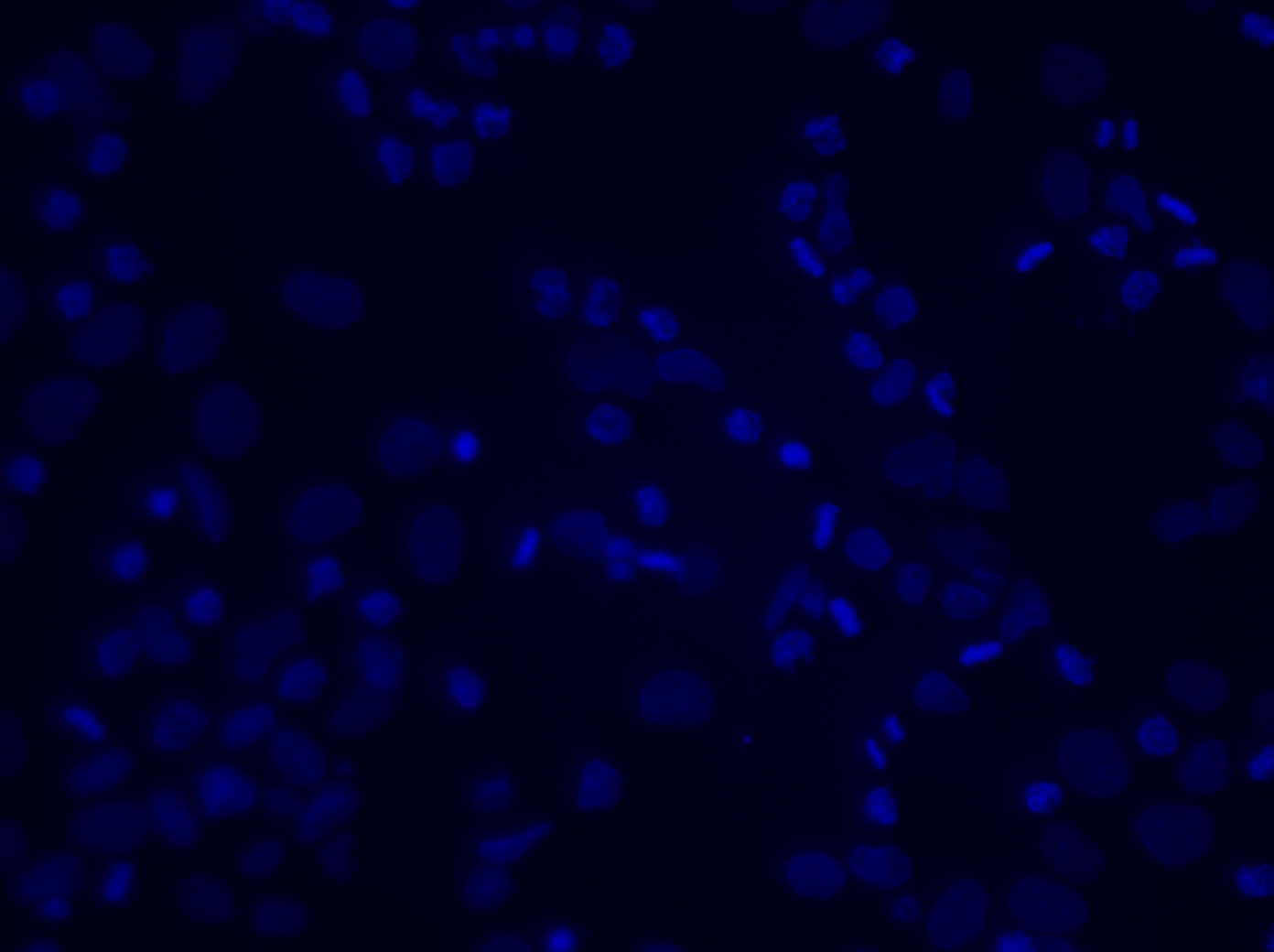

Supplement: Supplementary file 5 — Figure 4 raw data [file 44318_2025_532_MOESM5_ESM.zip › Figure 4/4F/-_Image068_ch00.jpg]

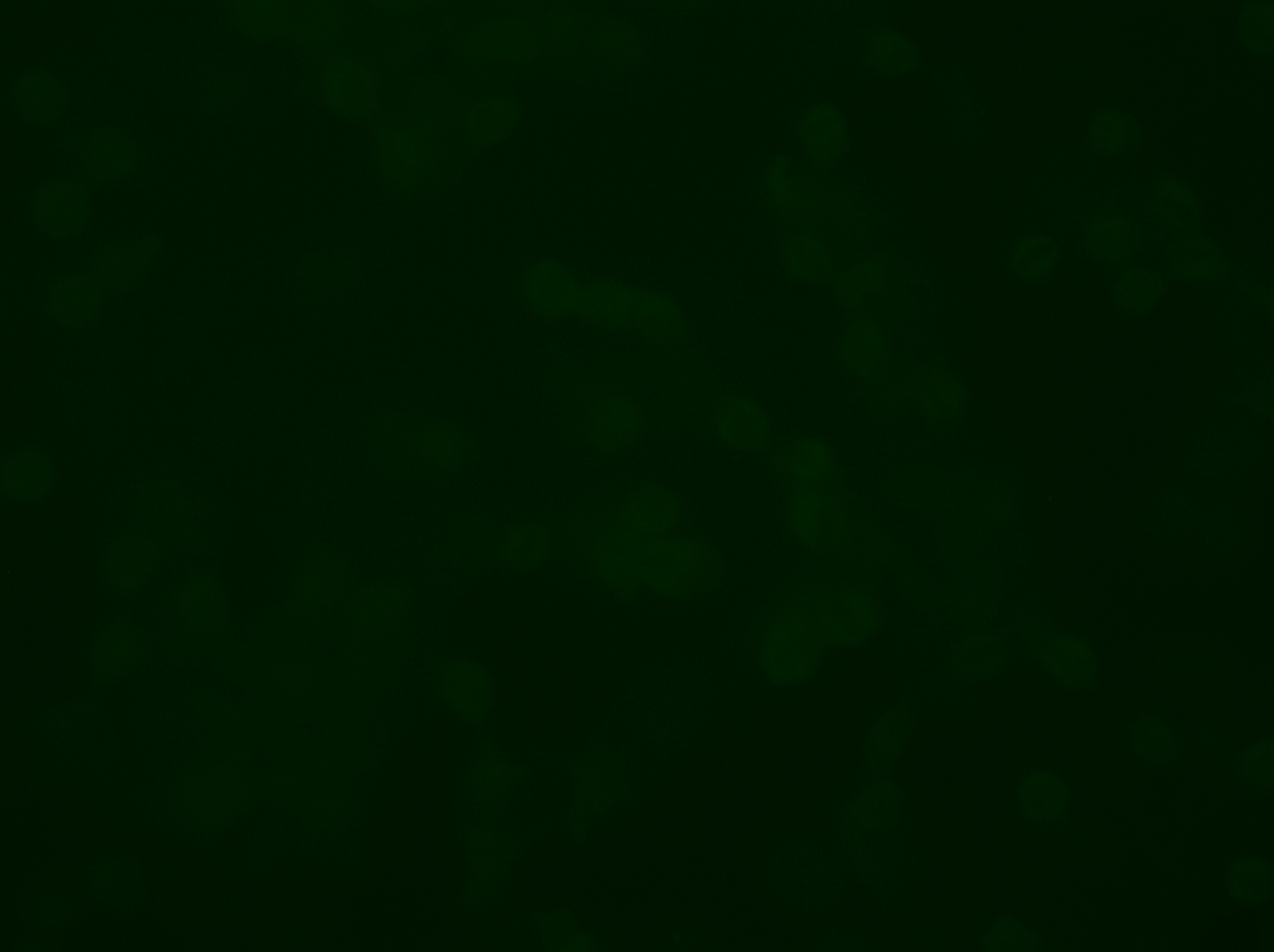

Supplement: Supplementary file 5 — Figure 4 raw data [file 44318_2025_532_MOESM5_ESM.zip › Figure 4/4F/-_Image068_ch01.jpg]

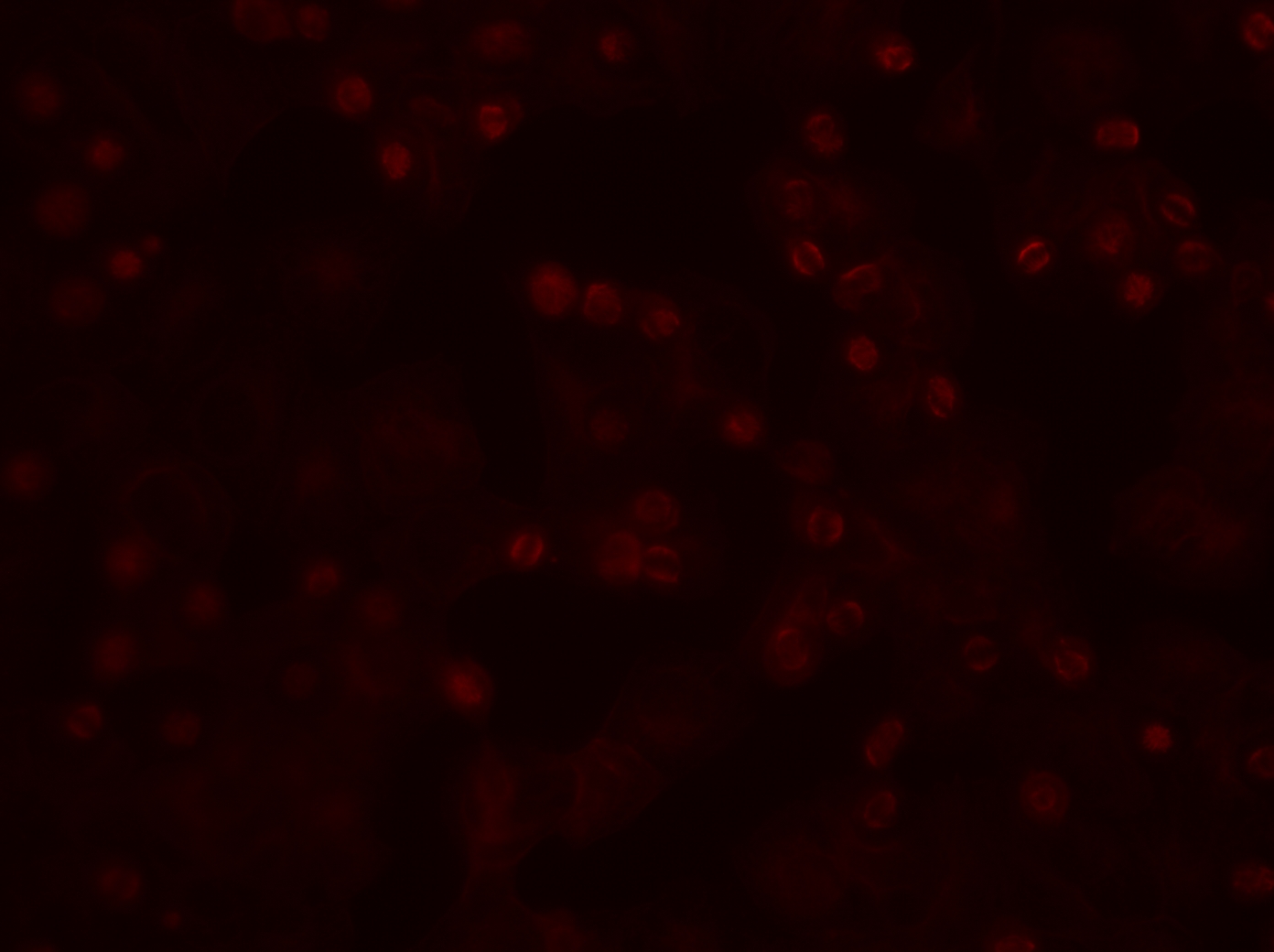

Supplement: Supplementary file 5 — Figure 4 raw data [file 44318_2025_532_MOESM5_ESM.zip › Figure 4/4F/-_Image068_ch02.jpg]

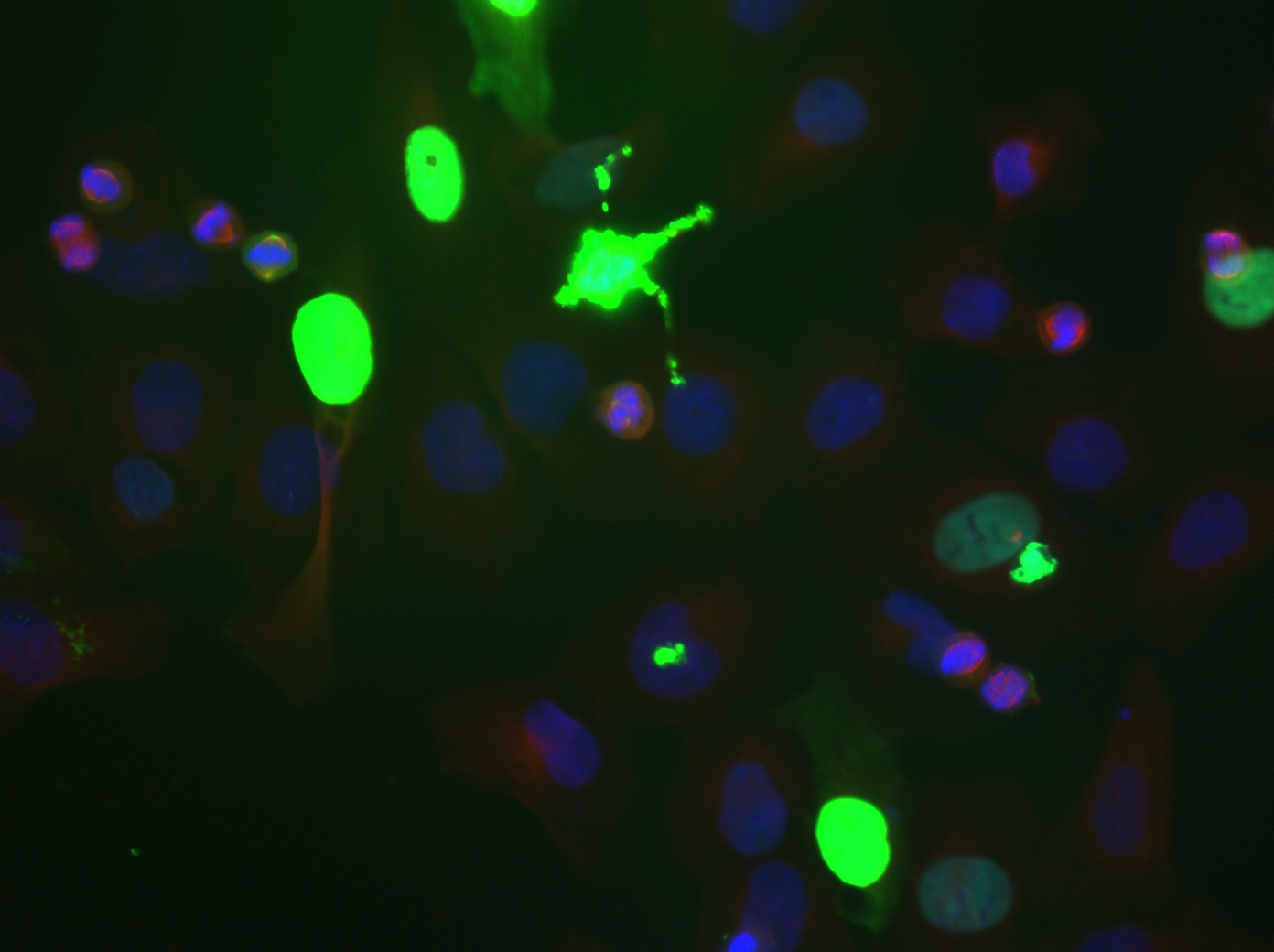

Supplement: Supplementary file 5 — Figure 4 raw data [file 44318_2025_532_MOESM5_ESM.zip › Figure 4/4F/KQ GFP-NuMA-KR_Image025.jpg]

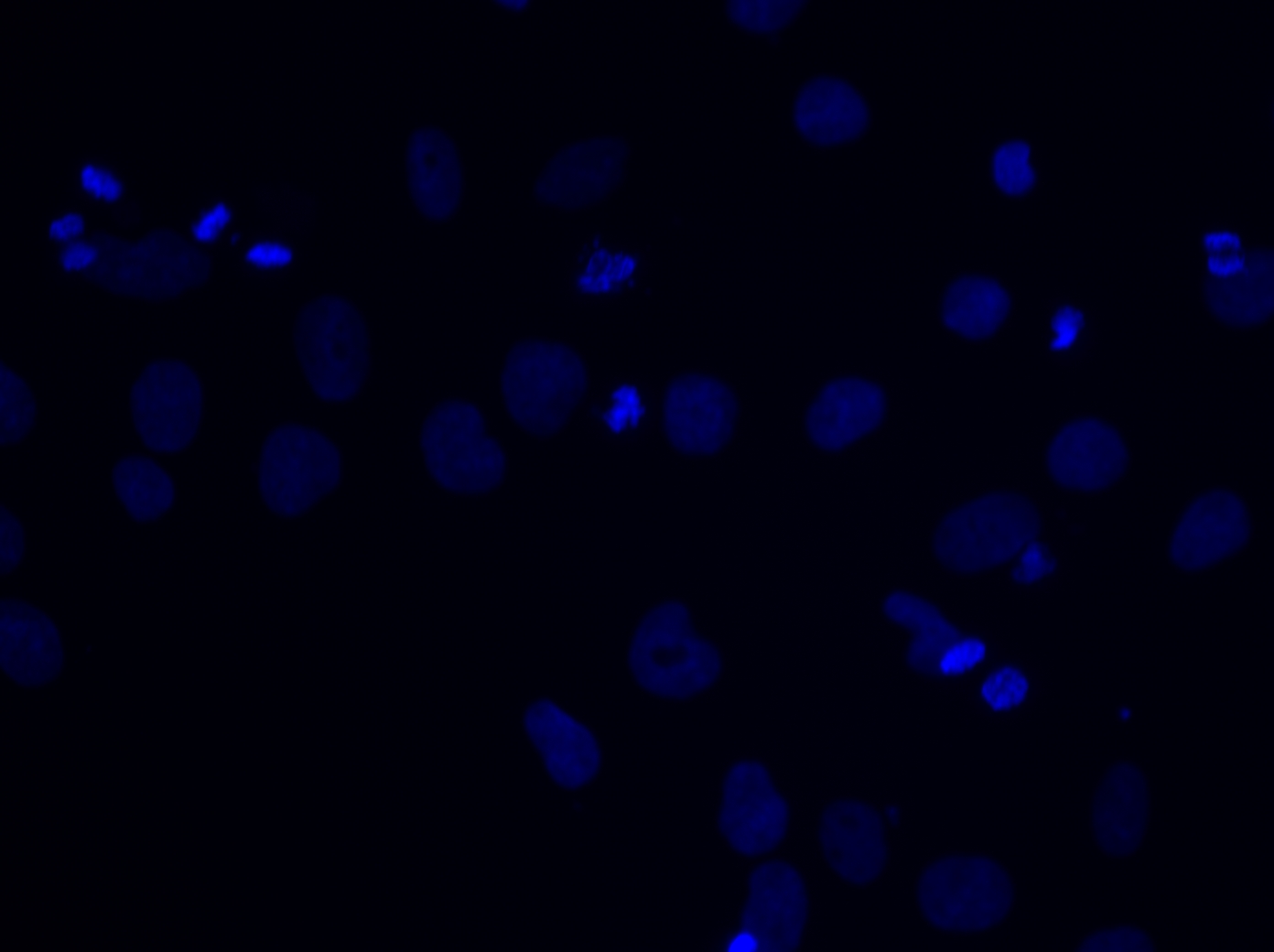

Supplement: Supplementary file 5 — Figure 4 raw data [file 44318_2025_532_MOESM5_ESM.zip › Figure 4/4F/KQ GFP-NuMA-KR_Image025_ch00.jpg]

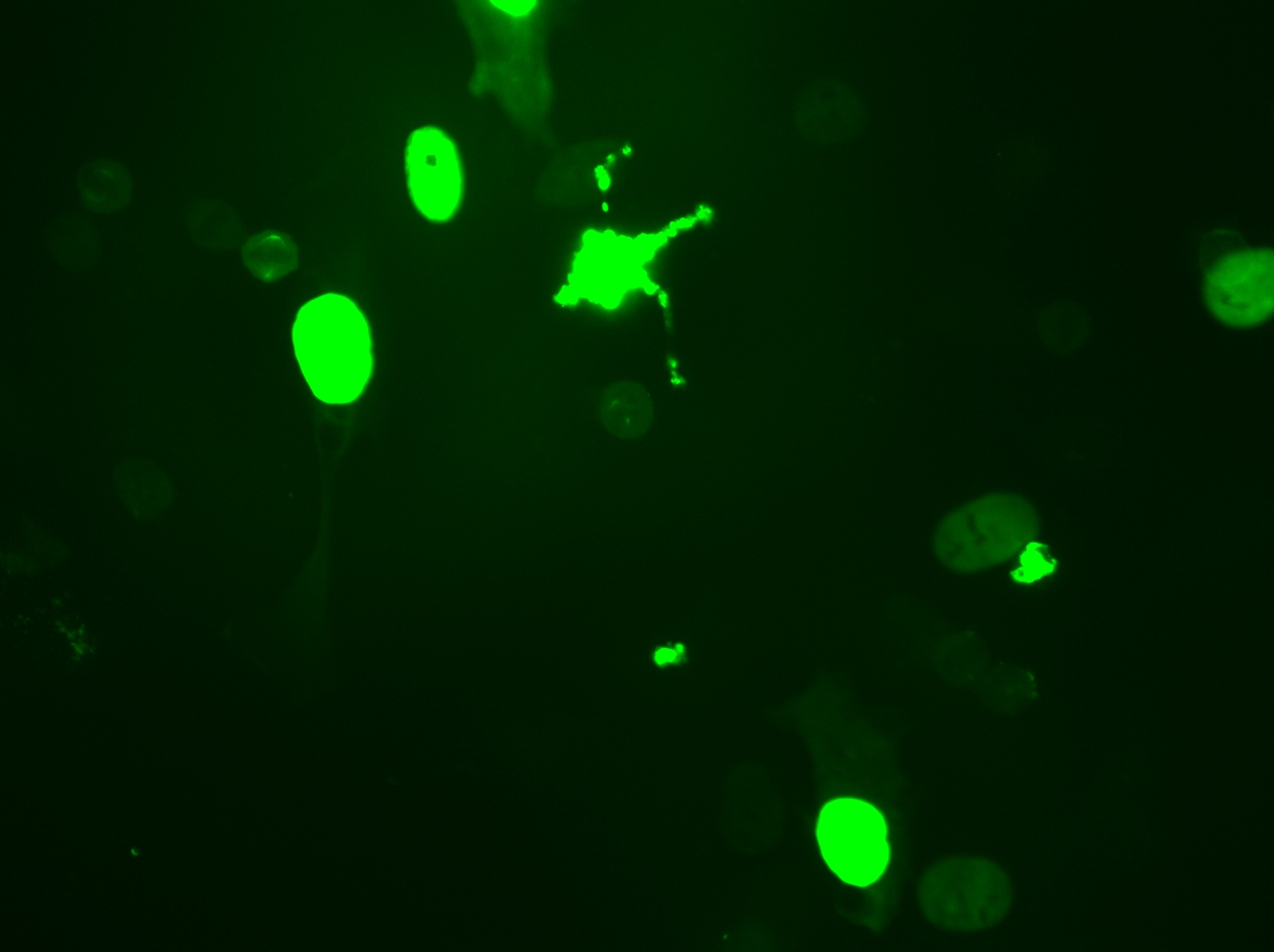

Supplement: Supplementary file 5 — Figure 4 raw data [file 44318_2025_532_MOESM5_ESM.zip › Figure 4/4F/KQ GFP-NuMA-KR_Image025_ch01.jpg]

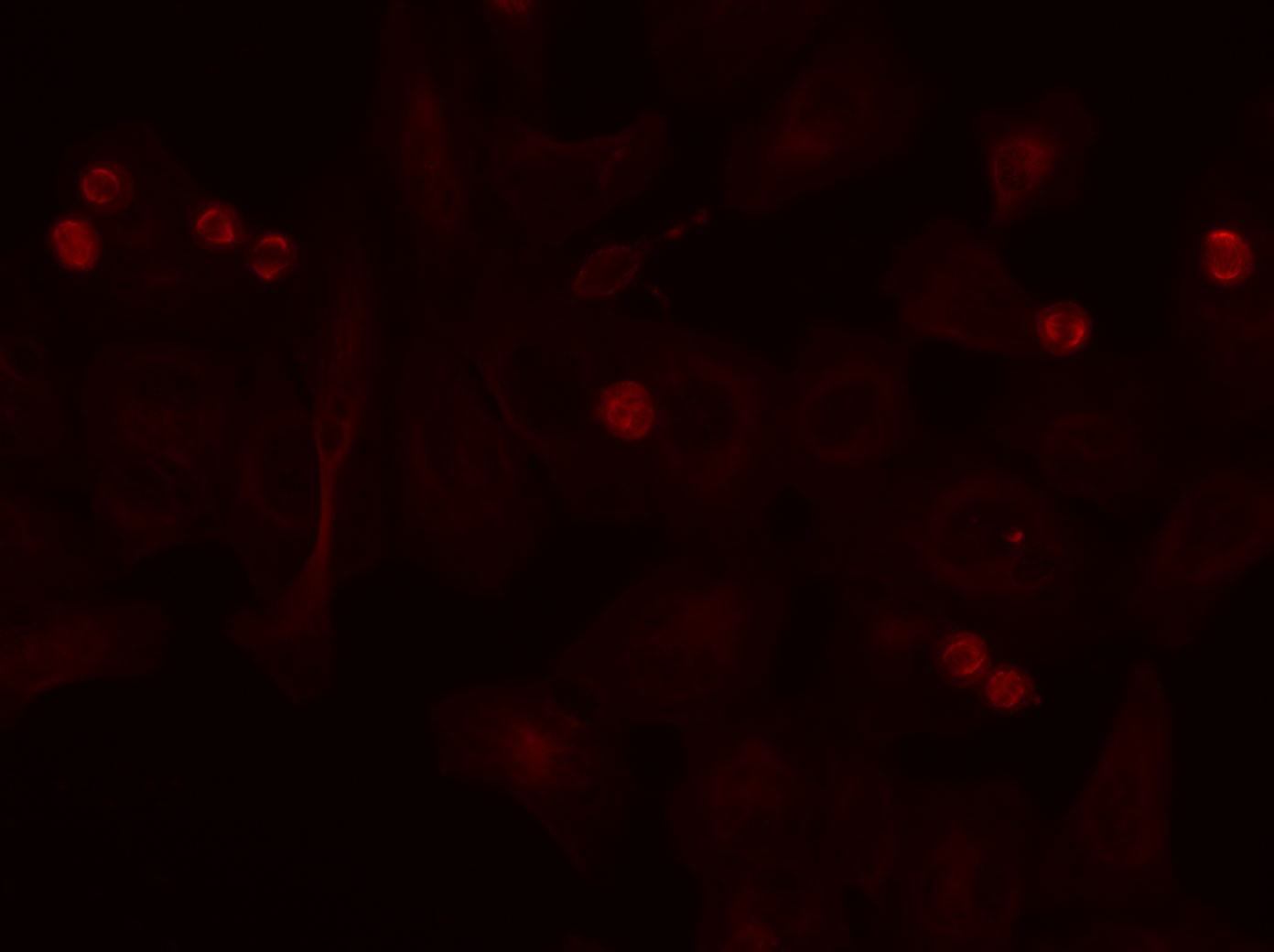

Supplement: Supplementary file 5 — Figure 4 raw data [file 44318_2025_532_MOESM5_ESM.zip › Figure 4/4F/KQ GFP-NuMA-KR_Image025_ch02.jpg]

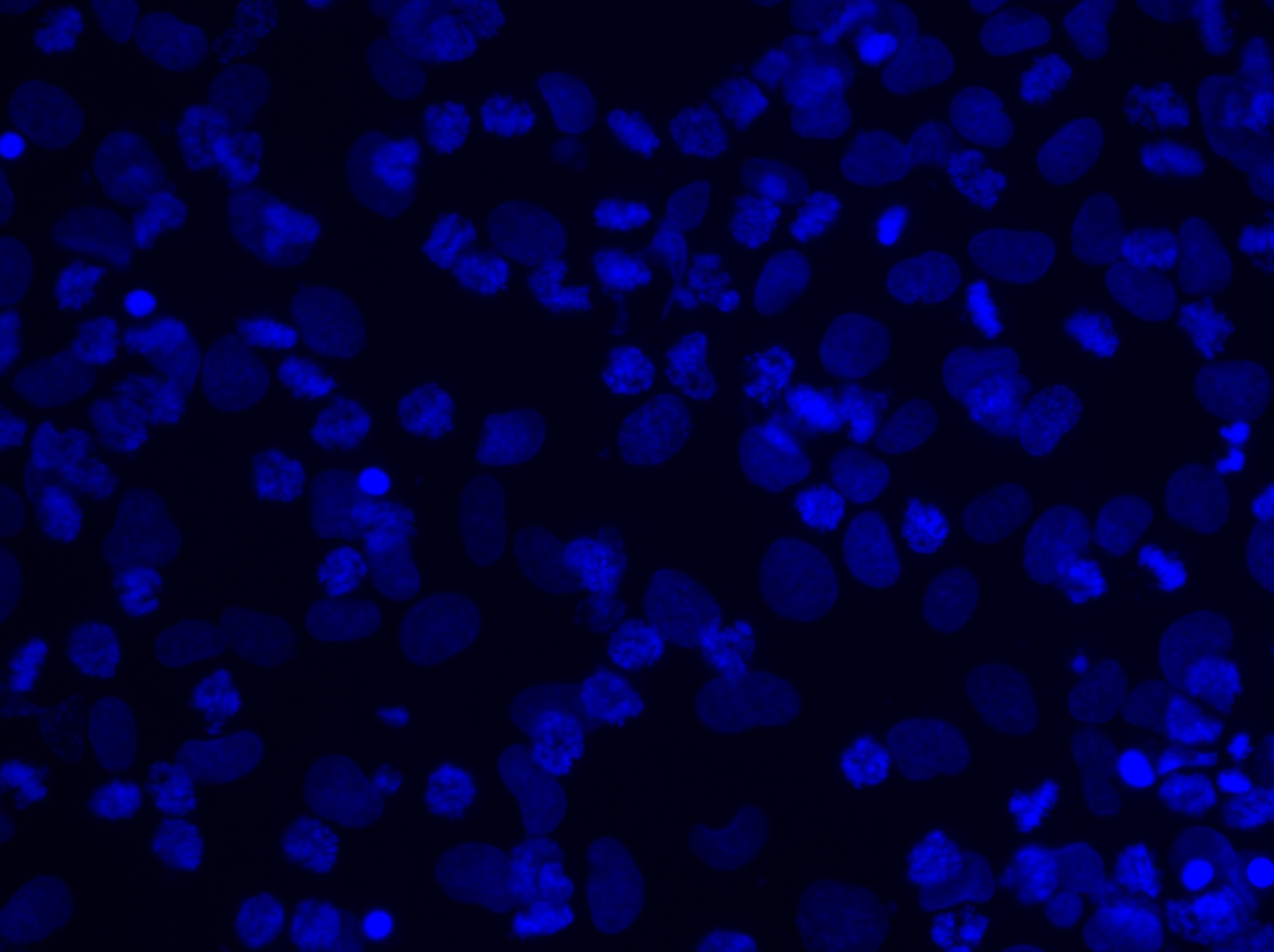

Supplement: Supplementary file 5 — Figure 4 raw data [file 44318_2025_532_MOESM5_ESM.zip › Figure 4/4F/KQ GFP-NuMA-KR-S1_Image039 Structures_ch00.jpg]

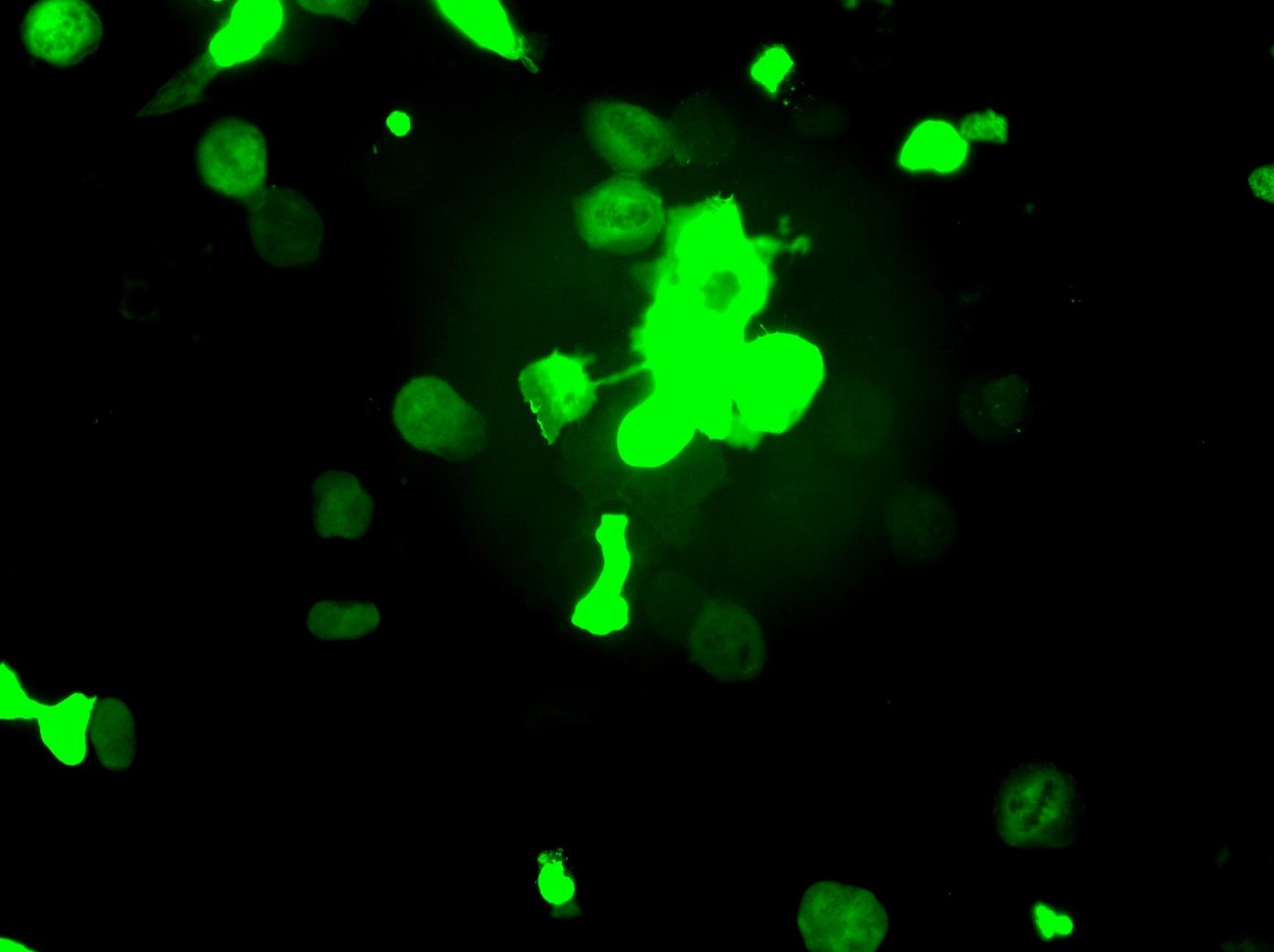

Supplement: Supplementary file 5 — Figure 4 raw data [file 44318_2025_532_MOESM5_ESM.zip › Figure 4/4F/KQ GFP-NuMA-KR-S1_Image039 Structures_ch01.jpg]

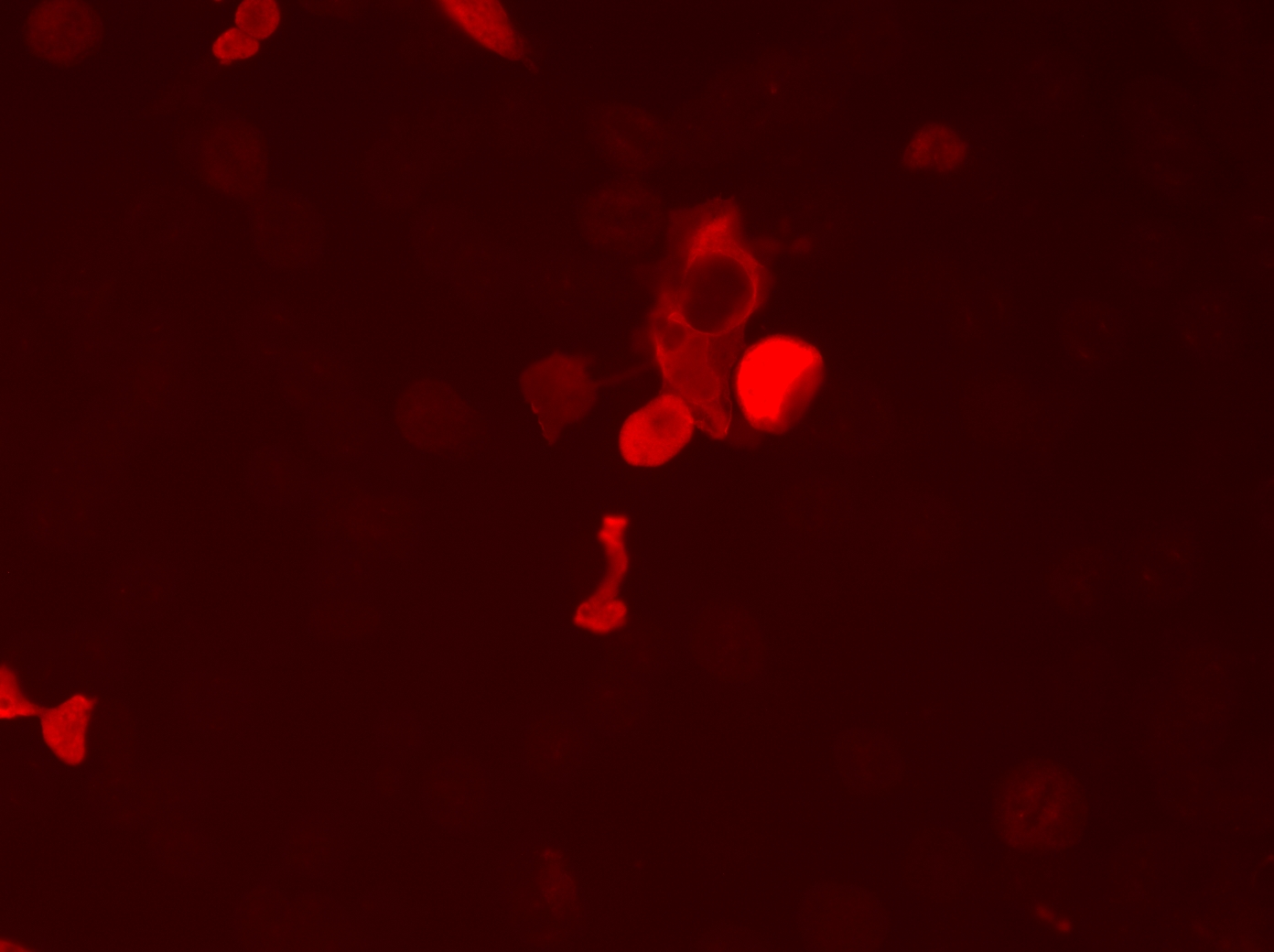

Supplement: Supplementary file 5 — Figure 4 raw data [file 44318_2025_532_MOESM5_ESM.zip › Figure 4/4F/KQ GFP-NuMA-KR-S1_Image039 Structures_ch02.jpg]

## Slide 1
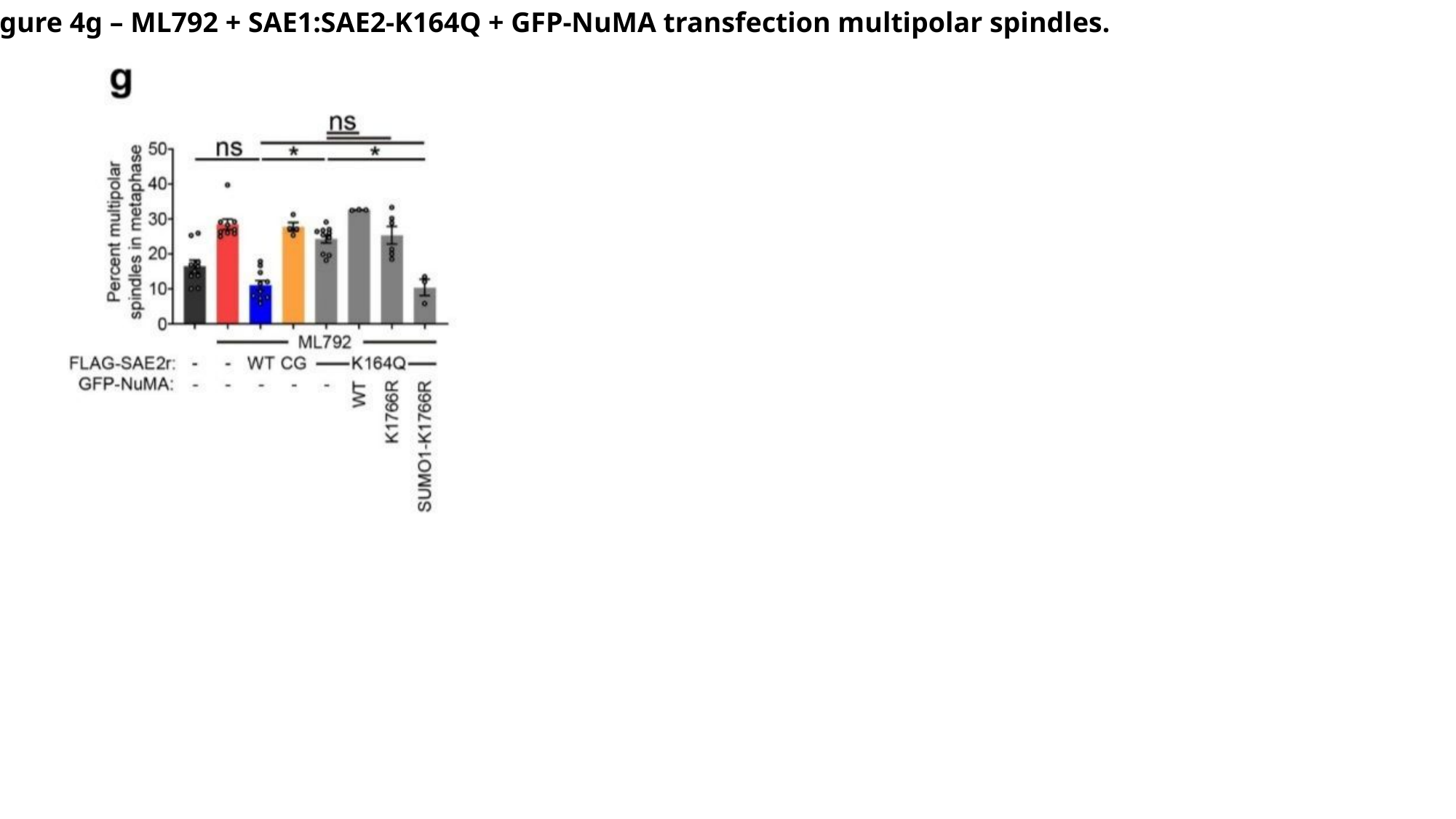

Figure 4g – ML792 + SAE1:SAE2-K164Q + GFP-NuMA transfection multipolar spindles.

Supplement: Supplementary file 5 — Figure 4 raw data [file 44318_2025_532_MOESM5_ESM.zip › Figure 4/4G/Figure 4g.pptx]

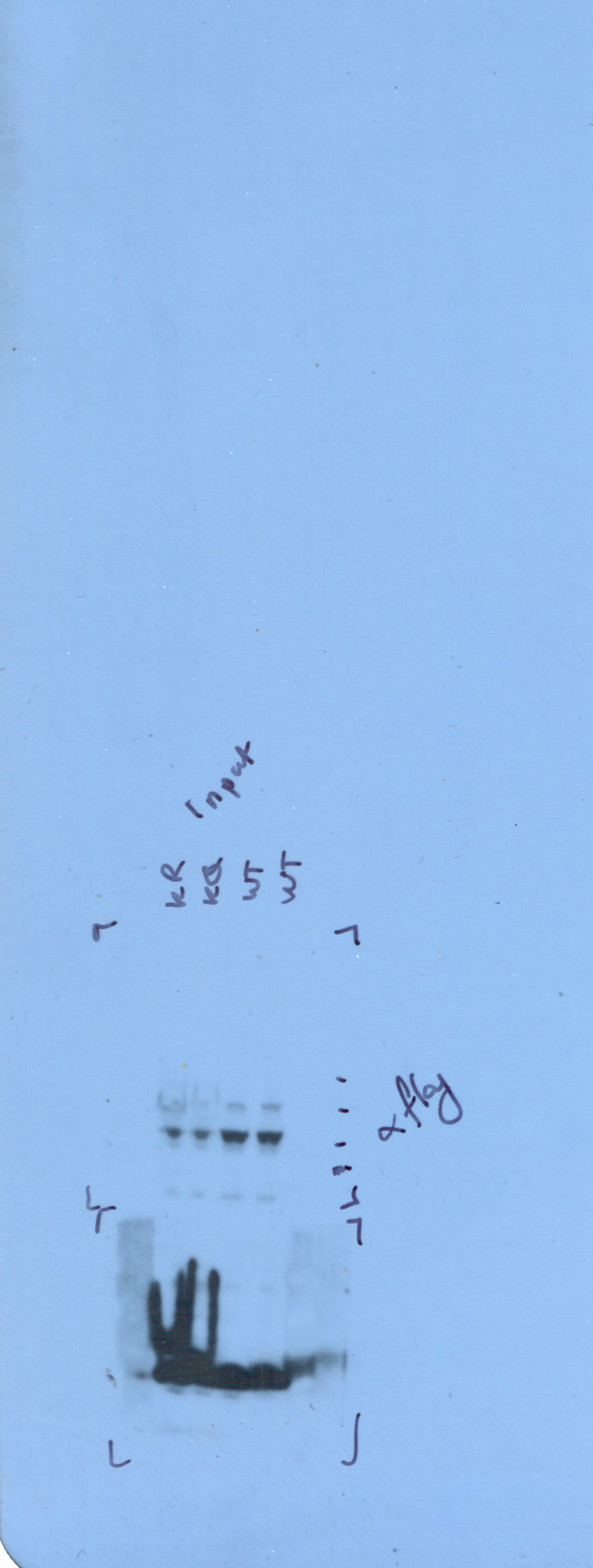

Supplement: Supplementary file 6 — Figure 3 raw data [file 44318_2025_532_MOESM6_ESM.zip › Figure 3/3A/Input flag.tif]

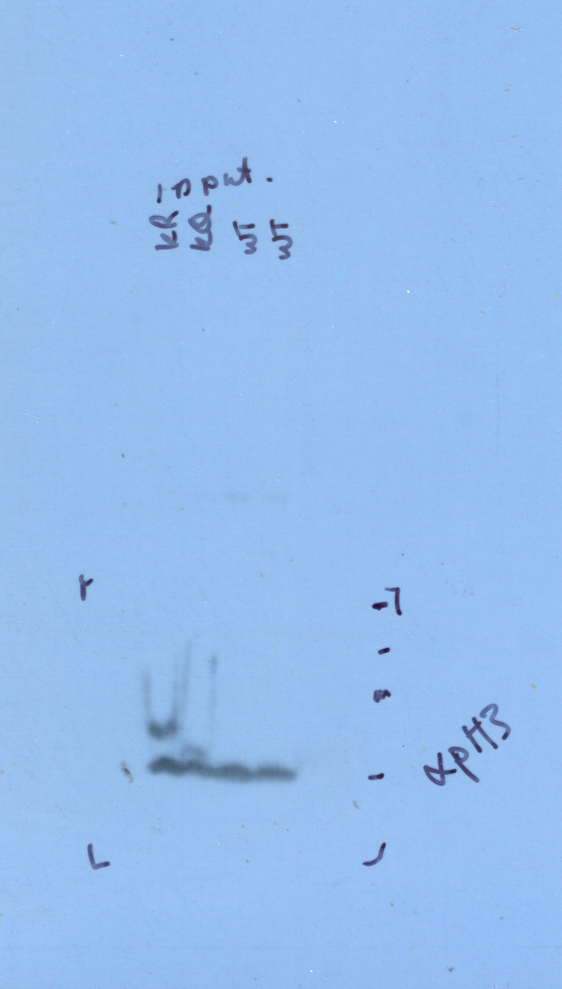

Supplement: Supplementary file 6 — Figure 3 raw data [file 44318_2025_532_MOESM6_ESM.zip › Figure 3/3A/Input ph3.tif]

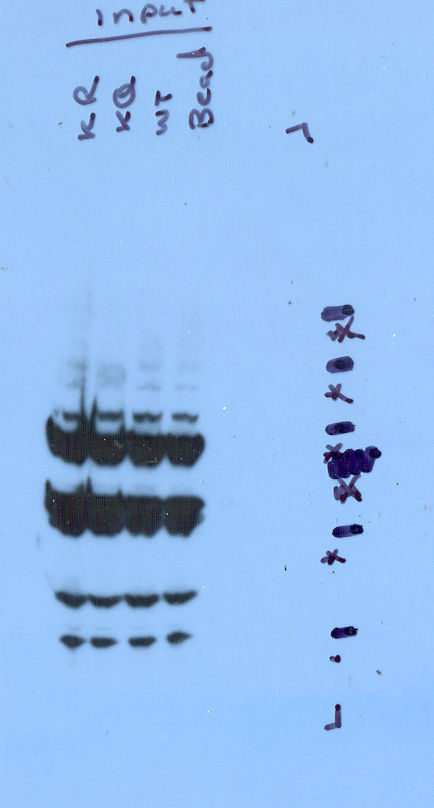

Supplement: Supplementary file 6 — Figure 3 raw data [file 44318_2025_532_MOESM6_ESM.zip › Figure 3/3A/Input SUMO1ep298.tif]

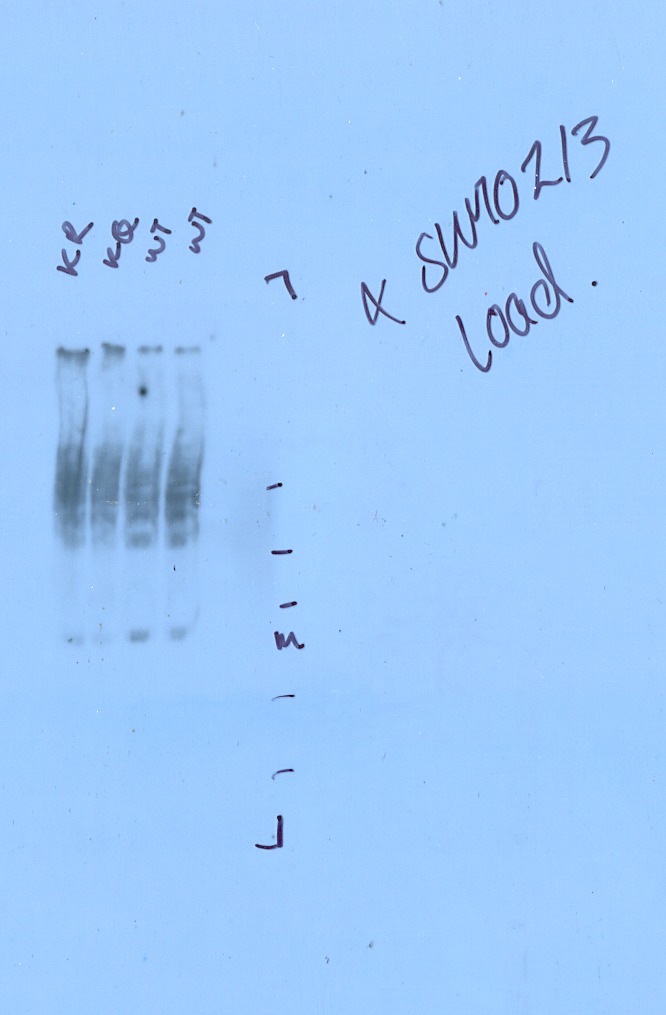

Supplement: Supplementary file 6 — Figure 3 raw data [file 44318_2025_532_MOESM6_ESM.zip › Figure 3/3A/Input SUMO2.3 8a2.tif]

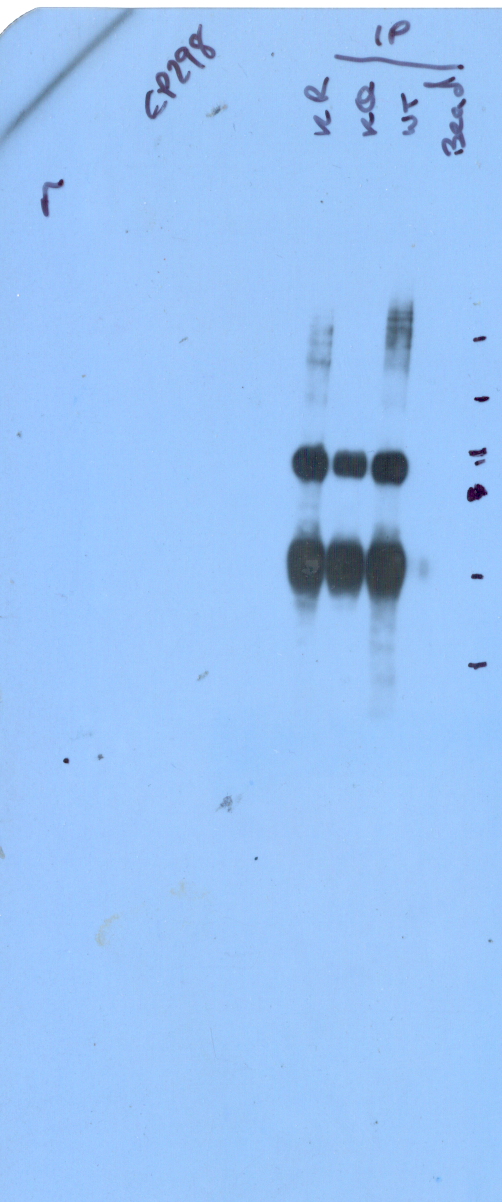

Supplement: Supplementary file 6 — Figure 3 raw data [file 44318_2025_532_MOESM6_ESM.zip › Figure 3/3A/Mitotic IP SUMO1 ep298.tif]

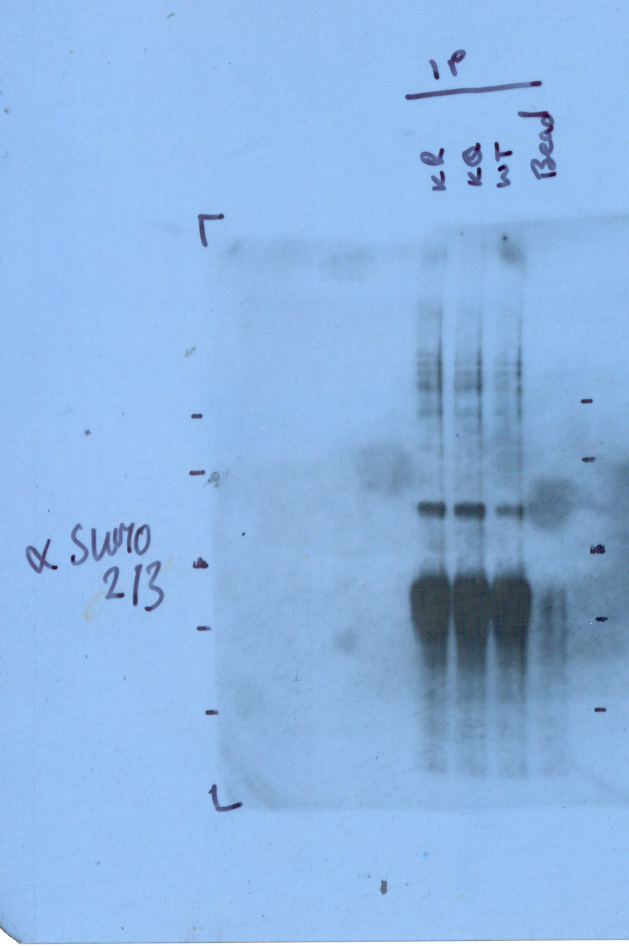

Supplement: Supplementary file 6 — Figure 3 raw data [file 44318_2025_532_MOESM6_ESM.zip › Figure 3/3A/Mitotic IP SUMO2.3 8a2.tif]

Figure 3a

Sumo1 IP  
Probed SUMO1 antibody Y299

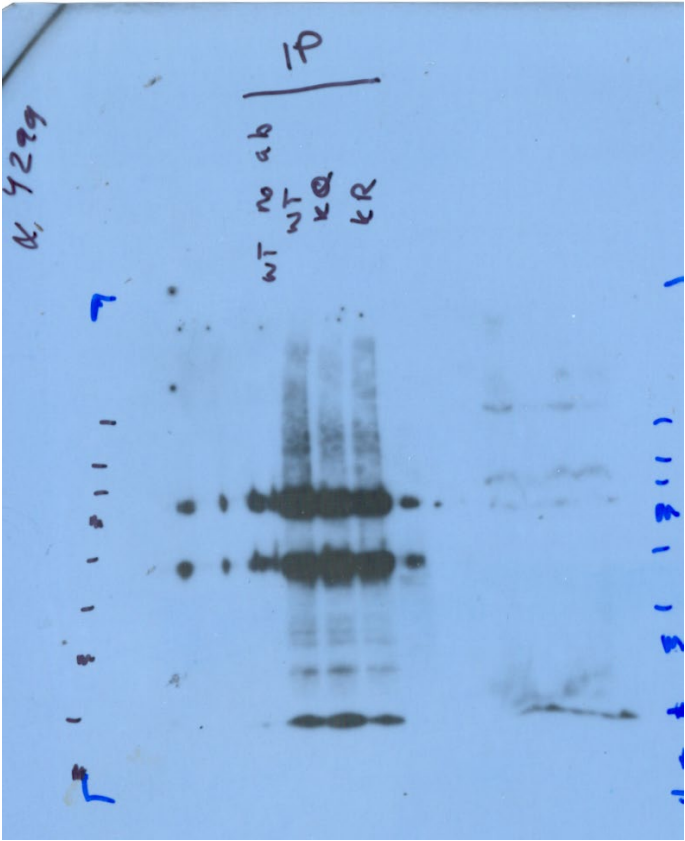

Sumo2/3 IP  
Probed SUMO1 antibody 8A2

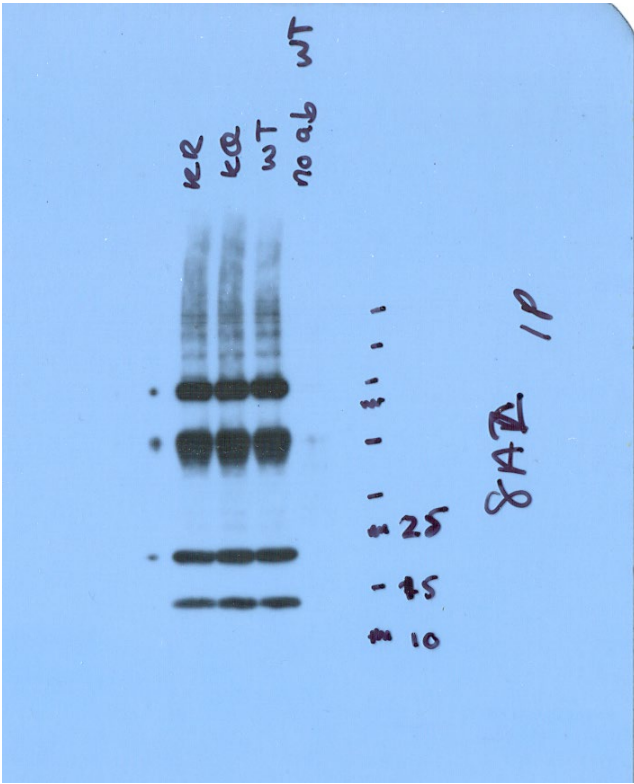

Input

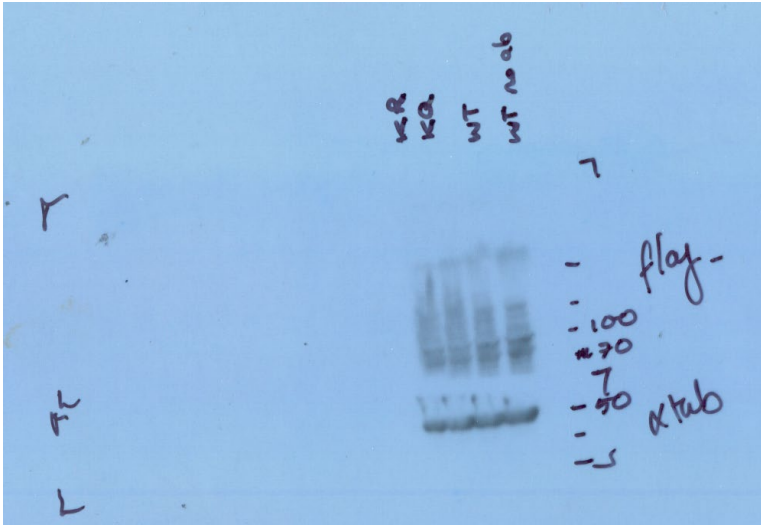

Supplement: Supplementary file 6 — Figure 3 raw data [file 44318_2025_532_MOESM6_ESM.zip › Figure 3/3A/new y299 IP/Figure 3a.pdf]
