# Supplementary material for: HDAC6-dependent deacetylation of SAE2 enhances SUMO1 conjugation for mitotic integrity
Source: EMBO J. 2025 Aug 20;44(19):5537–63. doi: 10.1038/s44318-025-00532-y (PMC12489036; doi:10.1038/s44318-025-00532-y)
Supplement: Supplementary file 4 — Figure 2 raw data [file 44318_2025_532_MOESM4_ESM.zip › Figure 2/2C/Figure 2c.pptx]

## Slide 1
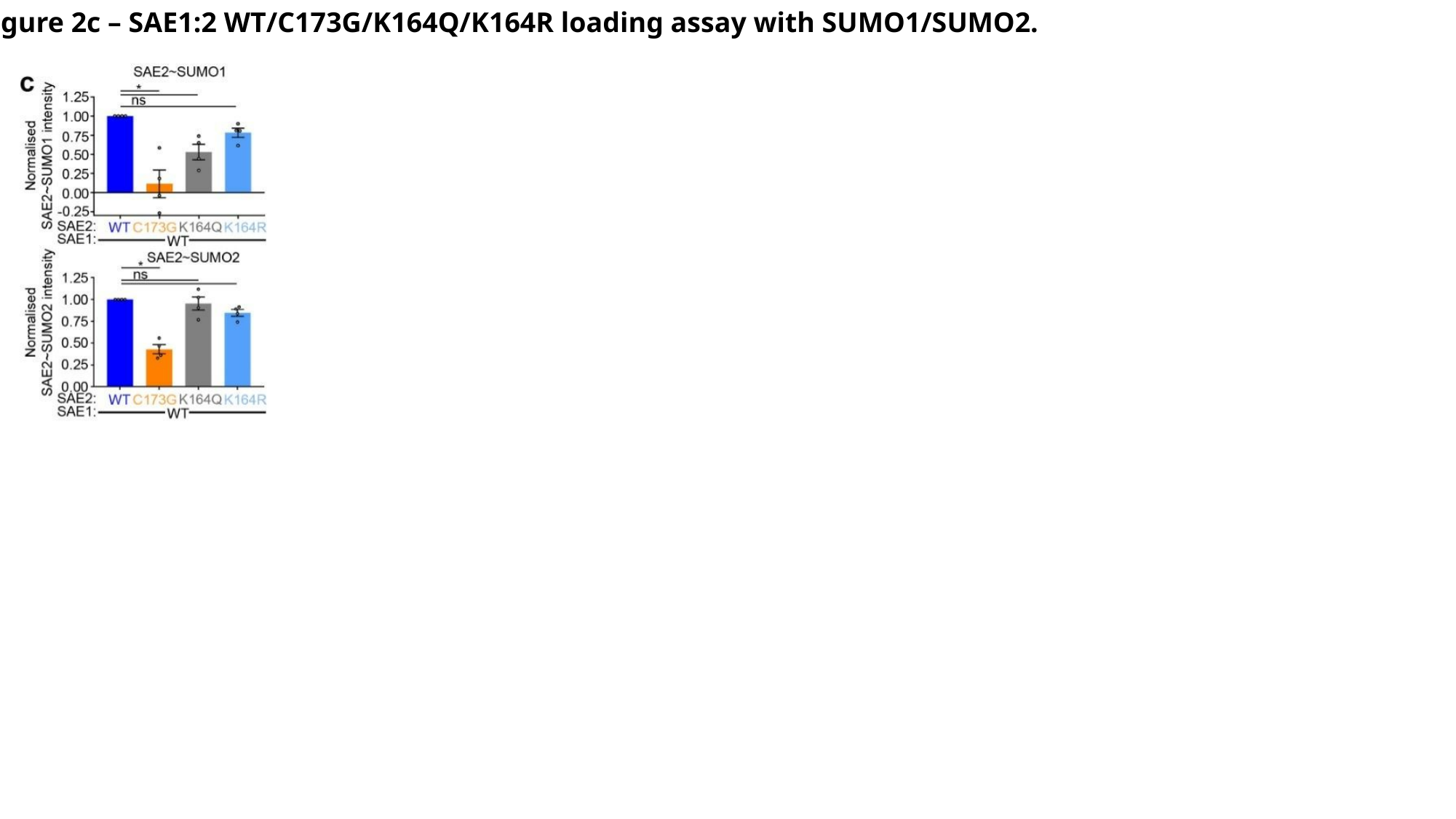

Figure 2c – SAE1:2 WT/C173G/K164Q/K164R loading assay with SUMO1/SUMO2.

## Slide 2
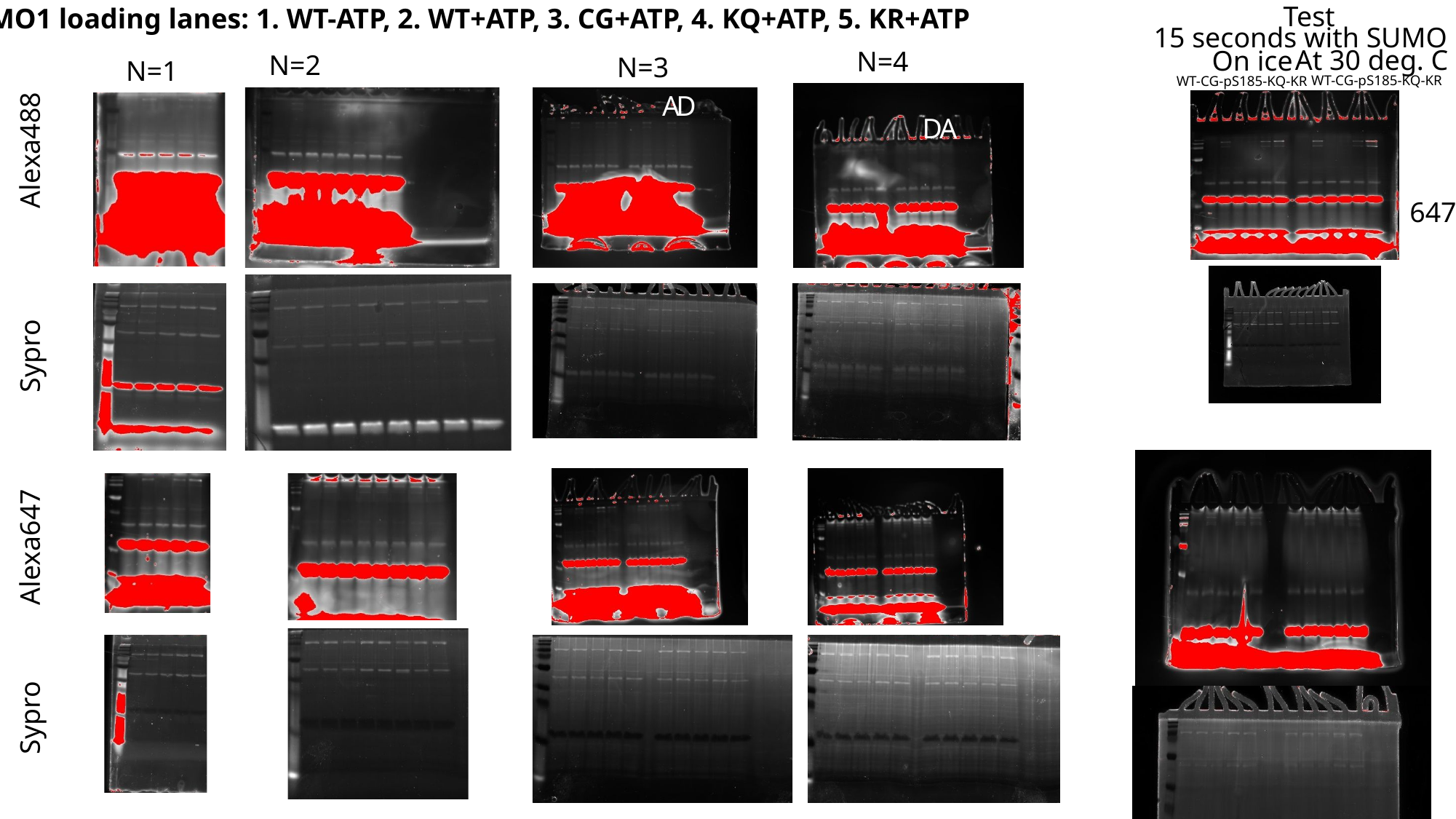

Test
SUMO1 loading lanes: 1. WT-ATP, 2. WT+ATP, 3. CG+ATP, 4. KQ+ATP, 5. KR+ATP
15 seconds with SUMO
At 30 deg. C
On ice
N=4
N=2
N=3
N=1
WT-CG-pS185-KQ-KR
WT-CG-pS185-KQ-KR
A
D
A
D
Alexa488
647
Sypro
Alexa647
Sypro
