# Supplementary material for: HDAC6-dependent deacetylation of SAE2 enhances SUMO1 conjugation for mitotic integrity
Source: EMBO J. 2025 Aug 20;44(19):5537–63. doi: 10.1038/s44318-025-00532-y (PMC12489036; doi:10.1038/s44318-025-00532-y)

2d. Confirmation of thioester formation by fluorescent SUMO proteins.SDS-PAGE gels from *in vitro* thioester formation data in Fig. 2d. Reactions comprised 1 µM SUMO1-C52A-S9C-Alexa488 and/or 1 µM SUMO2-C48A-A2C-Alexa647, 200 nM SAE1:SAE2, and 5 mM ATP, incubated at 30°C for 10 minutes. Reactions 1 and 4 also contained 100 mM DTT to assess thioester formation. Reactions 2 and 5 were the standard reaction without DTT. Reactions 3 and 6 were the ATP minus control reaction without DTT addition. The SUMO1-C52A-S9C-Alexa488 and SUMO2-C48A-A2C-Alexa647 loading were observed with excitation wavelengths of 493 nm and 647 nm, respectively, with bands at ~120 kDa taken to be SAE2~SUMO.

SAE1-Fluoresecnce SUMO loading with and without DTT and ATP

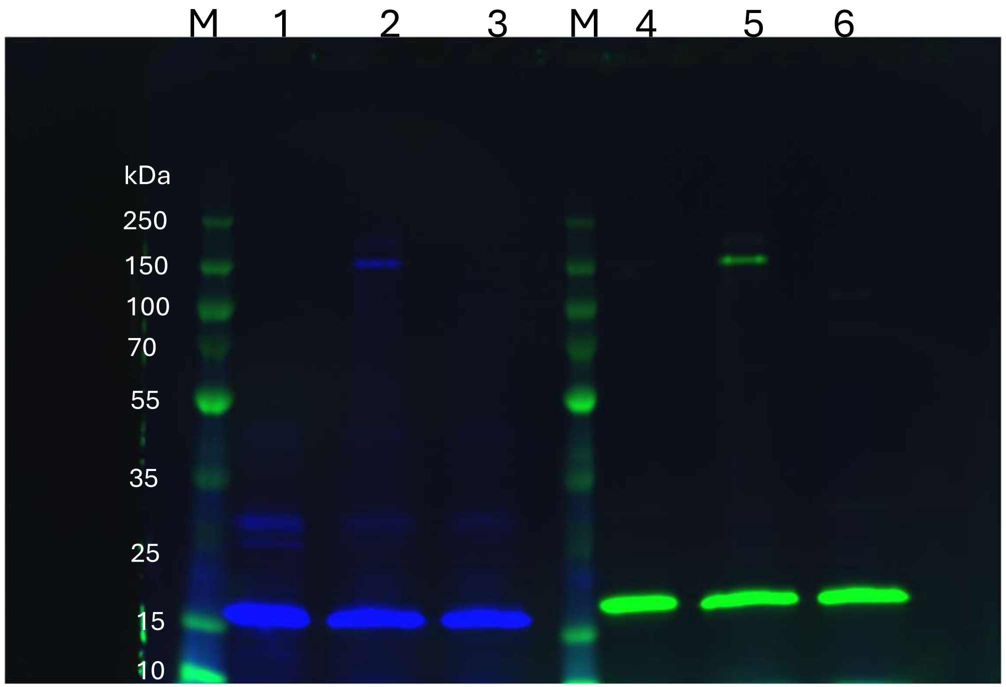

M – Marker

1 - SAE1/2 (200 nM) + SUMO1 C52A 488 (1 uM) + ATP (5 mM) + 100 mM DTT

2 - SAE1/2 (200 nM) + SUMO1 C52A 488 (1 uM) + ATP (5 mM)

3 - SAE1/2 (200 nM) + SUMO1 C52A 488 (1 uM)

4 - SAE1/2 (200 nM) + SUMO2 C48A 647 (1 uM) + ATP (5 mM) + 100 mM DTT

5 - SAE1/2 (200 nM) + SUMO2 C48A 647 (1 uM) + ATP (5 mM)

6 - SAE1/2 (200 nM) + SUMO2 C48A 647 (1 uM)

Dithiothreitol (DTT) has been used in the literature to assess the formation of thioester bonds

RAW image

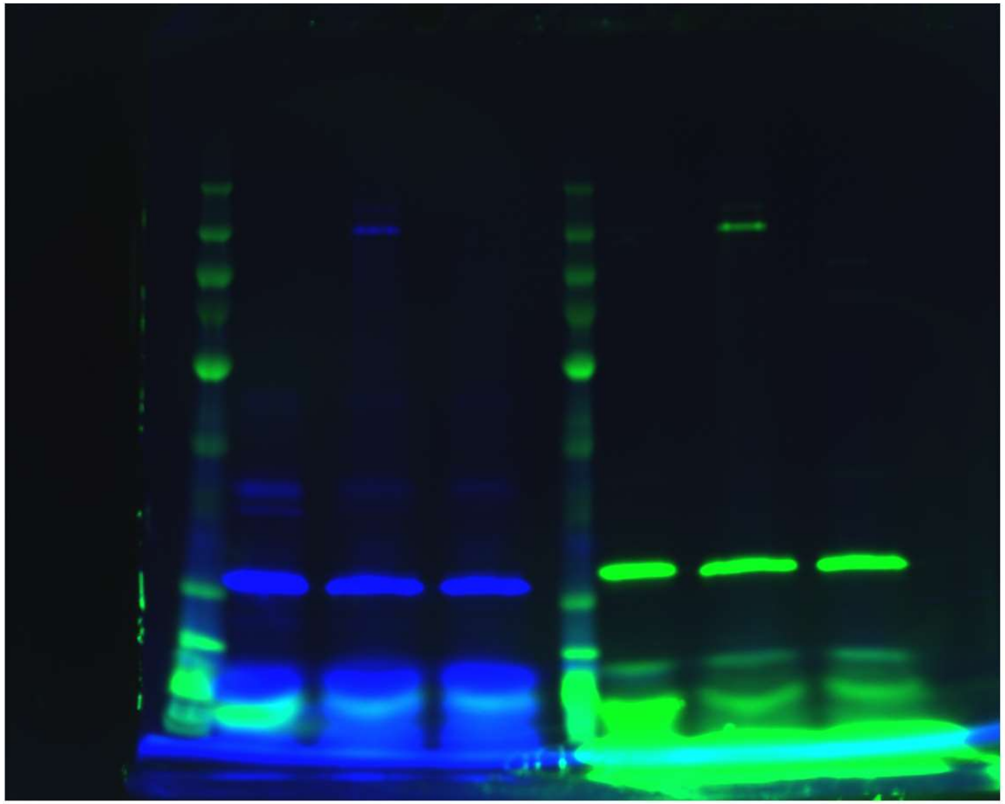

Supplement: Supplementary file 4 — Figure 2 raw data [file 44318_2025_532_MOESM4_ESM.zip › Figure 2/2D/Thioester formation figure 2D raw image.pdf]
