# Supplementary material for: HDAC6-dependent deacetylation of SAE2 enhances SUMO1 conjugation for mitotic integrity
Source: EMBO J. 2025 Aug 20;44(19):5537–63. doi: 10.1038/s44318-025-00532-y (PMC12489036; doi:10.1038/s44318-025-00532-y)
Supplement: Supplementary file 4 — Figure 2 raw data [file 44318_2025_532_MOESM4_ESM.zip › Figure 2/2F/Figure 2f.pptx]

## Slide 1
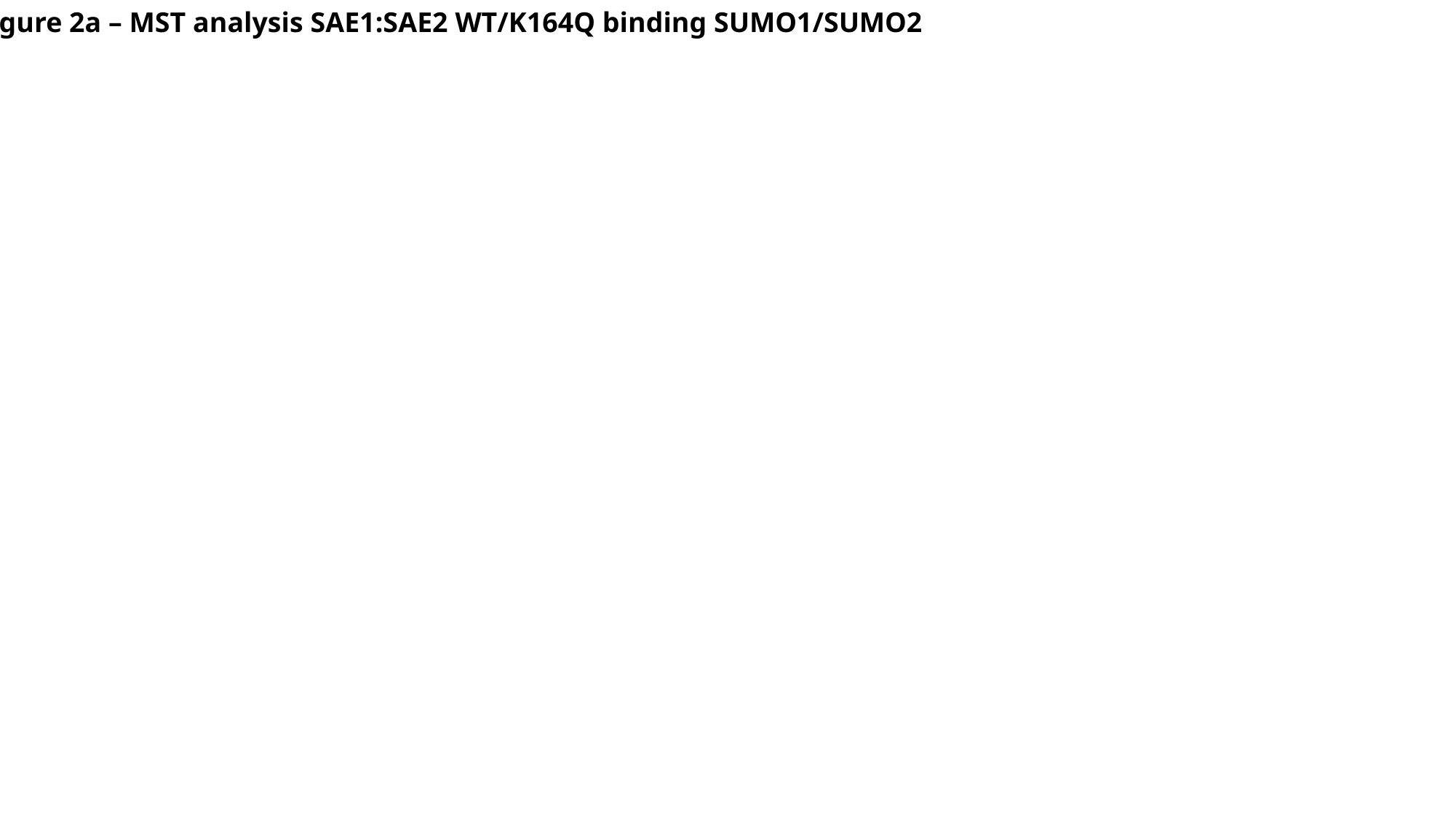

Figure 2a – MST analysis SAE1:SAE2 WT/K164Q binding SUMO1/SUMO2

## Slide 2
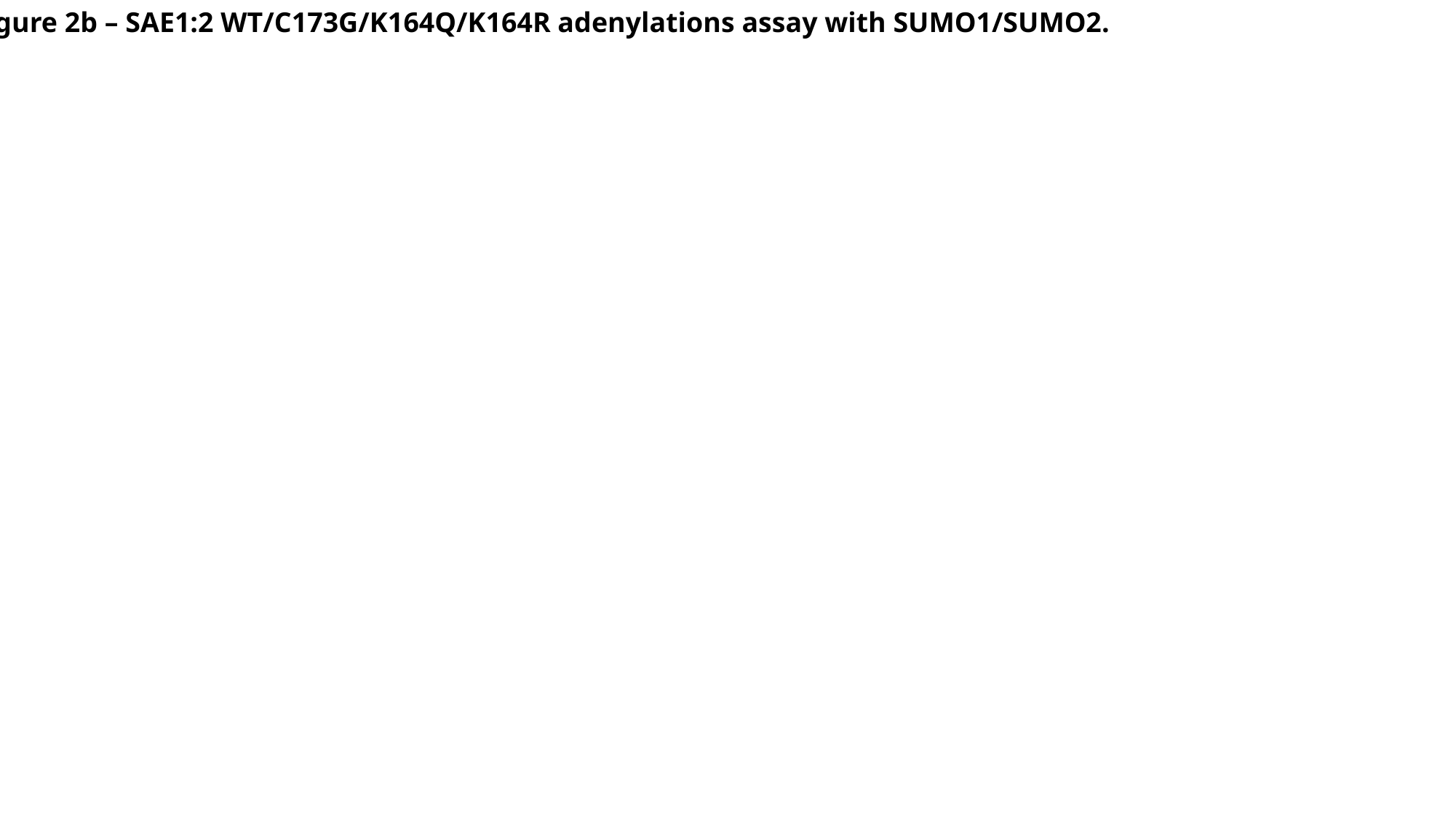

Figure 2b – SAE1:2 WT/C173G/K164Q/K164R adenylations assay with SUMO1/SUMO2.

## Slide 3
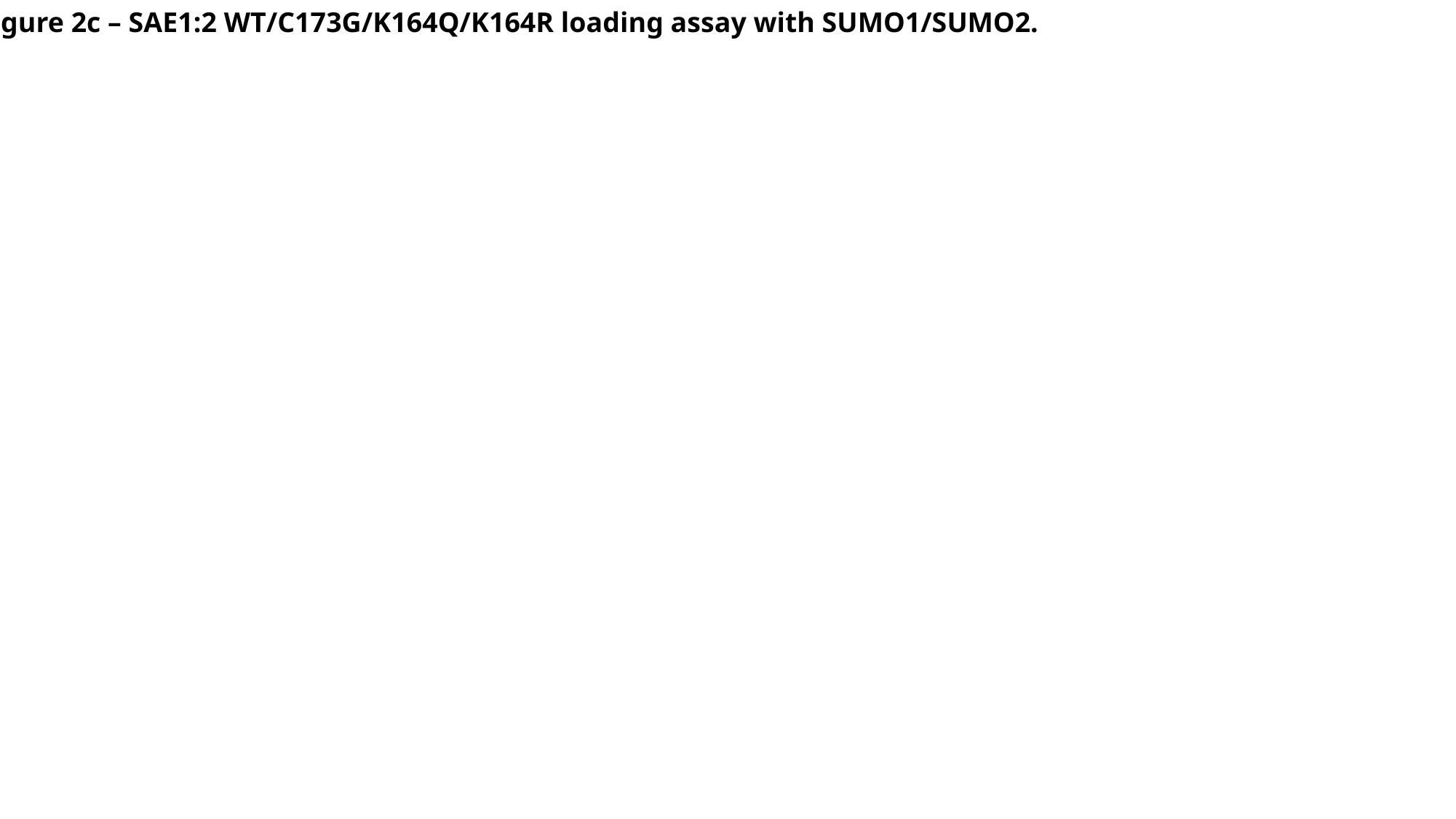

Figure 2c – SAE1:2 WT/C173G/K164Q/K164R loading assay with SUMO1/SUMO2.

## Slide 4
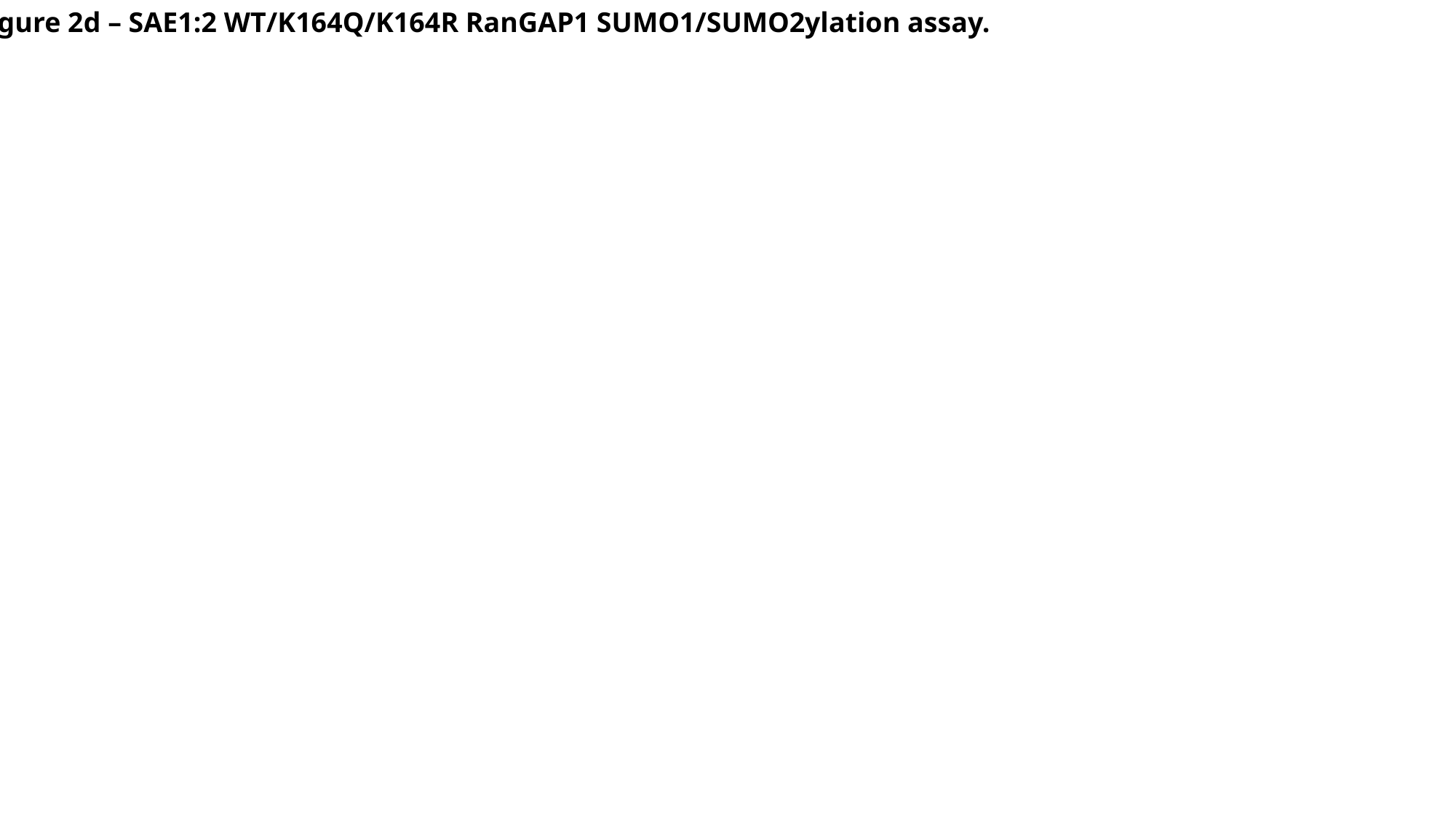

Figure 2d – SAE1:2 WT/K164Q/K164R RanGAP1 SUMO1/SUMO2ylation assay.

## Slide 5
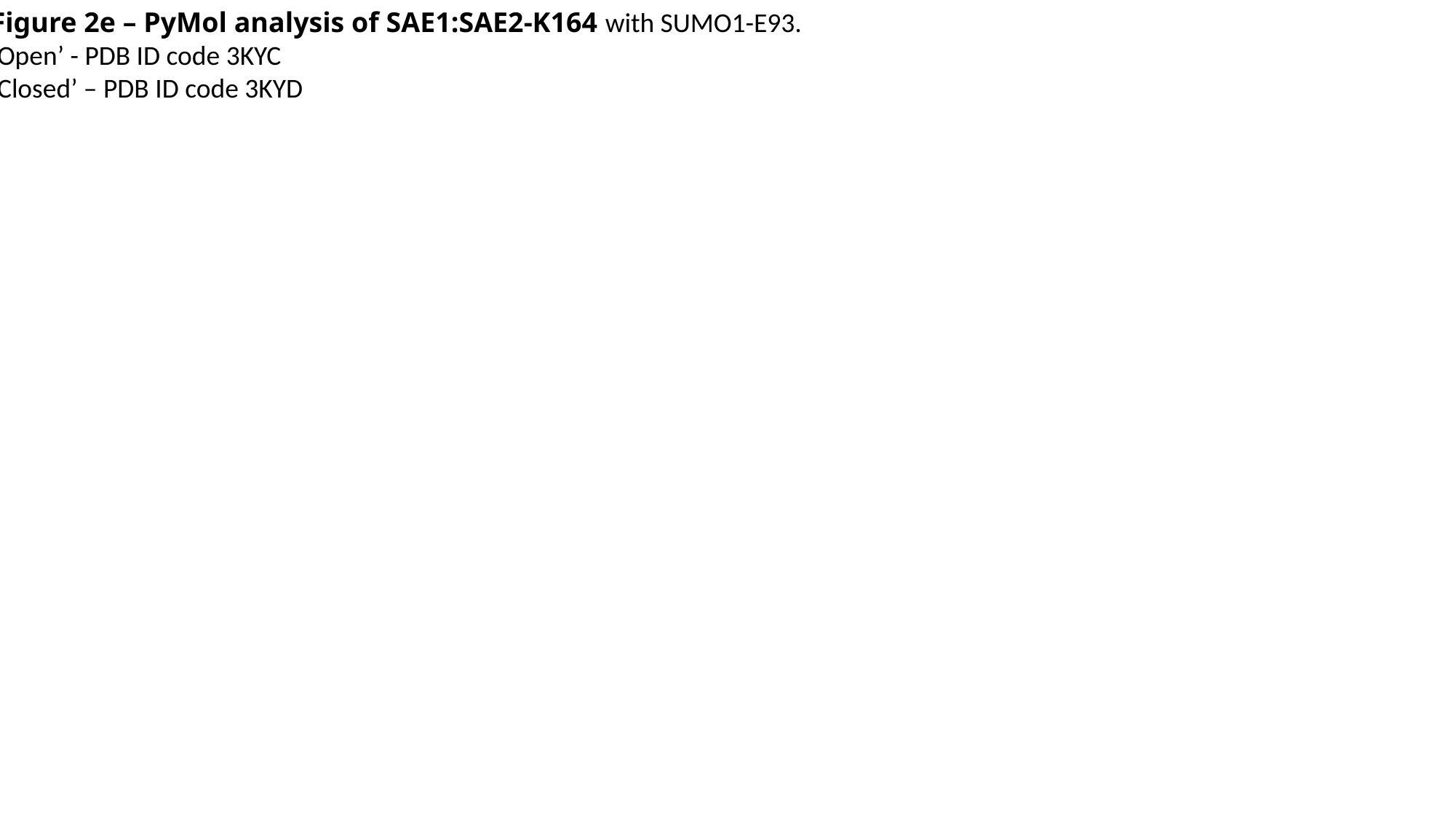

Figure 2e – PyMol analysis of SAE1:SAE2-K164 with SUMO1-E93.
‘Open’ - PDB ID code 3KYC
‘Closed’ – PDB ID code 3KYD

## Slide 6
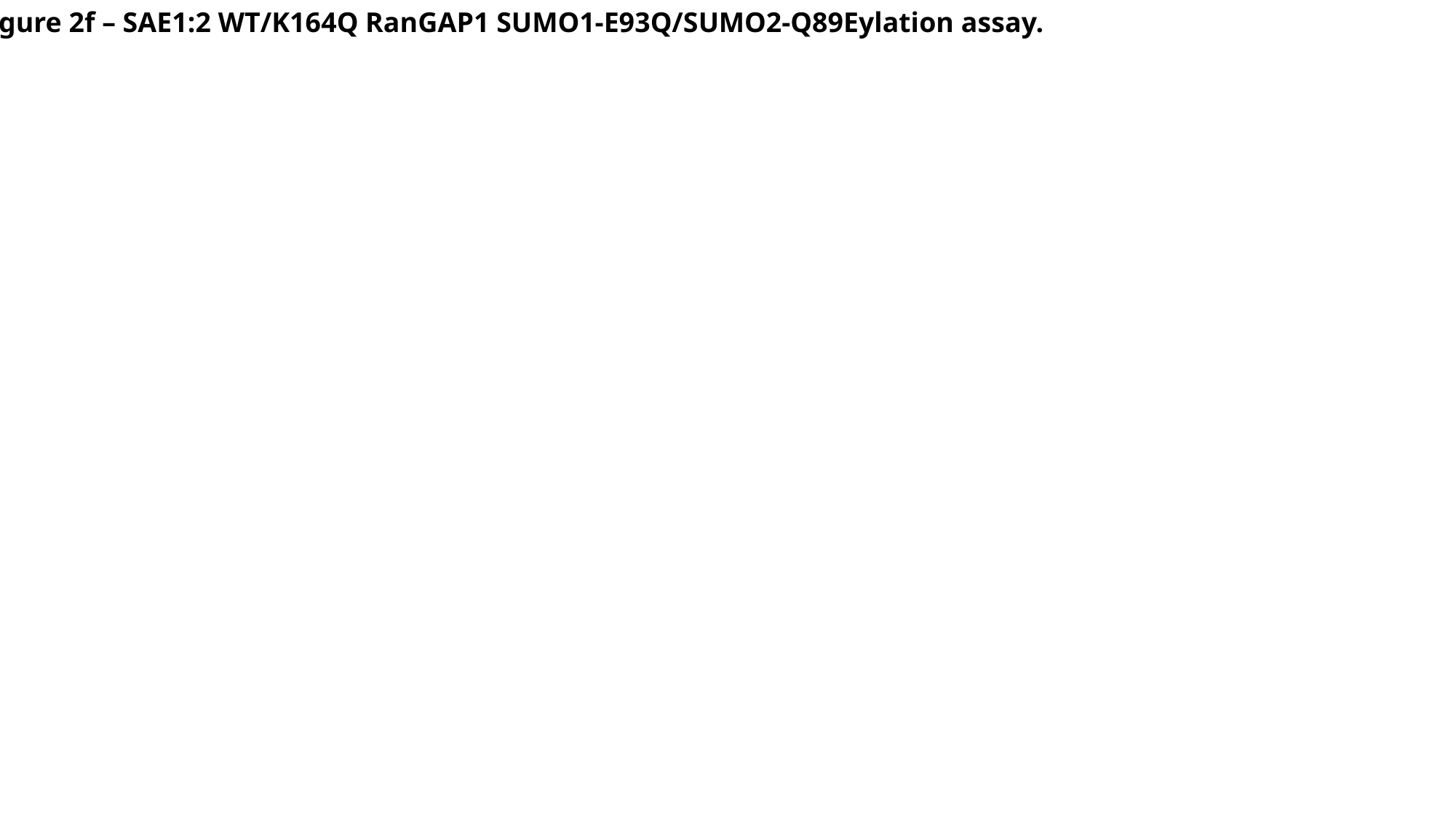

Figure 2f – SAE1:2 WT/K164Q RanGAP1 SUMO1-E93Q/SUMO2-Q89Eylation assay.
