# Supplementary material for: HDAC6-dependent deacetylation of SAE2 enhances SUMO1 conjugation for mitotic integrity
Source: EMBO J. 2025 Aug 20;44(19):5537–63. doi: 10.1038/s44318-025-00532-y (PMC12489036; doi:10.1038/s44318-025-00532-y)
Supplement: Supplementary file 6 — Figure 3 raw data [file 44318_2025_532_MOESM6_ESM.zip › Figure 3/3A/Figure 3a..pdf]

Figure 3a

SUMO1 IP
Protein A/G beads bound to Y299 antibody
Probed with EP298

IP SUMO1

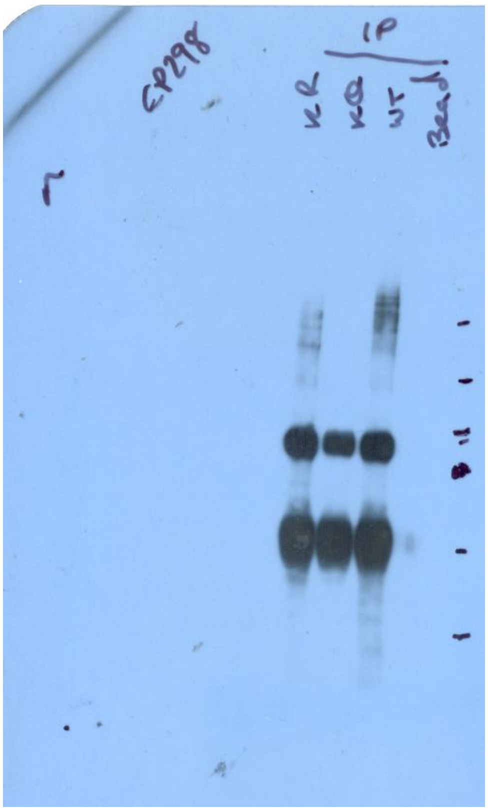

INPUT SUMO1

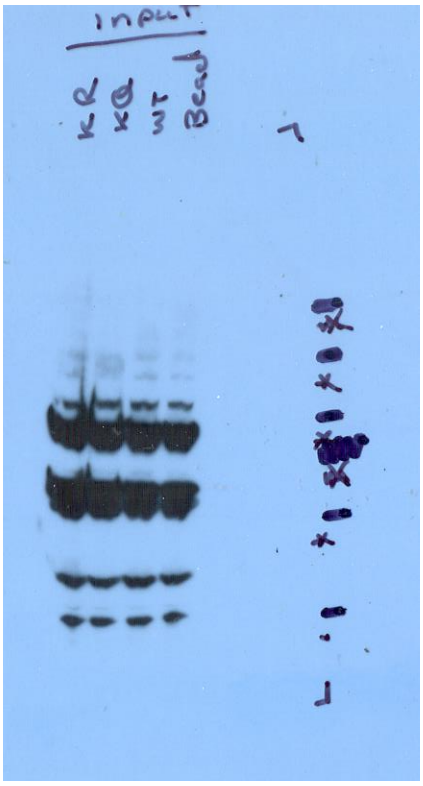

α SUMO1
(EP298)

SUMO2/3 IP
Protein A/G beads bound to SUMO2/3 8A2 antibody
Probed with 8A2

IP SUMO2/3

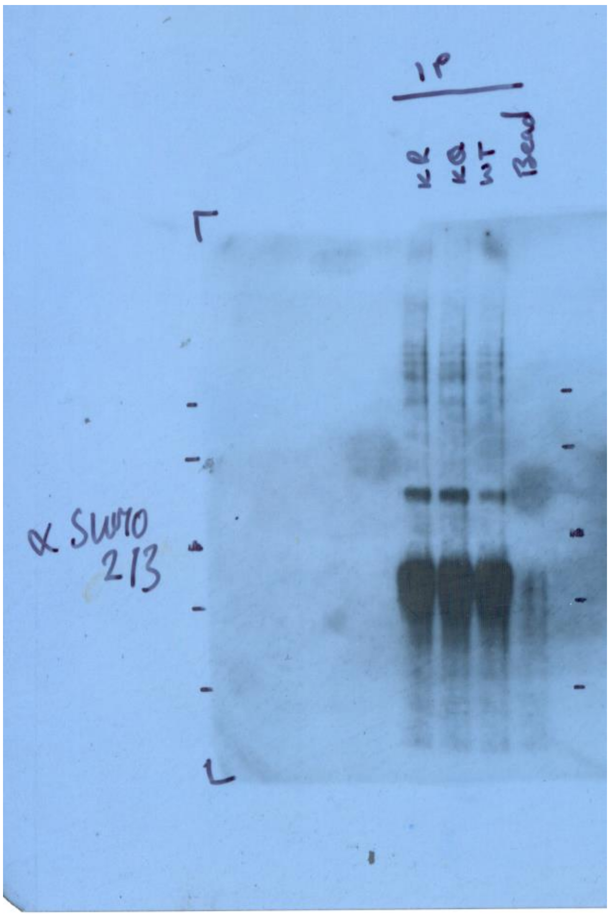

INPUT SUMO2/3

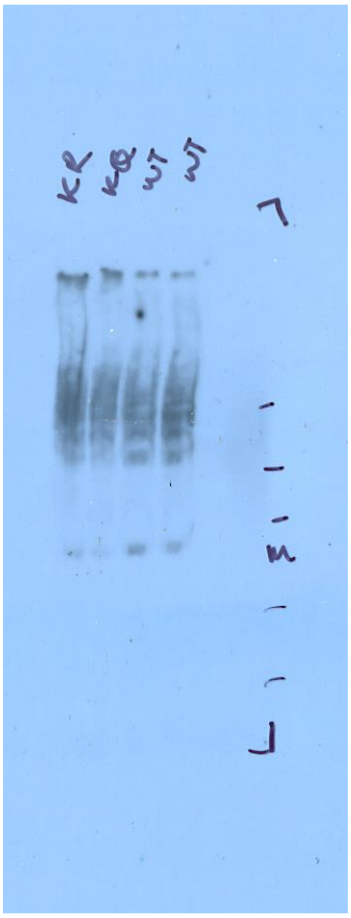

α SUMO2/3
(8A2)

Figure 3a

INPUTS for SUMO1 and 2/3

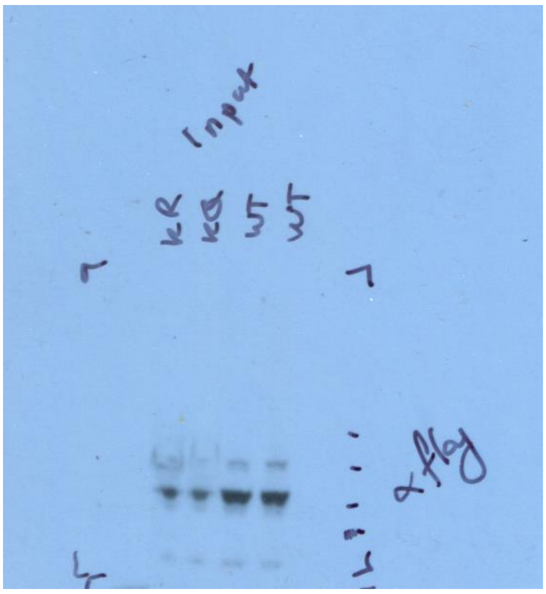

αFLAG

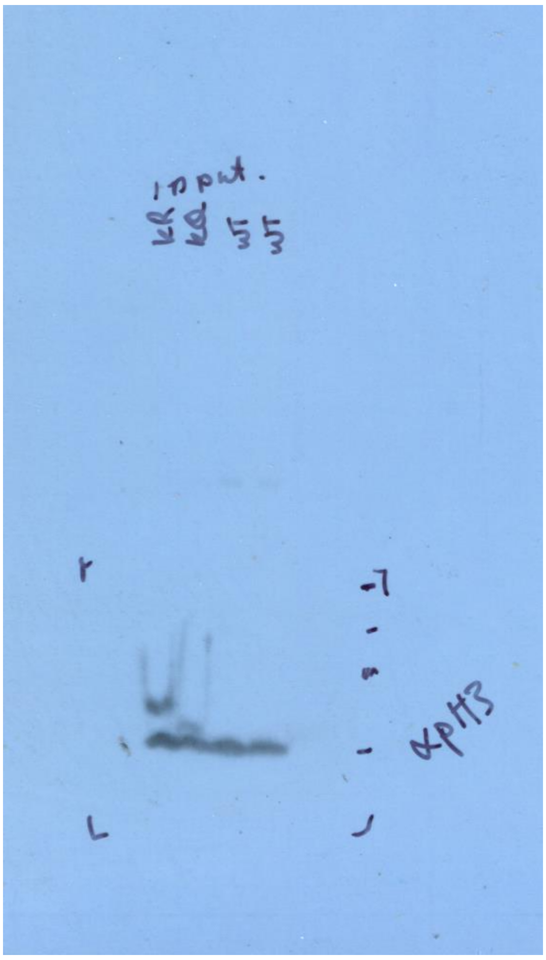

α phosH3
