# Supplementary figures and images for: HDAC6-dependent deacetylation of SAE2 enhances SUMO1 conjugation for mitotic integrity (part 2 of 2)
Source: EMBO J. 2025 Aug 20;44(19):5537–63. doi: 10.1038/s44318-025-00532-y (PMC12489036; doi:10.1038/s44318-025-00532-y)

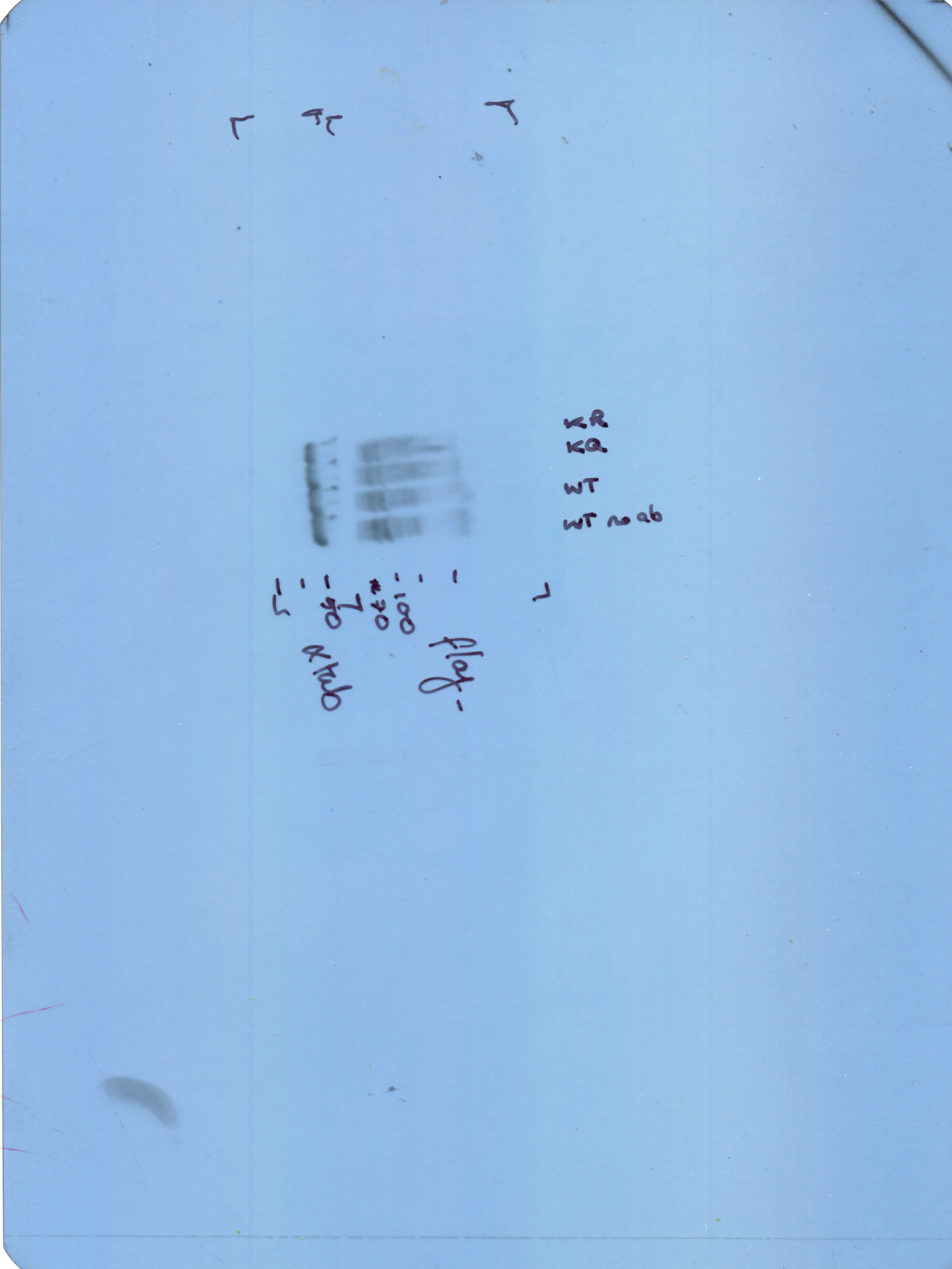

Supplement: Supplementary file 6 — Figure 3 raw data [file 44318_2025_532_MOESM6_ESM.zip › Figure 3/3A/new y299 IP/Input Sumo IP flag and tubulin.tif]

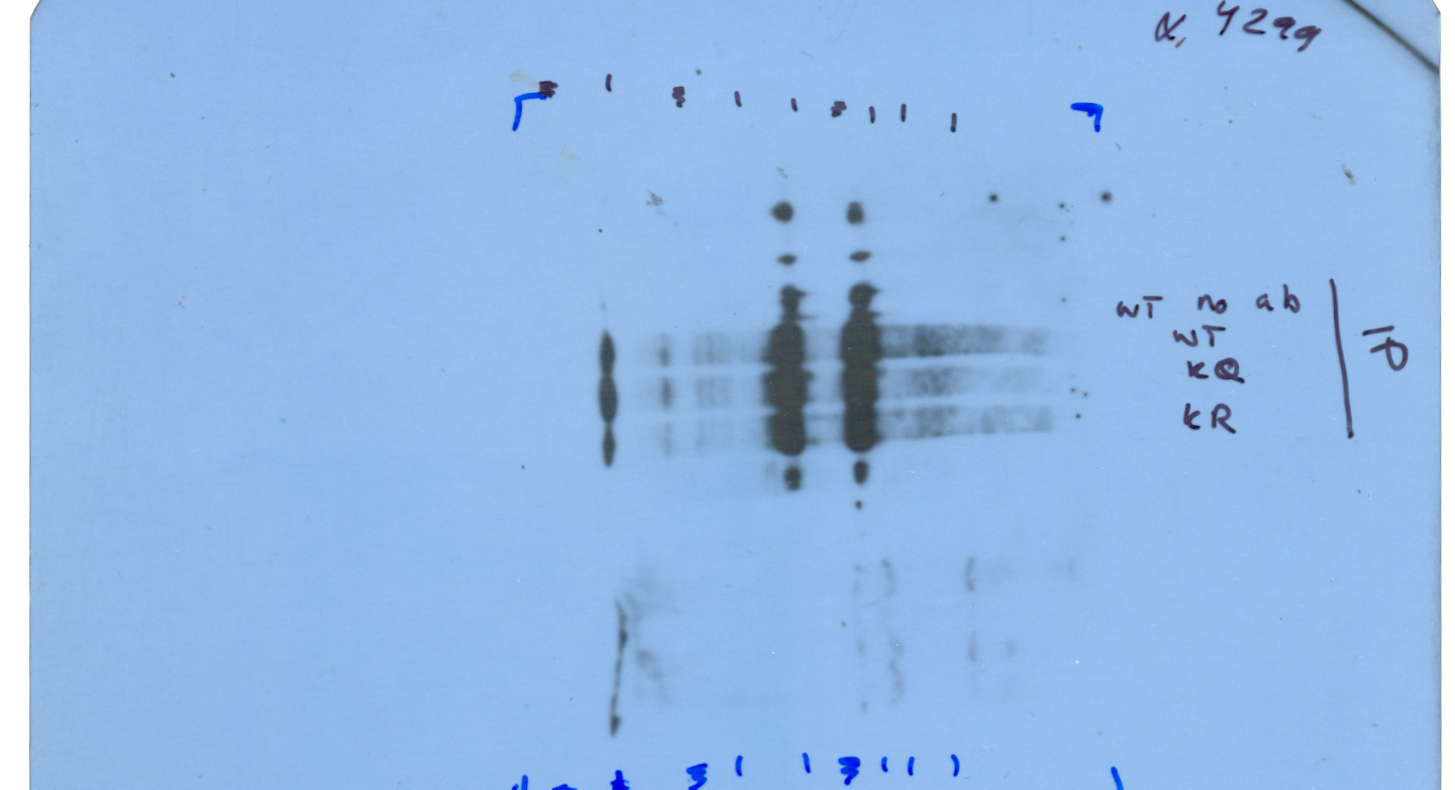

Supplement: Supplementary file 6 — Figure 3 raw data [file 44318_2025_532_MOESM6_ESM.zip › Figure 3/3A/new y299 IP/Mitotic SUMO1 IP probed Y299.tif]

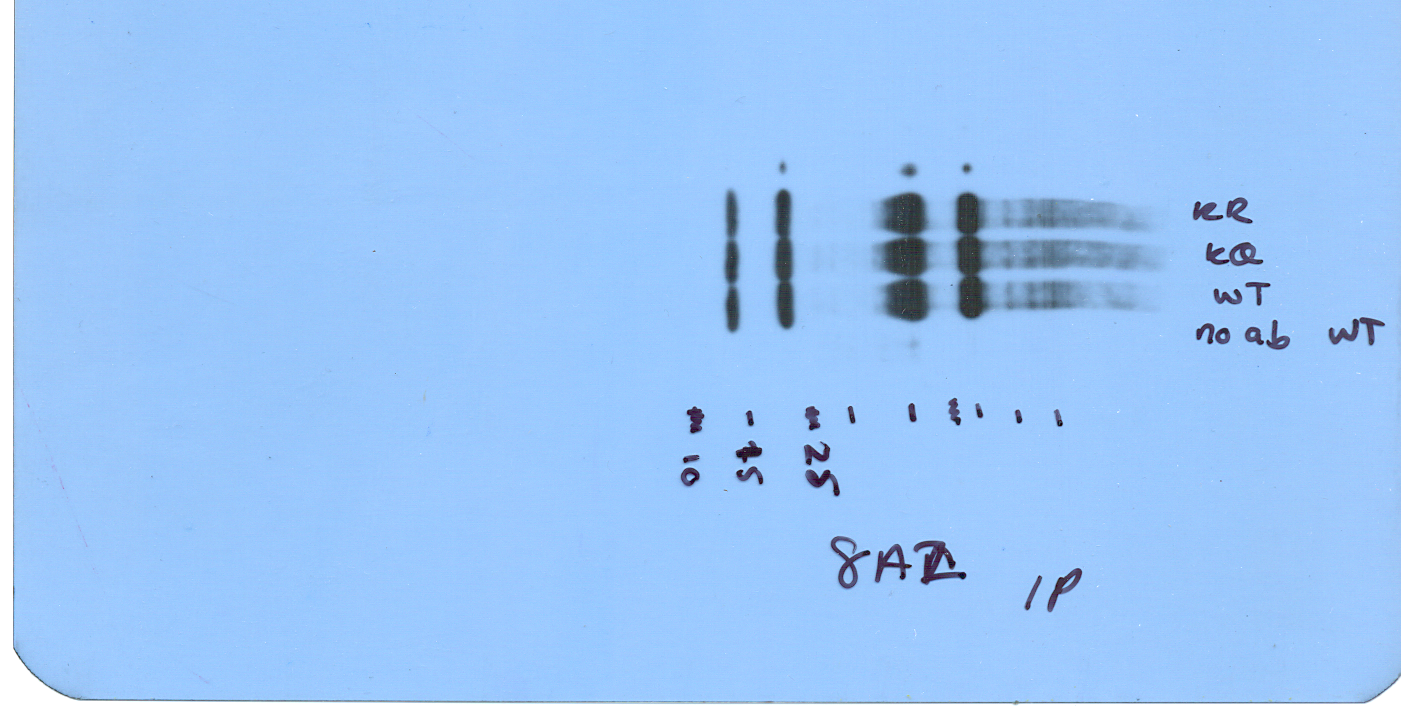

Supplement: Supplementary file 6 — Figure 3 raw data [file 44318_2025_532_MOESM6_ESM.zip › Figure 3/3A/new y299 IP/Mitotic SUMO2.3 IP probed with 8A2.tif]

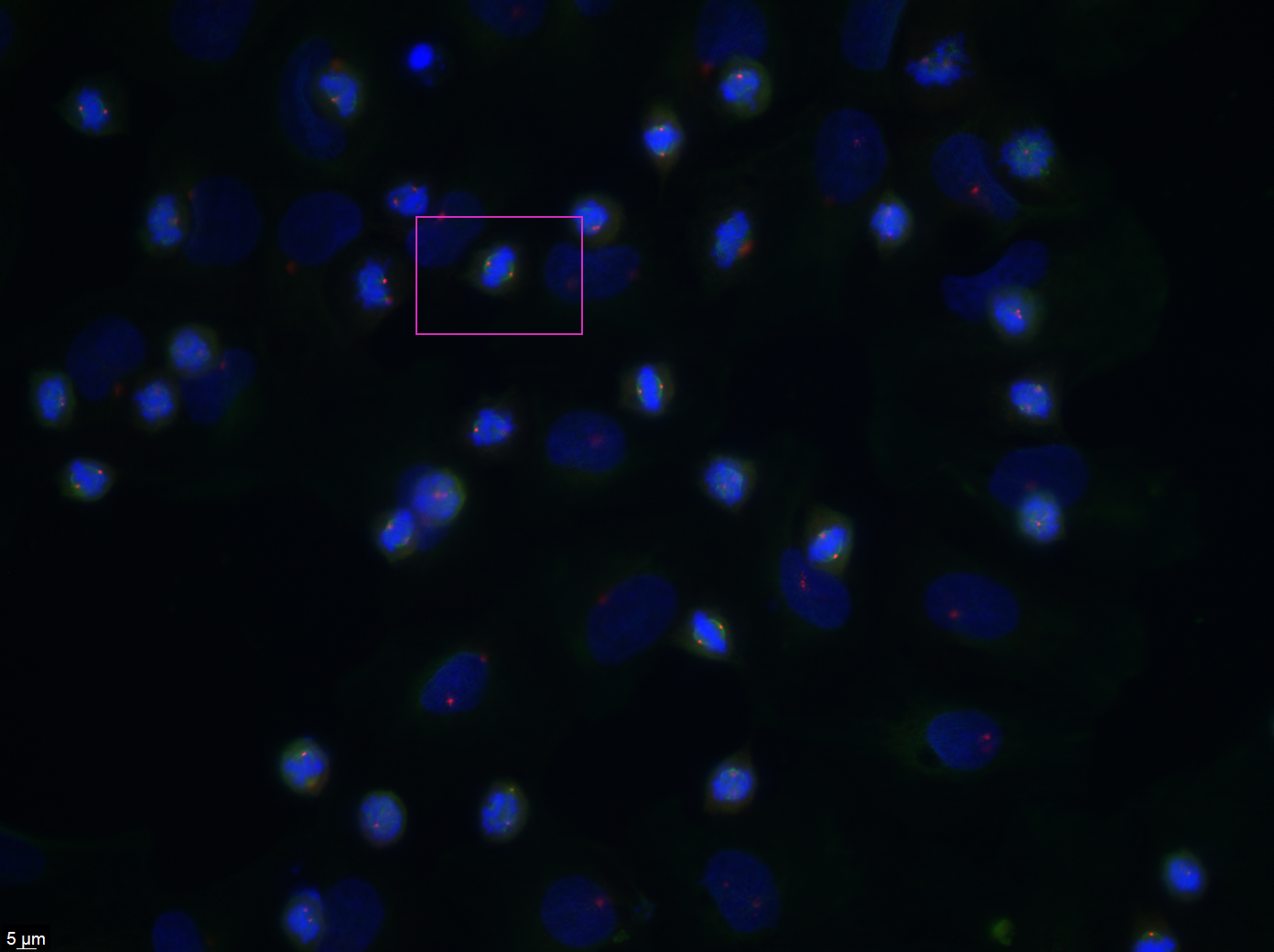

Supplement: Supplementary file 6 — Figure 3 raw data [file 44318_2025_532_MOESM6_ESM.zip › Figure 3/3B/Bipolar mitotic spindle pericentrin channel1, tubulin channel 2.tif]

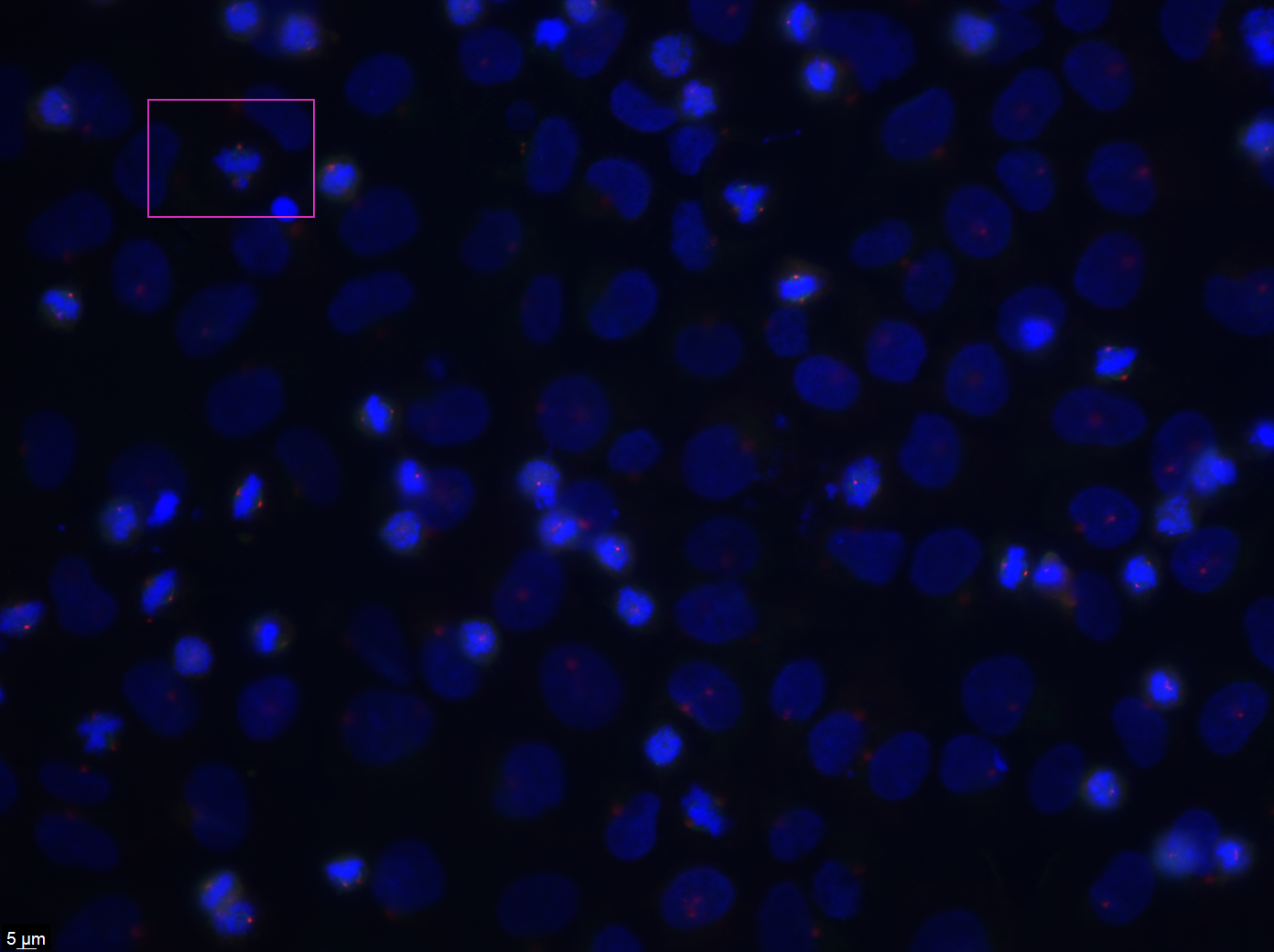

Supplement: Supplementary file 6 — Figure 3 raw data [file 44318_2025_532_MOESM6_ESM.zip › Figure 3/3B/multipolar spind pericentrin channel 1 tubulin channel 2.tif]
