# Supplementary material for: HDAC6-dependent deacetylation of SAE2 enhances SUMO1 conjugation for mitotic integrity
Source: EMBO J. 2025 Aug 20;44(19):5537–63. doi: 10.1038/s44318-025-00532-y (PMC12489036; doi:10.1038/s44318-025-00532-y)
Supplement: Supplementary file 7 — Expanded View Figures [file 44318_2025_532_MOESM7_ESM.pdf]

Expanded View Figures

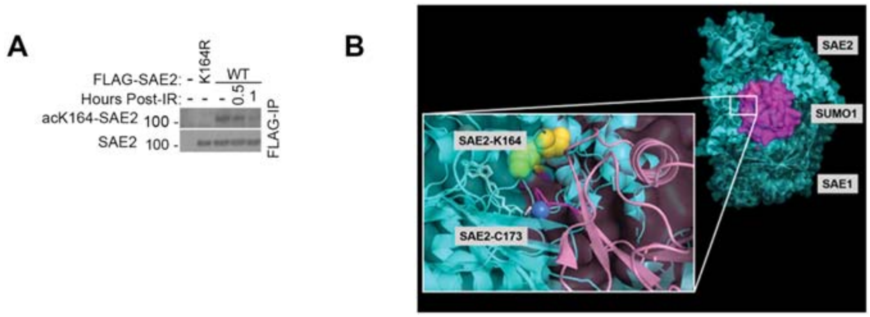

Figure EV1. Detection of acK164-SAE2 and location of SAE2-K164

(**A**) Western blot analysis of U2OS cells, untransfected (-) or expressing FLAG-SAE2 or FLAG-SAE2-K164R and treated with 10 Gy IR, then with half an hour (0.5) or 1 h recovery (1). Lysates were subjected to anti-FLAG immunoprecipitation and western blots were probed with acetyl-K164-SAE2 (mouse monoclonal) and anti-SAE2 antibodies. Performed once. (**B**) Structure of SAE1:SAE2:SUMO1 (PDB: 3KYD) adapted from Olsen et al, (2010) represented as a ribbon structure of SAE1:SAE2 in cyan and SUMO1 in magenta. The magnified image shows the C-terminal tail of SUMO1 (dark magenta) extending toward SAE2-C173 (dark blue sphere) through a channel in SAE1:SAE2; the ceiling of the channel is in part formed by SAE2-K164 (yellow spheres, visable as green where through the cyan).

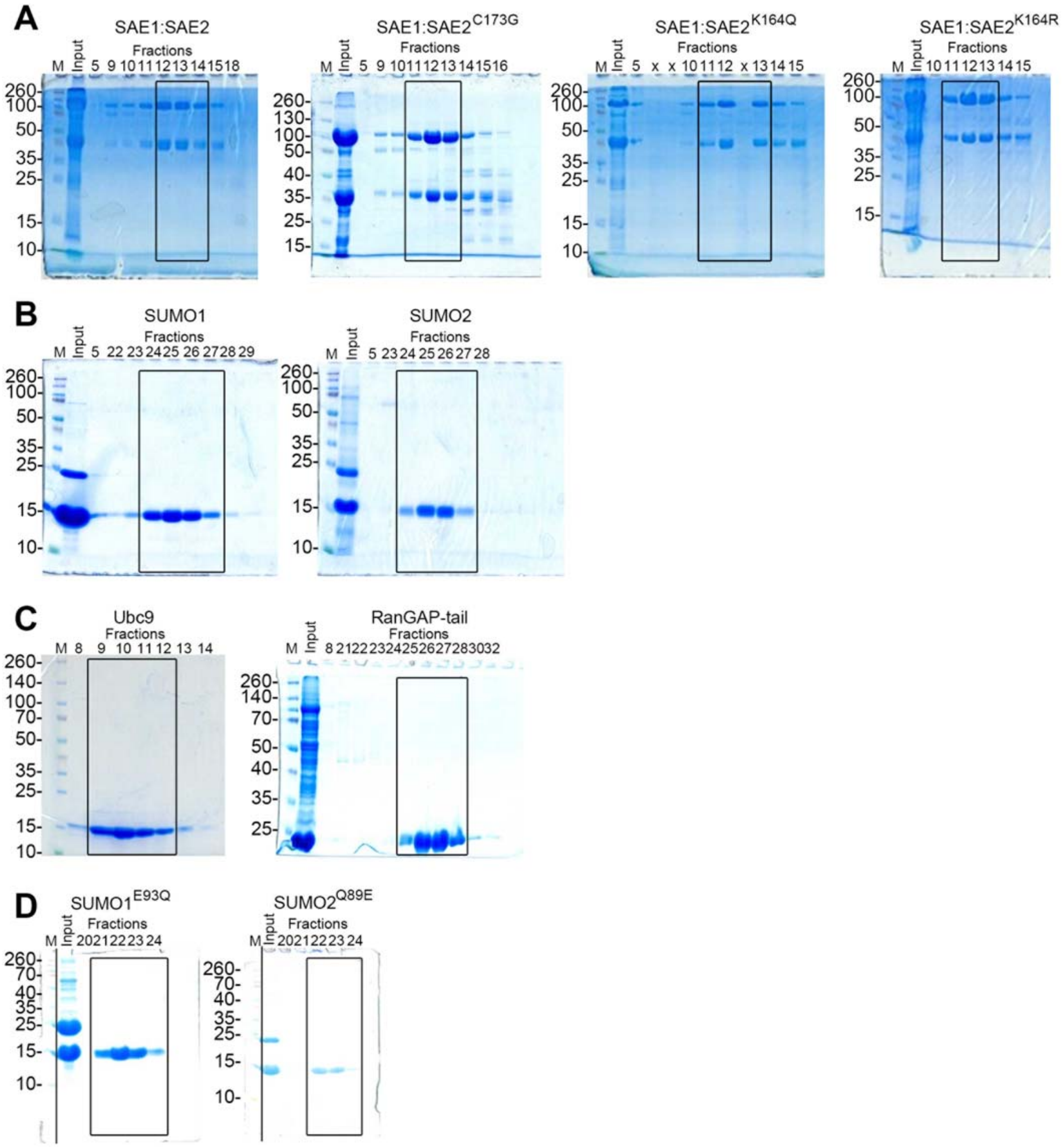

Figure EV2. Coomassie gels for each recombinant protein prepared.

Representative Instantblue stained SDS-PAGE gels from SEC fractions for the respective purified proteins. Shown here are gels from (**A**) SAE1:SAE2, SAE1:SAE2-C173G, SAE1:SAE2-K164Q, and SAE1:SAE2-K164R; (**B**) SUMO1 and SUMO2; (**C**) UBC9 and RanGAP1 (aa 398-587); and (**D**) SUMO1-E93Q and SUMO2-Q89E protein purifications.

 © The Author(s)

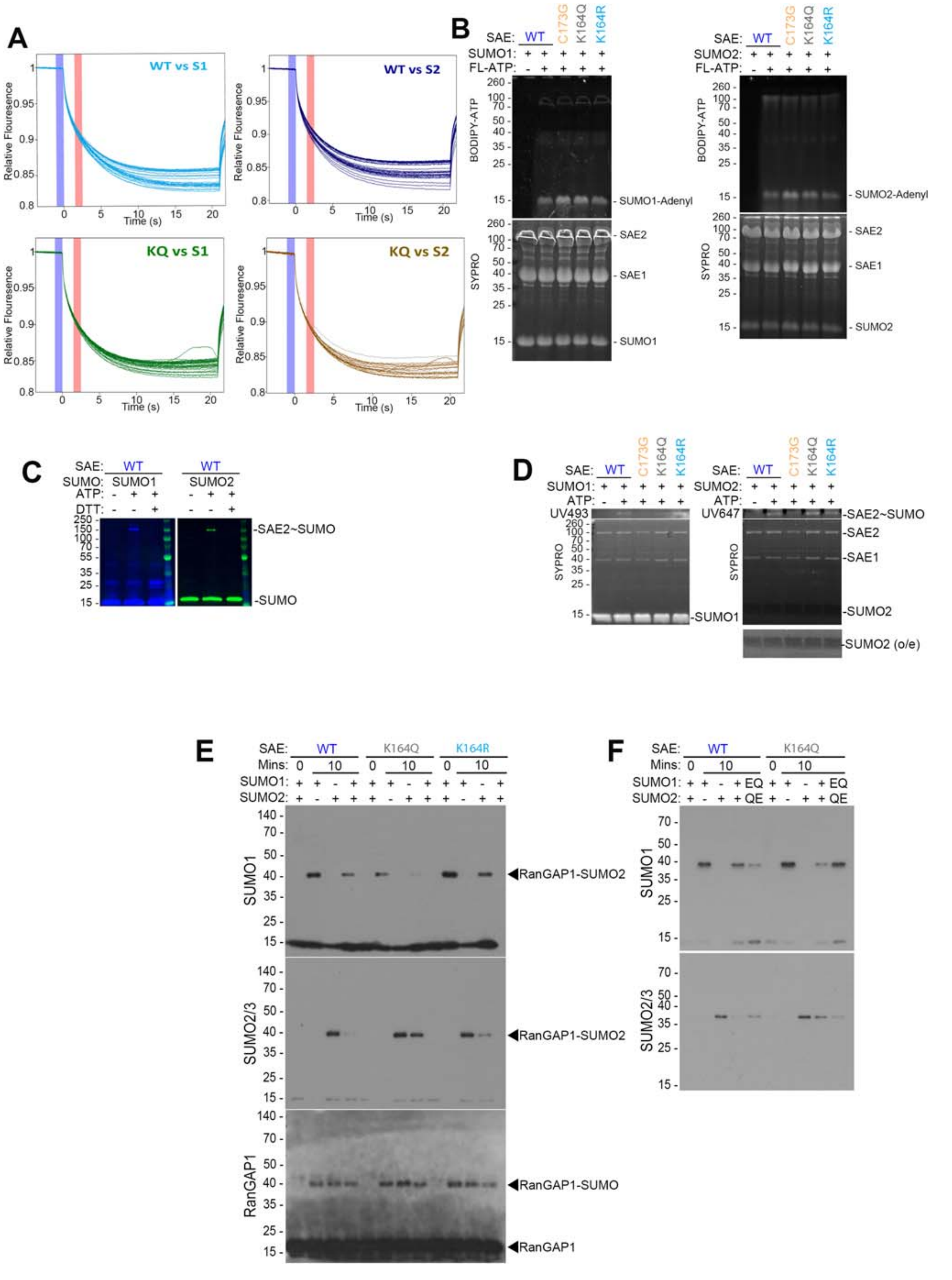
A
WT vs S1
WT vs S2
KQ vs S1
KQ vs S2
Relative Flouresence
Time (s)
B
SAE: WT C173G K164Q K164R
SUMO1: + + + + +
FL-ATP: - + + + +
BODIPY-ATP
SYPRO
- SUMO1-Adenyl
- SAE2
- SAE1
- SUMO1
SUMO2: + + + + +
- SUMO2-Adenyl
- SUMO2
C
SAE: WT WT
SUMO: SUMO1 SUMO2
ATP: - + + - + +
DTT: - - + - - +
-SAE2~SUMO
-SUMO
D
SAE: WT C173G K164Q K164R
SUMO1: + + + + +
ATP: - + + + +
UV493
UV647
-SAE2~SUMO
-SAE2
-SAE1
-SUMO1
-SUMO2
-SUMO2 (o/e)
E
SAE: WT K164Q K164R
Mins: 0 10 0 10 0 10
SUMO1: + + - + + + - + + + - +
SUMO2: + - + + + - + + + - + +
SUMO1
SUMO2/3
RanGAP1
RanGAP1-SUMO2
RanGAP1-SUMO
RanGAP1
F
SAE: WT K164Q
Mins: 0 10 0 10
SUMO1: + + - + EQ + + - + EQ
SUMO2: + - + + QE + - + + QE

Figure EV3. Extended in vitro data.

(**A**) MST Thermographs of SUMO1 or SUMO2 binding to SAE1:SAE2 or SAE1:SAE2-K164Q provide well-defined curves. The cold region is set to 0 s (blue) and the hot region set to 2.5 s (red) to determine the K_d of the interaction and to avoid any potential convection phenomena. (**B**) SDS-PAGE gels from in vitro adenylation assays after combining 30 μM SAE1:SAE2 variants, 40 μM SUMO1 (top) or SUMO2 (bottom), and 150 μM BODIPY-ATP. Gels were imaged with excitation at 488 nm to observe the BODIPY-ATP. Gels were subsequently stained using SYPRO Ruby to check protein loading. Replicated 3-times in the laboratory and quantified in Fig. 2B. (**C**) Confirmation of thioester bond formation between SAE2 and Alexaflour-tagged SUMO proteins. Reactions comprised 1 μM SUMO1-C52A-S9C-Alexa488(left) or 1 μM SUMO2-C48A-A2C-Alexa647 (right), 200 nM SAE1:SAE2, and 5 mM ATP, incubated at 30 °C for 10 min. Reactions from the left, in lanes 1 and 4 lacked ATP, and lanes 3 and 6 were followed by 30 °C, 10 min incubation at 30 °C with 100 mM DTT to assess SAE2~SUMO thioester formation. The SUMO1-C52A-S9C-Alexa488 and SUMO2-C48A-A2C-Alexa647 loading were observed with excitation wavelengths of 493 nm and 647 nm, respectively, with bands at ~120 kDa taken to be SAE2~SUMO. Replicated once in the laboratory. (**D**) Representative SDS-PAGE gels for in vitro SUMO loading assays combining 10 μM SUMO with 5 μM SAE1:SAE2-K164 variants or SAE1:SAE2-C173G. Reactions were initiated by the addition of 5 mM ATP on ice for 15 s and terminated by the addition of reducing agent-free loading buffer and boiling samples. 10 μM SUMO1-C52A-S9C-Alexa488 and SUMO2-C48A-A2C-Alexa647 were observed with excitation wavelengths of 493 nm and 647 nm, respectively, with bands at 120 kDa taken to be SAE2~SUMO. SDS-PAGE gels were stained with SYPRO ruby to detect unconjugated SUMO1 (15 kDa), SAE1 (40 kDa), and SAE2 (100 kDa). The panel below shows the over-exposed image to show SUMO2 loading. Replicated 3-times in the laboratory and quantified in Fig. 2C. (**E**) Representative blots for the in vitro SUMOylation data in Fig. 2D. Reactions comprised 10 μM SUMO1 and/or 10 μM SUMO2, 25 nM SAE1:SAE2, 100 nM UBC9, 10 μM RanGAP1 (aa 398–587), and 5 mM ATP, incubated at 30 °C for 10 min. Conditions were processed by SDS-PAGE in duplicate, such that western blots were developed using αSUMO1 and αSUMO2/3 antibodies. Note that the bands indicated are those quantified. Replicated 3 times in the laboratory and quantified in Fig. 2D. (**F**) Representative blots for the in vitro SUMOylation data in setup as described for EV3D and including a condition with 20 μM SUMO1-E93Q and SUMO2-Q89E. As before, samples were processed by SDS-PAGE in duplicate and western blots were developed using αSUMO1 and αSUMO2/3 antibodies. Replicated 3-times in the laboratory and quantified in Fig. 2F.

© The Author(s)

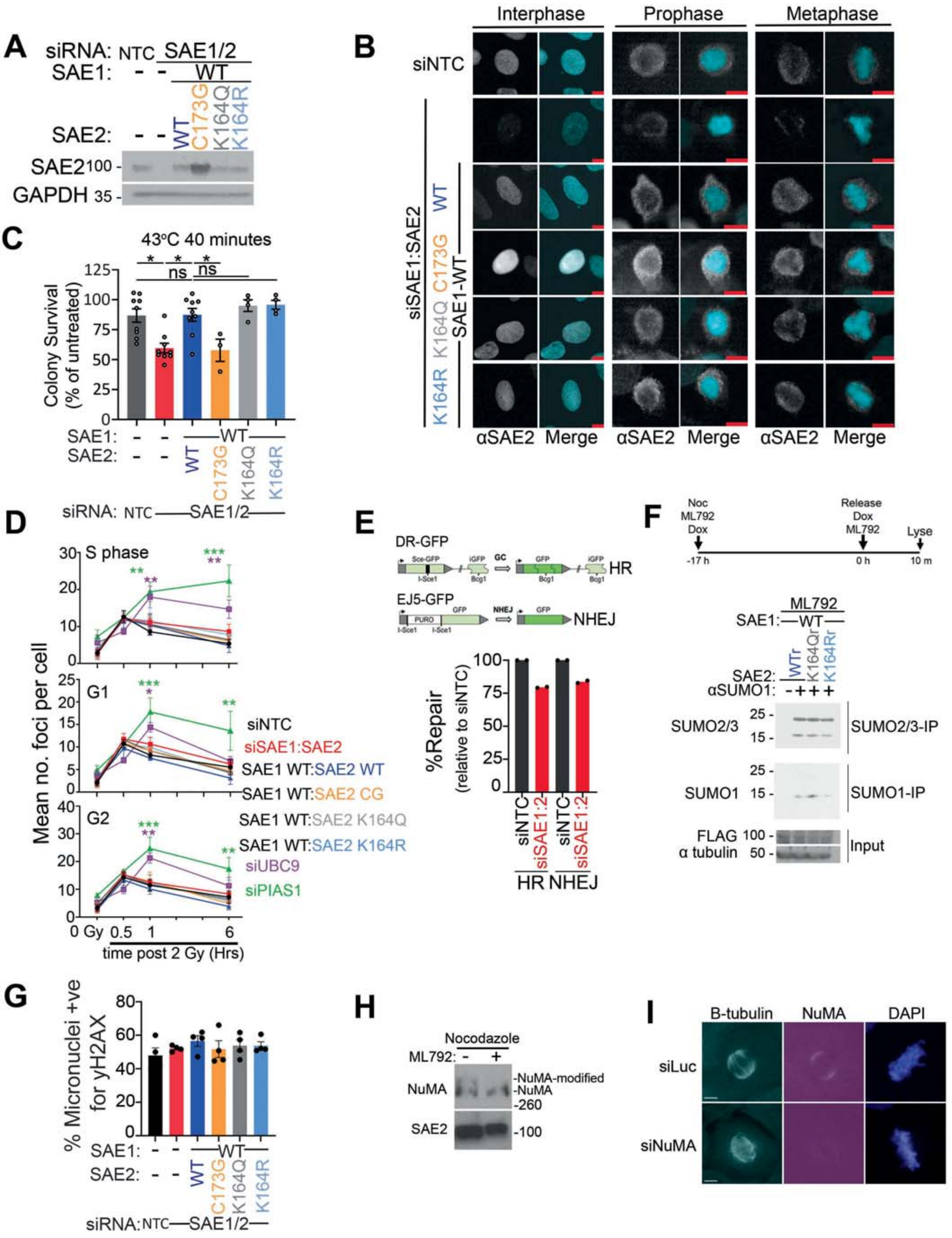
A
siRNA: NTC SAE1/2
SAE1: - - WT
SAE2: - - WT C173G K164Q K164R
SAE2 100 -
GAPDH 35 -
B
Interphase
Prophase
Metaphase
siNTC
siSAE1:SAE2
SAE1-WT
WT
C173G
K164Q
K164R
αSAE2 Merge αSAE2 Merge αSAE2 Merge
C
43°C 40 minutes
Colony Survival (% of untreated)
ns
siRNA: NTC SAE1/2
D
S phase
G1
G2
Mean no. foci per cell
siNTC
siSAE1:SAE2
SAE1 WT:SAE2 WT
SAE1 WT:SAE2 CG
SAE1 WT:SAE2 K164Q
SAE1 WT:SAE2 K164R
siUBC9
siPIAS1
time post 2 Gy (Hrs)
E
DR-GFP
EJ5-GFP
HR
NHEJ
%Repair (relative to siNTC)
siNTC siSAE1:2 siNTC siSAE1:2
F
Noc ML792 Dox
Release Dox ML792
Lyse
-17 h 0 h 10 m
ML792
SAE1: WT
SAE2: WTr K164Qr K164Rr
αSUMO1: - + + +
SUMO2/3 SUMO2/3-IP
SUMO1 SUMO1-IP
FLAG 100 -
α tubulin 50 -
Input
G
% Micronuclei +ve for γH2AX
SAE1: - - WT
SAE2: - - WT C173G K164Q K164R
siRNA: NTC SAE1/2
H
Nocodazole
ML792: - +
NuMA
SAE2
-NuMA-modified
-NuMA
-260
-100
I
B-tubulin NuMA DAPI
siLuc
siNuMA

Figure EV4. Extended assessment of the cellular impacts of SAE2 variants.

(**A**) Representative western blot of SAE2 expression in stable, inducible siRNA-resistant SAE1:SAE2 variant U2OS cells. Cells were treated for 72 h with siRNA for either NTC or SAE1:SAE2 with the concurrent addition of 4 µg/ml Doxycycline to induce expression of the indicated integrated SAE1:2 constructs. Replicated >5 times in the laboratory. (**B**) Representative images depicting the localisation of SAE2 in interphase, prophase and metaphase U2OS cells. Cells depleted for SAE1:SAE2 and complemented with WT or indicated SAE1:SAE2-variants. Interphase cells received no synchronisation. Prophase and metaphase cells were synchronised in nocodazole for 16 h and either fixed immediately or after a 35 min release into mitosis, respectively. Five-micrometer scale bar is shown as a red line. Cells chosen from a representative field >20 similar cells. Performed once. (**C**) U2OS depleted for SAE1:SAE2 and complemented with WT or indicated SAE1:SAE2-variants, subjected to 43 °C for 40 min before replating and counting after colony growth. Significance calculated using one-way ANOVA. Error bars = SEM; $N = 3$ biological repeats. * = $P \leq 0.05$, ns = not significant $p > 0.05$. Statistical values for NTC vs siSAE $P = 0.0060$, siSAE vs siSAE+SAE2-WT $P = 0.0064$, NTC vs siSAE+SAE2-WT $P > 0.9999$, siSAE+SAE2-WT vs siSAE+SAE2-C173G $P = 0.0174$, siSAE+SAE2-WT vs siSAE+SAE2-K164Q $P = 0.9665$, siSAE+SAE2-WT vs siSAE+SAE2-K164R $P = 0.4874$. (**D**) Automated analysis of γH2AX foci numbers, obtained through high-content microscopy, in U2OS cells treated with indicated siRNAs (siNTC- black, siSAE1:SAE2- red) with or without the complementation of inducible siRNA-resistant SAE2 variants (SAE2 WT- dark blue, SAE2 CG- orange, SAE2 K164Q- grey, SAE2 K164- light blue). siUBC9 (purple) and siPIAS1 (green) are used for comparison. Results displayed for data isolated from S phase (top), G1 (middle) and G2 (bottom) cell populations. Plotted data is derived from the mean number of foci per condition from 3 independent biological repeats, error bars = SEM. Statistical significance was calculated using two-way ANOVA using Dunnett's multiple comparisons test. Timepoints where there is a significant difference from the non-target control siRNA condition are marked with * = $P < 0.05$, ** = $P < 0.01$, *** = $P < 0.001$. Purple and green * show that only siUBC9 and siPIAS1 conditions significantly deviate from siNTC at points in the time course. siNTC vs siSAE1:SAE2 (S phase 1 h $P = 0.8997$, 6 h $P = 0.7437$; G1 1 h $P = 0.7623$, 6 h $P = 0.9997$; G2 1 h $P = 0.9993$, 6 h $P = 0.9988$), siNTC vs SAE2-WT (S phase 1 h $P = 0.9792$, 6 h $P > 0.9999$; G1 1 h $P = 0.9997$, 6 h $P = 0.7655$; G2 1 h $P = 0.9971$, 6 h $P = 0.7660$), siNTC vs SAE2-CG (S phase 1 h $P = 0.9907$, 6 h $P > 0.9999$; G1 1 h $P = 0.9965$, 6 h $P = 0.9989$; G2 1 h $P = 0.9957$, 6 h $P = 0.9494$), siNTC vs SAE2-KQ (S phase 1 h $P = 0.9686$, 6 h $P = 0.9993$; G1 1 h $P = 0.9978$, 6 h $P = 0.9932$; G2 1 h $P > 0.9999$, 6 h $P > 0.9999$), siNTC vs SAE2-KR (S phase 1 h $P = 0.8283$, 6 h $P > 0.9211$; G1 1 h $P = 0.9430$, 6 h $P = 0.9799$; G2 1 h $P = 0.9999$, 6 h $P = 0.9967$), siNTC vs siUBC9 (S phase 1 h $P = 0.0076$, 6 h $P = 0.0086$; G1 1 h $P = 0.0277$, 6 h $P = 0.9833$; G2 1 h $P = 0.0073$, 6 h $P = 0.5598$), siNTC vs siPIAS1 (S phase 1 h $P = 0.0016$, 6 h $P < 0.0001$; G1 1 h $P = 0.0002$, 6 h $P = 0.0020$; G2 1 h $P = 0.0002$, 6 h $P = 0.0051$). (**E**) The measure of DNA repair from U2OS cells bearing integrated DNA repair reporters in cells treated with siNTC or siSAE1:siSAE2 and transfected with the enzyme, I-SCE-1. Illustration of the integrated DNA repair substrates for homologous recombination and non-homologous end-joining (Top). The graph (Bottom) displays the percentage of GFP-positive cells normalised to RFP-transfection efficiency. %-repair of siSAE1:SAE2 is given relative to siNTC. Data from 2 independent biological repeats. (**F**) Immunoprecipitation of endogenous mitotic SUMO conjugates in U2OS cells treated with ML792 and expressing Flag-SAE2 constructs resistant to the inhibitor. ML792 resistance is denoted by (r). The presence of Flag-SAE2r is represented by (WTr), Flag-SAE2r-K164Q by KQr, and Flag-SAE2r-K164R by KRr. The diagram, top, illustrates the timing of inhibitor and induction agent addition. To better detect free SUMO, Y299 (SUMO1) and 8A2 (SUMO2/3) antibodies were employed (Garvin et al, 2022). Performed once. (**G**) The percentage of micronuclei positive for γH2AX in asynchronous siRNA-resistant SAE2 variant-expressing U2OS cells. Error bars SEM. Data from 4 independent biological repeats. $N > 50$ micronuclei per condition per biological repeat. Significance was tested using one-way ANOVA no significant differences between conditions were identified. (**H**) Western blot analysis of U2OS treated with nocodazole ±5 µM ML792 for 16 h. Mitotic cells were harvested by mitotic shake-off and lysed in loading buffer, and western probed for NuMA and SAE2. Performed twice. (**I**) Representative images validating the specificity of the NuMA antibody. NuMA colocalises to -β tubulin metaphase cells adjacent to the DAPI stain. NuMA signal significantly diminished after 72-h 10 nM siNuMA treatment. Cells chosen from >50 similar cells, performed once. White bar indicates 5 micrometers.

 © The Author(s)

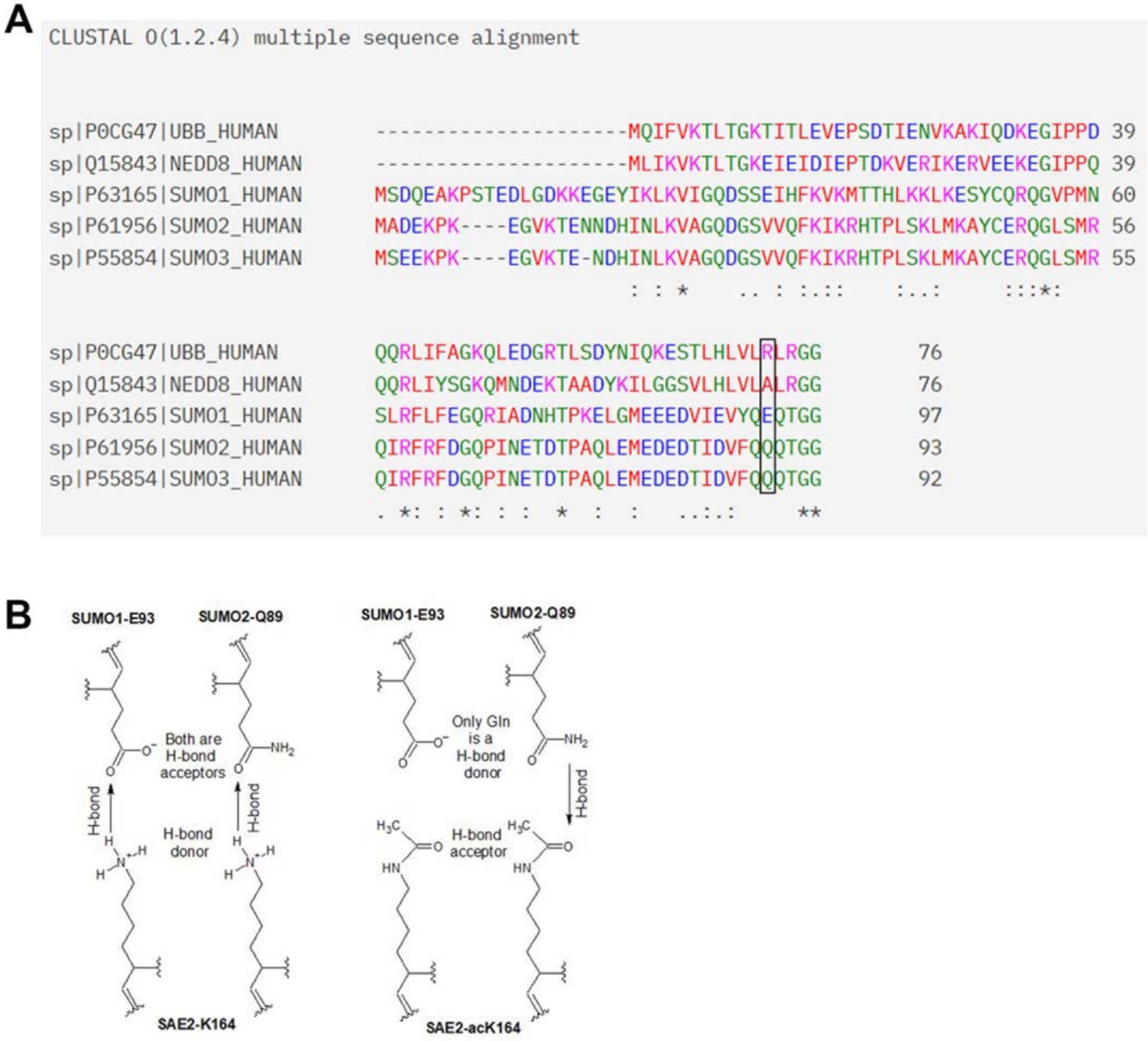

Figure EV5. Discrimination between SUMO1 and SUMO2 by SAE2-K164.

(**A**) Amino acid sequence alignment for Ubiquitin (UBB), Nedd8, SUMO1, SUMO2, and SUMO3 up to the C-terminal di-Gly motif representative of mature activation/conjugation-competent Ubls. The black box indicates Ubiquitin-R72, which is divergent in Nedd8, SUMO1, and SUMO2/3 and required for Ubl E1 discrimination of different Ubl modifiers (Walden et al, 2003). Generated using Uniprot and Clustal Omega. (**B**). Illustration of one proposed mechanism of SAE1:SAE2-acK164 bias for SUMO2-Q89 over SUMO1-E93 through hydrogen bonding patterns in the SUMO E1 'closed' conformation. Unacetylated SAE2-K164 acts as a hydrogen bond donor to both SUMO1-E93 or SUMO2-Q89, SAE2-acK164 acquires hydrogen bond acceptor capacity to which SUMO2-Q89 donate a hydrogen bond, while SUMO1-E93 cannot. This hypothetical hydrogen bonding arrangement explains why SAE1:SAE2-acK164 bears a bias towards SUMO2 activation. A second, additive model, is that of electrostatic interaction between SAE2-K164 and SUMO1-E93, which is absent for K164-SAE2:Q89-SUMO2 and lost for acK164-SAE2:E93-SUMO1/Q89-SUMO2 context.

© The Author(s)
